# Supplementary material for: Exploring climate smart agriculture in Turkey: Enhancing food security and sustainable practices for the reduction of CO₂ emissions
Source: PLoS One. 2026 Mar 31;21(3):e0344924. doi: 10.1371/journal.pone.0344924 (PMC13037976; doi:10.1371/journal.pone.0344924)

## Appendix A

<https://data.worldbank.org/topic/agriculture-and-rural-development> and

https://ourworldindata.org/search?q=agriculture

## Appendix B


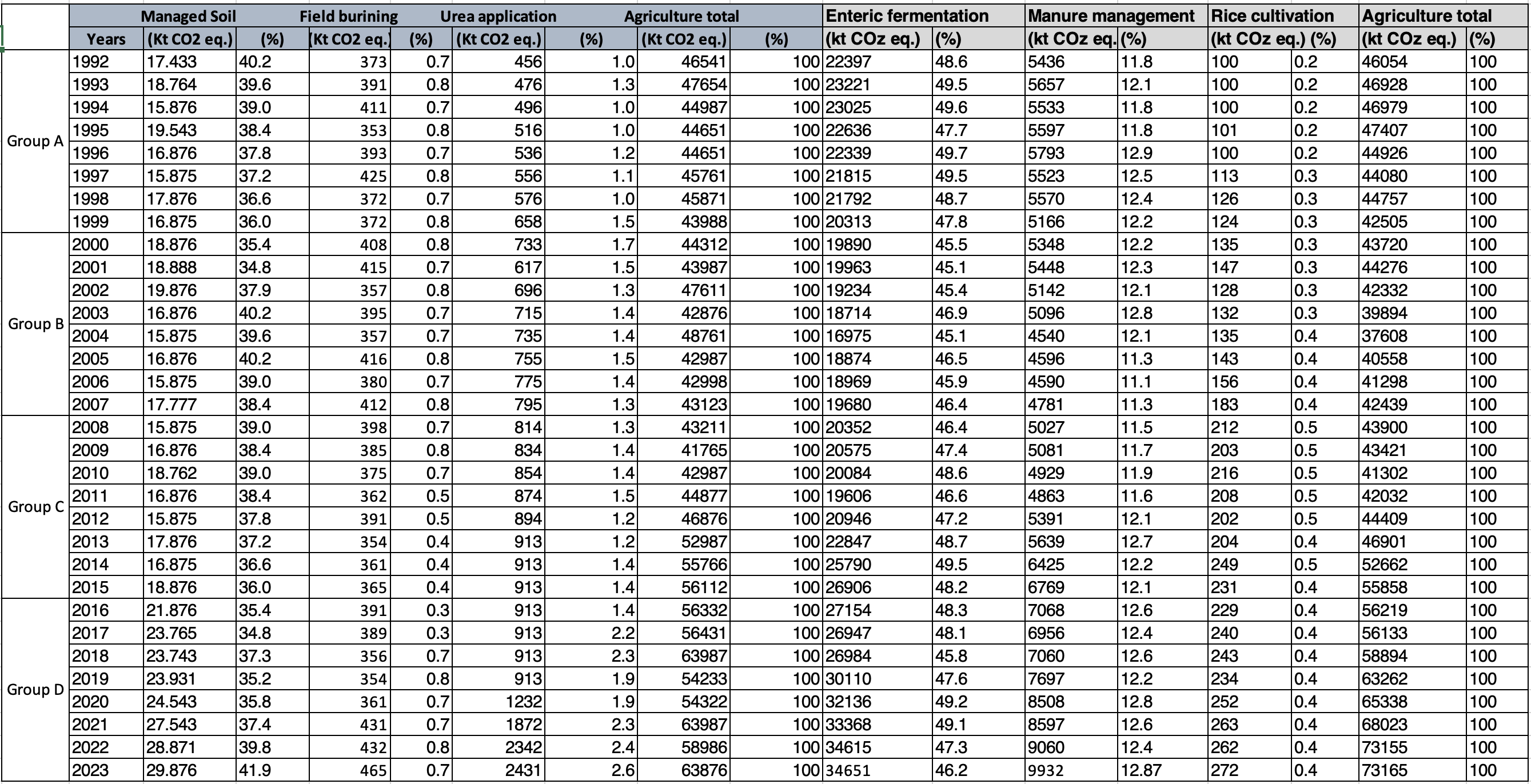

Supplement: S1 File — (DOCX) [file pone.0344924.s001.docx]
