# Supplementary material for: Exploring climate smart agriculture in Turkey: Enhancing food security and sustainable practices for the reduction of CO₂ emissions
Source: PLoS One. 2026 Mar 31;21(3):e0344924. doi: 10.1371/journal.pone.0344924 (PMC13037976; doi:10.1371/journal.pone.0344924)
Supplement: Attachment — Submitted filename: PONE-D-25-27665_ Reviewed.pdf [file pone.0344924.s003.pdf]

PLOS ONE

Exploring Climate Smart Agriculture in Turkey: Enhancing Food Security and Sustainable Practices for the Reduction of CO2 Emissions

--Manuscript Draft--

**Manuscript Number:**	PONE-D-25-27665
**Article Type:**	Research Article
**Full Title:**	Exploring Climate Smart Agriculture in Turkey: Enhancing Food Security and Sustainable Practices for the Reduction of CO2 Emissions
**Short Title:**	Climate Smart Agriculture
**Corresponding Author:**	Nawaz Khan, PhD<br>Fujian University of Technology<br>CHINA
**Keywords:**	Climate Smart-Agriculture; portfolio return, environment; CO2 emissions; Turkey
**Abstract:**	Climate smart agriculture, which entails the reduction of CO2 emissions, the adaptation and modification of technology to enhance resilience to climate change, and the sustainable increase of incomes. To assess the efficacy of smart agriculture practices, which significantly enhance food security and reduce CO2 emissions, this investigation implements the decoupling method. The decoupling process is responsible for estimating the percentage change in portfolio returns with high and low weighted risk over the projected five-year period from 1992 to 2023. The estimated results of decoupling CO2 emissions are subject to a 1% variation in rice cultivation, field fire, and managed soil. Rice cultivation and soil management are the factors that contribute to the highest weighted risk. The sustainable smart agriculture process in Turkey is crucial for the effective growth of the economy, as it integrates climate change considerations into existing policies and initiatives and supports the policy indicator of economic consideration with environmental protection in terms of CO2 emissions.
**Order of Authors:**	Wang Jie
	Nawaz Khan, PhD
**Additional Information:**	
**Question**	**Response**
**Financial Disclosure**<br><br>Enter a financial disclosure statement that describes the sources of funding for the work included in this submission. Review the submission guidelines for detailed requirements. View published research articles from *PLOS ONE* for specific examples.<br><br>This statement is required for submission and **will appear in the published article** if the submission is accepted. Please make sure it is accurate.	The author(s) received no specific funding for this work.

Powered by Editorial Manager® and ProduXion Manager® from Aries Systems Corporation

**Funded studies**<br>Enter a statement with the following details:<br>• Initials of the authors who received each award<br>• Grant numbers awarded to each author<br>• The full name of each funder<br>• URL of each funder website<br>• Did the sponsors or funders play any role in the study design, data collection and analysis, decision to publish, or preparation of the manuscript?<br><br>Did you receive funding for this work?	
**Competing Interests**<br><br>Use the instructions below to enter a competing interest statement for this submission. On behalf of all authors, disclose any competing interests that could be perceived to bias this work—acknowledging all financial support and any other relevant financial or non-financial competing interests.<br><br>This statement is required for submission and **will appear in the published article** if the submission is accepted. Please make sure it is accurate and that any funding sources listed in your Funding Information later in the submission form are also declared in your Financial Disclosure statement.<br><br>View published research articles from *PLOS ONE* for specific examples.	The authors have declared that no competing interests exist.

Powered by Editorial Manager® and ProduXion Manager® from Aries Systems Corporation

**NO authors have competing interests**<br><br>Enter: *The authors have declared that no competing interests exist.*<br><br>**Authors with competing interests**<br><br>Enter competing interest details beginning with this statement:<br><br>*I have read the journal's policy and the authors of this manuscript have the following competing interests: [insert competing interests here]*<br><br>* typeset	
**Ethics Statement**<br><br>Enter an ethics statement for this submission. This statement is required if the study involved:<br><br>• Human participants<br>• Human specimens or tissue<br>• Vertebrate animals or cephalopods<br>• Vertebrate embryos or tissues<br>• Field research<br><br>Write "N/A" if the submission does not require an ethics statement.<br><br>General guidance is provided below. Consult the submission guidelines for detailed instructions. **Make sure that all information entered here is included in the Methods section of the manuscript.**	N/A

Powered by Editorial Manager® and ProduXion Manager® from Aries Systems Corporation

**Format for specific study types**<br><br>**Human Subject Research (involving human participants and/or tissue)**<br>• Give the name of the institutional review board or ethics committee that approved the study<br>• Include the approval number and/or a statement indicating approval of this research<br>• Indicate the form of consent obtained (written/oral) or the reason that consent was not obtained (e.g. the data were analyzed anonymously)<br><br>**Animal Research (involving vertebrate animals, embryos or tissues)**<br>• Provide the name of the Institutional Animal Care and Use Committee (IACUC) or other relevant ethics board that reviewed the study protocol, and indicate whether they approved this research or granted a formal waiver of ethical approval<br>• Include an approval number if one was obtained<br>• If the study involved *non-human primates*, add *additional details* about animal welfare and steps taken to ameliorate suffering<br>• If anesthesia, euthanasia, or any kind of animal sacrifice is part of the study, include briefly which substances and/or methods were applied<br><br>**Field Research**<br><br>Include the following details if this study involves the collection of plant, animal, or other materials from a natural setting:<br>• Field permit number<br>• Name of the institution or relevant body that granted permission	
**Data Availability**<br><br>Authors are required to make all data underlying the findings described fully available, without restriction, and from the time of publication. PLOS allows rare exceptions to address legal and ethical concerns. See the PLOS Data Policy and FAQ for detailed information.	Yes - all data are fully available without restriction

Powered by Editorial Manager® and ProduXion Manager® from Aries Systems Corporation

A Data Availability Statement describing where the data can be found is required at submission. Your answers to this question constitute the Data Availability Statement and **will be published in the article**, if accepted.<br><br>**Important:** Stating 'data available on request from the author' is not sufficient. If your data are only available upon request, select 'No' for the first question and explain your exceptional situation in the text box.<br><br>Do the authors confirm that all data underlying the findings described in their manuscript are fully available without restriction?	
**Describe where the data may be found in full sentences. If you are copying our sample text, replace any instances of XXX with the appropriate details.**<br><br>• If the data are **held or will be held in a public repository**, include URLs, accession numbers or DOIs. If this information will only be available after acceptance, indicate this by ticking the box below. For example: *All XXX files are available from the XXX database (accession number(s) XXX, XXX.).*<br>• If the data are all contained **within the manuscript and/or Supporting Information files**, enter the following: *All relevant data are within the manuscript and its Supporting Information files.*<br>• If neither of these applies but you are able to provide **details of access elsewhere**, with or without limitations, please do so. For example:<br><br>*Data cannot be shared publicly because of [XXX]. Data are available from the XXX Institutional Data Access / Ethics Committee (contact via XXX) for researchers who meet the criteria for access to confidential data.*<br><br>*The data underlying the results presented in the study are available from (include the name of the third party*	n/a

and contact information or URL).<br>• This text is appropriate if the data are owned by a third party and authors do not have permission to share the data.<br><br>* typeset	
Additional data availability information:	Tick here if the URLs/accession numbers/DOIs will be available only after acceptance of the manuscript for publication so that we can ensure their inclusion before publication.; Tick here if your circumstances are not covered by the questions above and you need the journal's help to make your data available.

Manuscript Click here to access/download;Manuscript;Manuscript-2025-05-22.docx

Exploring Climate Smart Agriculture in Turkey: Enhancing Food Security and Sustainable Practices for the Reduction of CO_2 Emissions

Abstract

Climate smart agriculture, which entails the reduction of CO_2 emissions, the adaptation and modification of technology to enhance resilience to climate change, and the sustainable increase of incomes. To assess the efficacy of smart agriculture practices, which significantly enhance food security and reduce CO_2 emissions, this investigation implements the decoupling method. The decoupling process is responsible for estimating the percentage change in portfolio returns with high and low weighted risk over the projected five-year period from 1992 to 2023. The estimated results of decoupling CO_2 emissions are subject to a 1% variation in rice cultivation, field fire, and managed soil. Rice cultivation and soil management are the factors that contribute to the highest weighted risk. The sustainable smart agriculture process in Turkey is crucial for the effective growth of the economy, as it integrates climate change considerations into existing policies and initiatives and supports the policy indicator of economic consideration with environmental protection in terms of CO_2 emissions.

Keywords: Climate Smart-Agriculture; portfolio return, environment; CO2 emissions; Turkey

1. Introduction

Climate smart agriculture (CLS-A) reduces CO2 emission by lowering the emission of CO2 from agricultural activities and by helping to sequester CO2 from the atmosphere [1]. Cover cropping practices which sequester and add CO2 into the year end soil organic matter and enhanced grazing management which increases carbon storage in CLS-A [2]. First, Turkey is very susceptible initially to environmental changes [3]. It is in the Mediterranean basin, where increases in temperature as well as droughts due to extreme weather are already happening [4, 5]. Furthermore, CLS-A helps to shape economy and uses sustainability in agriculture. As such, CLS-A practices resilience and buffer environmental change, making food security and a stable economy possible. Secondly, the huge role of GHG emissions (including CO_2 emission [6]. By taking CLS-A strategic measures that will reduce such emissions and support soil management and agroforestry, Turkey can make a big contribution to its effort to meet and transition to a greener economy. In this case, the decoupling of CLS A development and rapid production from harmful processes of the environment is obtained. Correspondingly, it provides a framework for Turkey to modernize and reinforce its CLS-A sector and thereby benefit environment and economy [7, 8]. I examine th e CLS-A consequence in environmental protection by way of agroforestry — the integration of trees into agricultural systems, achieving twin goals: limiting forest denudation and sequestering atmospheric CO2 in biomass and soil [9, 10]. Environmental policies related to CO2 emission applied to Turkey's agricultural sector have been studied [11].

The two CLS-A practice primary objectives are climate change mitigation and its sustainability. [12, 13]. The CLS-A practice has two primary objectives, to minimize climate change and then maintain the sustainability of this aim [13, 14]. The other was the sustainable agriculture practice focusing on production methods which can diminish CO2 emissions. Moreover, the two main contributions demonstrated, by means of the application of decoupling techniques, the important contribution to CO2 emissions as well as the vulnerability of climate change[15, 16]. This research argues that by decoupling, Turkey can meet its food security goals with limited environment impacts [17]. To investigate and implement effective decoupling techniques, we need a multi prong approach that considers such technological developments. It may be possible to decouple these goals: By increasing food production, we can stop our environmental impact [18]. There are many ways to do this. Second, agricultural practices such as enhanced agricultural practices including integrated pest management, conservation tillage and precision CLS-A, have been tested to increase the crop yield while reducing water consumption, soil erosion and dependence of pesticides, [19]. Second, eco friendly innovations: water for irrigation can be sourced from salt water, drought resistant crops are grown and a vertical farming approach is adopted which will technically work around the constraints of available resources and lead to more efficient food production.

Third, there is a lot of wasted food because of the supply chain. Reducing food waste from farm to fork is one approach that can aid with decoupling. Lastly, a significant portion of the food waste occurs throughout the supply chain. Decoupling direct payments in Turkey's agricultural sector may significantly contribute to enhancing food security and reducing the sector's CO_2 emissions impact, according to the findings of this study. Decoupling can foster multifarious, sustainable, and fruitful agricultural methods by empowering producers to make decisions more in line with market forces. Turkey would benefit from implementing supplementary policies that encourage producers to cultivate crops using sustainable methods, thereby slowing down the process. Transitioning to a decoupled direct payment system could be a significant improvement for Turkey in addressing the challenges of food security and high levels of CO_2 emissions [20]. This CLS-A research aims to improve low-cost, long-term

farming systems. It could involve finding new sustainable food buyers or verifying fair trade [21]. Rural education, healthcare, and infrastructure are more accessible with decoupling. A step toward decoupling is the implementation of waste-reduction techniques all the way from the farm to the table. These are things Turkey can do to improve the world and secure its food supply for the future [22, 23].

This study emphasizes CLS-A's climate change adaptation through research. It underlines the need to focus on relevant research areas like drought management, climate change effects, adaptation, and evidence-based food security. It also emphasizes the need to collect and verify climate change data in each river basin and region. Authorities can affect future event preparations after accounting for the data. Research and development aims to improve land, water, and crop management in response to climate change. Thus, the literature emphasizes the following ideas and actions needed to reach this goal. This study emphasizes the need to study how climate change may affect CLS-A output, production, and local information. The main goal is to monitor climate change and create measures to limit its effects on agriculture. This study examines whether sustainable farming might reduce climate change's impact on Turkish food production. We investigate Turkey's environmental and economic policies using critical economic facts, global choices, and laws. According to studies, Turkish farmers can slow global warming by using modern farming methods. The legal application of Turkey's program reduces hazardous emissions, boosting economic growth and environmental security. Economic data can illustrate how financially feasible ideas are.

This study has two sections: background information and research on decoupling's environmental implications. Section 3 describes the research goals, theoretical and conceptual frameworks, and methodology and results. Estimation and results are in Section 4. Section 5 addresses results. The conclusion concludes the investigation and suggests policies.

2. Literature Review

2.1. Climate Smart Agriculture

A number of sustainable CLS-A-related policies and projects have been established by the Turkish government. To make these policies and initiatives work, though, more work is required. In order to create new sustainable agricultural technology, the agricultural sector must also engage in research and development. How Turkey deals with its CO_2 emissions problems will determine the fate of its CLS-A economy. More sustainable methods can help Turkey's CLS-A endure the impacts of climate change, water scarcity, and soil degradation. Consequently, this sector will persist in the future, guaranteeing that the Turkish populace has an adequate supply of sustenance.

Currently, there is insufficient information available to determine whether various decoupling strategies are the most effective in mitigating the impact of climate change on CLS-A. Furthermore, it is imperative to conduct additional research in order to determine the most cost-effective decoupling solutions. As long as the solutions are within reasonable costs, individuals who are responsible for molding policies and farmers can identify the methods that effectively capture the most CO_2 emissions [24]. It is imperative to investigate the factors that influence individuals' decisions to adopt ecological and dining behaviors. [25]. Research is necessary to develop effective laws that promote environmental protection, prevent detrimental actions, and ensure equal opportunity in agriculture. Additionally, research is necessary to investigate the consequences of policies that are currently in effect and planned for the future [26].

2.2. Sustainable practice of Turkey and CO2 emissions

Turkey can address these challenges and fill these knowledge gaps by implementing a successful decoupling strategy that is financially feasible, socially acceptable, and sustainable. It guarantees the availability of food for individuals and guarantees the sustainability of food systems in the future. In spite of the adoption of CLS-A, there are still certain research areas in CLS-A that require improvement to achieve a more favorable outcome and acceptance. Currently, there are numerous critical regions in the globe [26]. It is imperative that we determine whether CLS-A is economically feasible for small and medium-sized farms in Turkey [27]. The implementation of a program that assists farmers in enhancing their digital literacy is necessary to enable them to implement sustainable practices that leverage new technologies two successful policy instruments that can be used to encourage farmers to adopt sustainable practices [28] are subsidies and tax breaks, which are informed by research [29]. In order to incentivize producers to embrace market mechanisms, sustainable practices, such as certification systems or premium pricing for sustainably produced food could be investigated. Filling these knowledge shortfalls in agricultural sustainability can hasten Turkey's shift to ecologically friendly farming practices [30].

3. Methodology

3.1. The CLS-A Method and Decoupling

A dynamic development model of Turkey's global economy was employed in this segment to examine the interplay between food security and the environment. In this section, we analyzed decoupling as a potential solution to improve food security and reduce CO_2 emissions in Turkey. What follows is an examination of decoupling, and after that we go on to estimating portfolio return using weighted risk and return. Finally, we will discuss the fluctuation of this return. [31]. The negative portfolio return value represents the separated decoupling values. Consequently, for each category, the returns of the portfolio can be examined for a complete eight years [32]. The last step is to examine the variation in portfolio returns for every group. The next four steps make up the computation. At the onset, we make use of beta decoupling in order to ascertain the average transformation of the environment as well as the percentage change for each group individually (covariance/variance multiply with 8/7 and 1%, respectively). The 1% variation in decoupling has caused a 1% shift in the following areas: rice cultivation, enteric fermentation, field fire, managed soil, urea application, and manure management. Second, upon decoupling, we calculate the overall risk and average return for each individual group [33, 34].

The mean return provides insight into the conventional assessment, while the total risk indicates the standard deviation for each group over a period of eight years. Furthermore, the portfolio return with weighted risk is computed by adding the 100% risk premium to the adjusted return indicators of reduced environment, namely field fire, managed soil, urea application, manure management, enteric fermentation, and rice cultivation, subsequent to the average return and total risk [35, 36]. A 1 percent change in the environment is then computed using the projected weighted return and risk of each cohort. In Section 3, the effects of the environment and the impact of a 1% change in food security on CO_2 emissions degradation are also evaluated individually. Moreover, the decoupling shift at different junctures is ascertained by the groups [37]. For instance, a 1% shift in environment decoupling can be attributed to modifications in CLS-A, which encompass enteric in the field burning, managed soil, urea application, manure management, enteric fermentation, and rice cultivation [38].

3.2. Sources of the Dataset

From 1992 to 2023, this study examines the CO_2 emissions impacts of several agricultural practices in Turkey, including field burning, managed soil, urea application, manure management, enteric fermentation, and rice farming. The decoupling operation divides into four distinct sections (A–D) for the purpose of analysis. All of the subsets cover a full five years. Our Data in the World and the World Bank (WB) both have datasets that we analyzed using panel data. Inspired by eco-economic separation and a byproduct of techno-nationalism, accessibility is a determining element in the metrics' selection. The primary objective of this research is to assess the impact of CLS-A on the decoupling of CO_2 emissions in a statistically valid sample of Turkey. This increase is mostly attributable to the country's heavy use of coal to meet its growing energy demands. We investigate how these nations' actions affect decoupling on a global scale [39]. Based on their ecological situation and agricultural progress, we have ranked the nations [40]. Because of the association between reliance on these resources and volatility, it is widely believed that the price environment for commodities derived from them threatens the nation's purpose of sustainable economic growth. We determine that changes in the response variables are due to a decoupling estimate ranging from +1 to -2, which is a surrogate for measuring the effectiveness of agricultural initiatives to reduce CO_2 emissions harm [41]. The decoupling concept is further explained by looking at the performance of the portfolio. Conversely, if the decoupling analysis turns out to be unfavorable, we use techno-nationalism to rein in the CO_2 emissions decoupling effect and come up with workable solutions for the agricultural sector's transformation. The decoupling method was used to conduct a weighted calculation [42]. It demonstrates that as environmental conditions change by one percentage point, field burning, managed soil, urea application, manure management, enteric fermentation, and rice cultivation all change proportionately. The weighted values with a flexible range became readily apparent when distinct attitudes were uncoupled [43].

3.3. Portfolio Returns

The CLS-A indicator's estimated weighted return value in the portfolio reveals how much of an impact the better decoupling approach has on the return. Two outcomes could occur. The first is the disparity between the variable's Turkish investment breadths. The indicators over the next eight years are a further factor to think about. The weighted risk and possible return are determined using the consequences of the environment, with the shared aid of decoupling. There are three steps to the calculation process [44]. The average return and risk value variable is one technical evaluation statistic that differentiates the four countries' economies (A–D) [45]. The average return is calculated in this research using total risk and average beta decoupling. That is consistent with the CO_2 emissions standard deviation for the past eight years. Secondly, we determined the portfolio weight by using the relative relevance of the three performance measures (agricultural) to assign a value to each. We allot a certain percentage of the portfolio to each variable according to criteria like CO_2 emissions impact and economic development. The third phase relies on the concept of weighted rewards and risk [46]. This portfolio's enormous potential gains and losses are shown by the big swings. Additionally, the disparity between risk and weighted return is brought to light. To reduce CO_2 emissions risks to manageable levels, it is necessary to evaluate the viewpoints of the most risk-tolerant individuals, which can be achieved through changes in technology changes in CLS-A and strategic policies [47].With this data, we were able to compute the average return and total risk, which we then adjusted using weighted percent. For the most part, previous studies have used a stochastic approach to explaining the portfolio selection problem, treating asset returns as random variables, and have assumed that the statistical feature of agricultural stock returns will remain constant [48]. Although decoupling can be helpful, modeling

portfolio selection under weighted return is problematic owing to the dynamic environment in different regions. In addition, multiply the total risk, average return, and estimated weighted value of each category by their corresponding values [49]. We calculate the weighted return value by averaging the return value. The risk value is an inevitable starting point for the weighted risk value. In this case, we estimate the total risk and average return as we approach the beta decoupling levels. It was able to accomplish this by taking a more solid and sincere approach to the percentage change in agricultural technology [48, 50].

Pollutants released into the atmosphere by fertilizer applications, manure management, and other agricultural inputs are all part of the agricultural inputs that contribute to the degradation of naturally occurring soils. Industrial waste products, such as crop stubble and straw, are associated with emissions from field burning [51]. One major contributor to GHG emissions is the widespread use of urea fertilizer in CLS-A production. Among the numerous agricultural sources, such as manure management, field burning, urea application, enteric fermentation, and agricultural soils, among many others, urea—a nitrogen-based fertilizer—can release nitrous oxide, a potent CLS-A. All emissions from agricultural sources add up to total CO_2 emissions. [52–55]. The findings unequivocally demonstrate that emissions in each of the four categories have demonstrated a progressively increasing trend over the course of the years. The emissions from managed soils have registered the most significant increase, which is most likely attributable to the increased application of fertilizers and manure in order to improve crop yields [56]. In addition, the data clearly demonstrate that CLS-A plays a crucial part in contributing to Turkey's overall CO_2 emissions profile. In the year 2020, the agriculture sector was responsible for around twenty percent of the total emissions that were produced by the nation. The information presented in Appendix A provides a rundown of the CO_2 emissions that will be produced by Turkish CLS-A in the year 2020 [57]. One of the most significant contributors to carbon dioxide emissions within Turkish CLS-A is enteric fermentation, which accounts for approximately forty percent of the overall emissions [58]. The second-biggest source of carbon dioxide emissions from Turkish CLS-A, after manure management, accounts for about 25% of the overall emissions [59]. Animal manure has the potential to release two powerful greenhouse gases—methane and nitrous oxide—during the storage, processing, and application phases of manure management. Furthermore, for Turkey's CO_2 emissions, CLS-A soils account for more than 15% of the overall total. Agricultural soil organic matter cultivation and nitrogen fertilizer use both contribute to the formation of nitrous oxide, the gas responsible for these emissions [60].

4. Results

We used data for Turkey nations from 1992 to 2023 and the portfolio return concept to look at how resurgence with repeated and single aeration and continuous flooding affected high- and low-risk zones. The portfolio return is computed on the basis of the decoupling technique. Specifically, it is demonstrated that a 1 percent change in multiple and single aeration and continuous flooding leads to a 1 percent change in CLS-A. In Fig. 1, we can observe the pattern of wheat yields in Turkey throughout the four categories (A-D). In this portfolio, returns for all CLS-A figures show the formatting condition with three marks, like a checkmark, an exclamation mark, and a heavy cross mark. The checkmark shows the less than 70% decoupling attitude when the result is more than 70%, so it records an exclamation mark, and when the result is more than 30%, it indicates heavy cross. Furthermore, marks show that field burning, managed soil and urban development in terms of CLS-A sectors [61, 62]. From 2.4 metric tons/ha in 2013–14 to 2.5 metric tons/ha in 2022–23, it showed that wheat yields have been rising consistently. We expect wheat yields to rise even further in 2023–24, reaching 2.7 tons per hectare. Fig. 1 reveals that Turkey's wheat area remained virtually unchanged at

7,020,000 hectares (ha) between 2015/16 and 2019/20, compared to 7,860,000 ha in 2016/17. However, the wheat harvest has been more unpredictable, fluctuating between 15,000 metric tons in 2014–15 and 21,000 metric tons in 2017–18. The projected wheat harvest for 2023–24 is 19,500 million metric tons.

4.1 High-Risk Zone of CLS-A

According to Fig. 1, the portfolio CLS-A need is assessed for multiple and single aeration and continuously flooded separately in high- and low-risk zones. Firstly, we analyzed nations with a high-risk factor. In our assessment, along with each country's efforts to combat climate change, we discovered that new technical regulations impact the CLS-A separation characterized by three separate structural marks.

			Managed Soil	Field burining	Urea application	Enteric fermentation	Manure management	Rice cultivation
Continously Flooded	Av.return	Group A	✔ 0.164	✘ -0.511	! -0.190	✔ 0.130	! -0.090	✘ -0.105
		Group B	✘ -0.197	✔ -0.047	✔ 0.052	✘ -0.007	✘ -0.526	✘ -0.215
		Group C	✔ 0.202	✘ -0.394	✘ -0.396	✔ 0.099	✔ 0.204	✔ 0.154
		Group D	✘ -0.135	✔ -0.106	✔ -0.073	✘ -0.004	✔ 0.096	✔ 0.042
	Total Risk	Group A	✔ 0.064	✔ -0.023	✘ -0.047	✔ 0.455	✔ 0.237	✘ -0.035
		Group B	✔ 0.011	✔ 0.104	✔ 0.118	! 0.067	✘ 0.015	✔ 0.248
		Group C	✔ -0.012	✘ -0.358	! 0.025	✔ 0.231	! 0.118	✘ 0.000
		Group D	✘ -0.178	! -0.058	! 0.018	✘ -0.341	! 0.094	✘ -0.063
Single Aeration	Av.return	Group A	✘ -0.924	! 0.624	! 0.382	! 0.214	✔ 0.805	! 0.337
		Group B	✔ 0.526	✘ 0.192	✘ 0.105	✘ 0.100	✔ 0.475	✘ 0.240
		Group C	✔ 0.292	✔ 0.993	✔ 0.856	✔ 0.386	✘ -0.607	✘ 0.239
		Group D	✘ -0.491	✘ 0.357	✘ 0.130	✘ 0.034	✔ 0.400	✔ 0.442
	Total Risk	Group A	! 0.045	! 0.055	✘ -0.119	✔ -0.105	✔ 0.066	✔ 0.090
		Group B	! -0.122	✘ 0.000	✘ -0.127	✔ -0.157	✔ -0.003	! -0.171
		Group C	✔ 0.373	✘ 0.047	✔ 0.074	✔ -0.125	✔ -0.014	✔ -0.030
		Group D	✘ -0.553	✔ 0.147	✘ -0.177	✘ -0.318	✘ -0.178	✘ -0.360
Multiple Aeration	Av.return	Group A	✔ 0.331	✔ -0.273	✔ 0.129	✔ 0.139	✔ 0.556	✔ 0.401
		Group B	✔ -0.045	✔ 0.055	✔ -0.119	✔ -0.105	✔ -0.066	✔ -0.090
		Group C	✘ 0.292	✔ 0.993	✔ 0.856	✘ -0.386	✔ 0.607	✘ 0.239
		Group D	✔ 0.491	✘ 0.357	✘ 0.130	✔ 0.034	✘ -0.400	✔ 0.442
	Total Risk	Group A	✘ -0.122	✔ 0.000	✘ -0.127	✘ -0.157	✔ 0.003	✘ -0.171
		Group B	✔ 0.373	✘ -0.047	✔ 0.074	✔ -0.125	✘ -0.014	✔ -0.030
		Group C	✔ 1.249	✔ 0.567	✘ 0.108	✘ -0.570	✔ 0.456	✔ 0.244
		Group D	✘ 0.248	✘ 0.297	✔ 0.238	✔ 0.930	✘ 0.423	✘ 0.194

Fig 1: Portfolio return of multiple and single aeration and continuously flooded in high risk zone

Similarly, in Fig. 1, portfolio-weighted results of more than 70% are shown by the exclamation mark in high- and low-risk countries, respectively. In terms of total risk and average return in weighted portfolio return, the largest decoupling attitude is reported in Turkey (0.248% and 1.249%) in field burning (0.567% and 0.297%) in groups D and C, respectively. Similarly, rice cultivation has the strongest attitudes toward (0.194% and 0.244%) and enteric fermentation in single aeration (-0.318% and -125%) in group C. In group D, the urea application of -0.341% with a 0.231. The managed soil decoupling attitudes demonstrate -0.553% and 0.373% in single aeration in groups C and D, respectively [42]. Turkey has the most positive attitude about urea application, with 0.018% and 0.025% in groups D. Hence, CLS-A from managed soils includes emissions from cultivating and managing agricultural areas, which includes fertilizer applications, manure management, and other agricultural inputs. The combustion of crop leftovers, such as straw and stubble, is associated with field burning emissions [63]. Use of urea fertilizer in agricultural cultivation is the source of urea application emissions. The nitrogen fertilizer urea has the ability to release nitrous oxide, a powerful CO_2 emission. Emissions from all agricultural activities, such as manure management, field burning, urea application, enteric fermentation, and agricultural soils, make up CLS-A's total emissions.

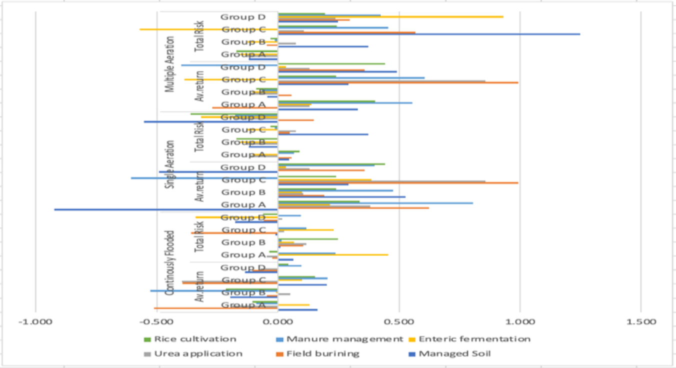

Fig 2: Portfolio return of multiple and single aeration and continuously flooded in high risk

Fig. 2 demonstrates that growing rice releases a lot of carbon dioxide. The predicted 2020 CO_2 equivalent emissions from rice agriculture were 261.53 million tons, as shown in the table. All told, this accounts for around 1.4% of the world's CO_2 emissions. Compared to intermittently flooded rice agriculture, continuously flooded rice cultivation produces greater carbon dioxide emissions [64]. This is due to the fact that, in anaerobic conditions, methane is more readily produced in continually flooded rice paddies, making them a powerful source of carbon dioxide emissions [65]. Over the years, emissions from rice farming have been on the rise. There has been a roughly 50% rise in emissions from rice cultivation since 1990, as seen in the table. Several factors have contributed to this, such as the rise in fertilizer use, the extension of rice agriculture into previously uncultivated areas (such as wetlands), and the overall increase in rice production [29].

4.2 Low-Risk Zone of CLS-A

Fig. 3 illustrates the portfolio return of low-risk countries weighted by risk and return with varying ratios in the CLS-A sector. Initially, the approximated results are analyzed for both single and multiple aeration and continuous flooding in the low-risk zone.

			Managed Soil	Field burining	Urea application	Enteric fermentation	Manure management	Rice cultivation
Continously Flooded	Av.return	Group A	0.038	-0.058	-0.042	0.694	-0.280	-0.111
		Group B	-0.203	0.277	0.097	-0.045	-0.036	-0.043
		Group C	0.126	0.045	0.023	-0.479	-0.574	-0.206
		Group D	-0.467	0.108	0.478	-0.663	0.264	0.255
	Total Risk	Group A	0.278	0.414	0.141	1.832	0.424	0.178
		Group B	0.708	0.742	0.168	0.052	0.217	0.175
		Group C	0.832	0.326	0.200	1.356	0.780	0.254
		Group D	0.834	0.891	1.079	0.668	0.525	0.434
Single Aeration	Av.return	Group A	0.187	0.023	0.141	-0.200	-0.353	-0.226
		Group B	-0.401	0.504	0.123	0.004	-0.347	-0.319
		Group C	-0.136	-0.085	-0.053	-0.317	-0.616	-0.114
		Group D	-0.021	0.125	-0.011	0.427	-0.337	-0.269
	Total Risk	Group A	0.402	0.448	0.566	0.813	0.355	0.261
		Group B	1.043	1.344	0.288	0.081	0.771	0.743
		Group C	0.159	0.107	0.054	0.774	0.800	0.595
		Group D	0.589	1.390	0.405	1.540	0.415	0.392
Multiple Aeration	Av.return	Group A	-0.026	-0.959	0.110	0.237	1.006	0.178
		Group B	-0.047	-0.028	-0.028	0.622	-0.012	0.486
		Group C	-0.186	-0.167	-0.086	-0.085	0.064	-0.217
		Group D	-0.305	-0.004	0.162	-0.252	-0.810	0.039
	Total Risk	Group A	0.231	1.169	0.366	0.680	1.826	0.614
		Group B	0.139	0.275	0.076	1.154	0.318	0.899
		Group C	0.791	0.490	0.245	0.566	0.216	0.287
		Group D	0.751	0.693	0.230	0.393	1.079	0.225

Fig. 3: Portfolio return of multiple and single aeration and continuous flooding in a low-risk zone.

The maximum weighted risk in group D is estimated to be field burning (1.390), multiple aeration (0.693), and manure management (1.079) in China, a high-risk country [66]. From about 70% in 2000 to about 75% in 2019, the percentage of CLS-A in Turkey's water abstractions has been steadily rising. This data points to the fact that CLS-A is using more and more water in Turkey. Additionally, Turkey's irrigated land share has been steadily rising, from about 20% in 2000 to about 25% in 2019. This indicates that irrigation is becoming more important for Turkish farmers when it comes to crop production [67]. Throughout the years, CLS-A's contribution to Turkey's CO_2 emissions has remained around 20%. Therefore, CLS-A probably isn't a big deal when it comes to Turkey's CO_2 emissions. From about 10% in 2000 to about 8% in 2019, CLS-A's proportion of Turkey's overall energy consumption has been steadily declining. This data points to an increase in CLS-A's energy efficiency in Turkey. It has been more common to take phosphorus from the soil in Turkey than to add it, resulting in a negative phosphorus balance since the year 2000.

It appears that the soils of Turkey are losing their phosphorus content. Since the year 2000, Turkey has also had a negative nitrogen balance, which means that the country is removing more nitrogen from the soil than it is adding. This indicates that the nitrogen levels in Turkey's soils are declining. These conclusions point to water scarcity, soil degradation, and climate change as some of the difficulties that CLS-A in Turkey is encountering. The Turkish government must take action to address these concerns if CLS-A is to continue operating in the future. Consequently, the utilization of rice and urea has resulted in a 0.392 and 0.405 reduction, respectively, in agricultural productivity within group D. In the low-risk zone, group C has the highest weighted risk at 0.774, which is 1.98 times higher than group D's 1.540. Since 1990, when it was 37.9 million hectares, the irrigated area for growing rice has grown significantly, reaching 125.4 million hectares in 2020 [68]. From 1990 to 2020, the estimated emissions from rice agriculture have climbed continuously, reaching 261.530 million tons of CO_2 emissions equivalent. Figure 4 demonstrates that rice cultivation has the greatest attitude in single aeration, which is really important because rice is both a staple crop for many people's diets and a big agricultural crop in the country. From a respectable 7.6 metric tons per hectare in 2013–14 to an impressive 8.30 metric tons per hectare in 2023 to 2024, Turkish rice yields have been steadily increasing over the previous decade. There are three major reasons for this impressive expansion. As a first point, the advancements in technology [69]. Modern seed types, precision CLS-A methods, and integrated pest control concepts are just a few examples of how Turkish farmers are fully embracing cutting-edge agricultural technology [70].

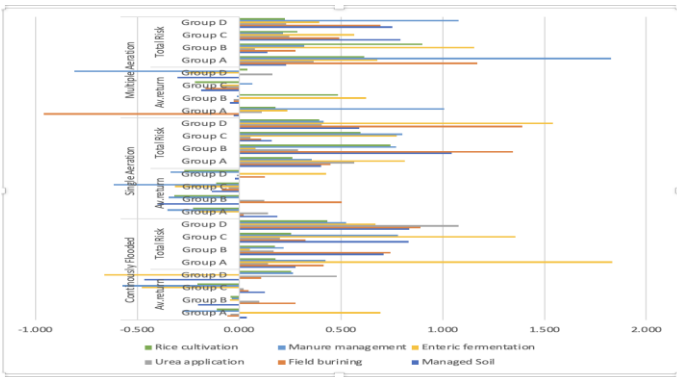

Fig 4: Portfolio return of multiple and single aeration and continuously flooded

There is no denying that these technological advancements have been the foundation of the increased rice harvests. Secondly, backing from the government: The Turkish government has done an admirable job of becoming a reliable backer of rice farming, offering

crucial assistance in the form of subsidies, loans, and outreach programs. Thanks to this strategic backing, the nation's rice farming has become much more efficient and productive [71]. Thirdly, climatic provisions that are beneficial; in recent years, Turkey's climate has been great for rice growing. The positive dynamics of CO_2 emissions have created an enabling environment for the enhancement of rice yields. Figure 5 illustrates that under group D, the Turkish economy and agricultural landscape have benefited greatly from the increasing rice yields achieved through enteric fermentation, manure management, and rice planting. Increased yields allow farmers to get more rice out of their land, which improves their financial situation. Additionally, Turkish rice gains a competitive edge on the world stage due to a dramatic decrease in production overheads brought about by an increase in yields.

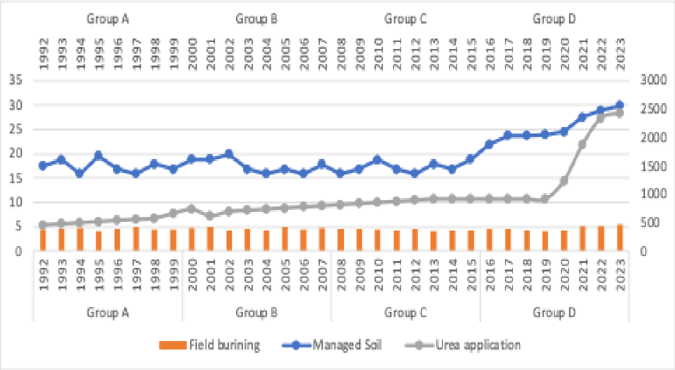

Fig. 5: Managed Soil, Field burning and Urea application

Like the rest of the world, Turkey is facing the daunting prospect of climate change as it pertains to agricultural output. Our farming community may have to adjust to changing weather conditions and look into new agricultural technology as a safety net if we want to survive this enormous challenge and keep or increase our rice harvests for the future. Agricultural activities such as liming, field burning, and rice planting also contribute to emissions. Additionally, the table highlights how the agricultural sector plays a major part in Turkey's CO_2 emissions profile, accounting for around 20.21 percentage of the overall emissions. From the table, we may deduce several important things.

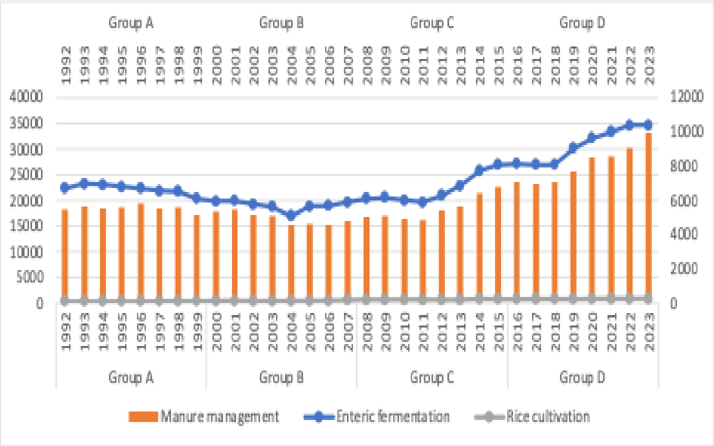

Fig. 6: Enteric fermentation, manure management and rice cultivation

The critical need to decrease emissions the data emphasizes the critical importance of the Turkish agricultural industry reducing its CO_2 emissions immediately [72, 73]. In order to

lower Turkey's total carbon footprint and achieve its climate change mitigation goals, this step is crucial. Measures to decrease emissions: the agricultural sector in Turkey can decrease its CO_2 emissions by implementing a number of proven solutions. Improving manure management, reducing nitrogen fertilizer use, and switching to more environmentally friendly farming methods are all part of these steps [51].

From 2014 to 2023, the line graph in figure 6 displays the maize yields in Turkey. Crop yields for maize have been trending upwards for a while now. responding to climate change and securing a future food supply, which ranks somewhat higher in group D when it comes to growing rice. Rising rice harvests in Turkey are an encouraging trend [51]. Improving efficiency and output while capitalizing on favorable weather conditions is a hallmark of the remarkable transition that the country's rice farmers are presently experiencing. But we must not lose sight of the fact that increasing rice yields may not be sustainable forever [52].

5. Discussion

We are observing that the frequency of droughts, floods, and heat waves is increasing, and they are inflicting greater damage with each occurrence. It is presenting producers with additional obstacles in the areas of animal husbandry and plantation. The data in the image is not reliable for scientific interpretation as a result of the modifications. It is imperative to possess the most recent data on CLS-A in order to make informed decisions regarding agriculture [74]. Farmers have consequently restricted alternatives for cultivating produce and livestock. The information in the image is no longer applicable for scientific research as a result of these modifications. Accurate and up-to-date information regarding CLS-A should be the foundation for any modifications to agriculture policy and practice [75]. Turkey is a prominent producer of wheat, and wheat is a staple in the cuisines of numerous individuals worldwide [76]. Consequently, producers are applying an increased quantity of fertilizers to the soil [77].

Numerous Turkish farmers are currently employing a variety of techniques, including crop rotation, to not only maintain the soil but also to prevent the excessive use of water. A further contributing factor to Turkey's record-breaking wheat harvest was the favorable weather [78]. Turkey has recently encountered mild winters and an abundance of rainfall in the course of the growing season. That is why growing wheat yielded such excellent results. [79]. An increase in wheat harvests in when looking at the worldwide need for food, Turkey is a promising sign, and the country itself. Turkey's dependence on imports will diminish as its wheat production increases. The conflict in Ukraine is a contributing factor to the global food shortage; therefore, it is unnecessary to disregard this issue [80]. The Turkish government should guarantee that producers continue to receive support payments to assist them in enhancing their wheat harvest [81].

6. Conclusion

Turkey can reduce maize harvest risks by taking the following steps: The CLS-A sector can improve collaboration among many agencies, strengthen important ministry of food, CLS-A, and livestock components, and make other positive actions to prevent climate change. We intend to disseminate information regarding the potential effects of climate change on CLS-A and the potential measures that can be implemented to mitigate the risks. Every one of these goals has its own set of essential steps that will lead us to success. First and foremost, we aim to increase interagency cooperation and build capability. Second, strengthening drought management capacity: The objective stresses the need to strengthen the capacity of the committees and agencies responsible for agricultural drought management. As a third point, there are training and research programs to deal with the effects of local and regional climate

change. It is strongly recommended that the ministries of food, CLS-A, and cattle, along with their affiliated institutes, establish a distinct department within their respective provincial organizations to address matters pertaining to climate change.

To adapt to the CLS-A sector's impacts of climate change, it is crucial to coordinate Turkey's policies, strategies, and legislative framework, as stated in the objective. The goal of these programs is to promote sustainable farming practices while simultaneously ensuring the safety and security of our food supply. Incorporating climate change considerations into existing policies and initiatives is crucial for effectively addressing these issues. Second, highlights the importance of conducting and expanding scientific studies and research in order to understand the impacts of climate change on CLS-A and to facilitate adaptation to these changes. The paper focuses on the following main points. To begin, economic development goals are a common impetus for agricultural research and development projects, which have traditionally prioritized increasing production. This has led to less focus on how to adjust to a changing climate. Second, CLS-A's shift to climate revolution adaptation: The goal emphasizes the need to redirect research efforts towards adapting to climate change, especially those that focus on soil and water resource protection. Developing irrigation systems, adjusting production patterns, increasing crop diversity, and disaster management strategies are all necessary to address the impacts of climate change on CLS-A on a national scale, especially in relation to drought. The third component of the plan is socio-economic research. This study will focus on CLS-A, food, the environment, and rural development on a national scale. It will take climate change into account. The findings of this study will pave the way for innovative policies that encourage the expansion of the country's agricultural sector in response to climate change, and they will also assist in securing the livelihoods of specific communities, such as women farmers. Fourth, long-term food security: The plan stresses building a food security system around data uncovered by R&D initiatives that account for the impacts of climate change. Aiming to meet customer expectations while responding to changing factors affecting CO_2 emissions, this system is always evolving. As for the fifth objective, building a database and information system, it highlights the importance of developing such a system at a regional or basin-scale, or even nationally. This approach can be adjusted on a regular basis to account for changing climatic impacts; it will help evaluate and monitor how climate change is affecting farming.

Appendix A:

		Managed Soil		Field burining		Urea application		Agriculture total		Enteric fermentation		Manure management		Rice cultivation		Agriculture total	
	Years	(Kt CO2 eq.)	(%)	(Kt CO2 eq.)	(%)	(Kt CO2 eq.)	(%)	(Kt CO2 eq.)	(%)	(kt COz eq.)	(%)	(kt COz eq.)	(%)	(kt COz eq.)	(%)	(kt COz eq.)	(%)
Group A	1992	17.433	40.2	373	0.7	456	1.0	46541	100	22397	48.6	5436	11.8	100	0.2	46054	100
	1993	18.764	39.6	391	0.8	476	1.3	47654	100	23221	49.5	5657	12.1	100	0.2	46928	100
	1994	15.876	39.0	411	0.7	496	1.0	44987	100	23025	49.6	5533	11.8	100	0.2	46979	100
	1995	19.543	38.4	353	0.8	516	1.0	44651	100	22636	47.7	5597	11.8	101	0.2	47407	100
	1996	16.876	37.8	393	0.7	536	1.2	44651	100	22339	49.7	5793	12.9	100	0.2	44926	100
	1997	15.875	37.2	425	0.8	556	1.1	45761	100	21815	49.5	5523	12.5	113	0.3	44080	100
	1998	17.876	36.6	372	0.7	576	1.0	45871	100	21792	48.7	5570	12.4	126	0.3	44757	100
	1999	16.875	36.0	372	0.8	658	1.5	43988	100	20313	47.8	5166	12.2	124	0.3	42505	100
Group B	2000	18.876	35.4	408	0.8	733	1.7	44312	100	19890	45.5	5348	12.2	135	0.3	43720	100
	2001	18.888	34.8	415	0.7	617	1.5	43987	100	19963	45.1	5448	12.3	147	0.3	44276	100
	2002	19.876	37.9	357	0.8	696	1.3	47611	100	19234	45.4	5142	12.1	128	0.3	42332	100
	2003	16.876	40.2	395	0.7	715	1.4	42876	100	18714	46.9	5096	12.8	132	0.3	39894	100
	2004	15.875	39.6	357	0.7	735	1.4	48761	100	16975	45.1	4540	12.1	135	0.4	37608	100
	2005	16.876	40.2	416	0.8	755	1.5	42987	100	18874	46.5	4596	11.3	143	0.4	40558	100
	2006	15.875	39.0	380	0.7	775	1.4	42998	100	18969	45.9	4590	11.1	156	0.4	41298	100
	2007	17.777	38.4	412	0.8	795	1.3	43123	100	19680	46.4	4781	11.3	183	0.4	42439	100
Group C	2008	15.875	39.0	398	0.7	814	1.3	43211	100	20352	46.4	5027	11.5	212	0.5	43900	100
	2009	16.876	38.4	385	0.8	834	1.4	41765	100	20575	47.4	5081	11.7	203	0.5	43421	100
	2010	18.762	39.0	375	0.7	854	1.4	42987	100	20084	48.6	4929	11.9	216	0.5	41302	100
	2011	16.876	38.4	362	0.5	874	1.5	44877	100	19606	46.6	4863	11.6	208	0.5	42032	100
	2012	15.875	37.8	391	0.5	894	1.2	46876	100	20946	47.2	5391	12.1	202	0.5	44409	100
	2013	17.876	37.2	354	0.4	913	1.2	52987	100	22847	48.7	5639	12.7	204	0.4	46901	100
	2014	16.875	36.6	361	0.4	913	1.4	55766	100	25790	49.5	6425	12.2	249	0.5	52662	100
	2015	18.876	36.0	365	0.4	913	1.4	56112	100	26906	48.2	6769	12.1	231	0.4	55858	100
Group D	2016	21.876	35.4	391	0.3	913	1.4	56332	100	27154	48.3	7068	12.6	229	0.4	56219	100
	2017	23.765	34.8	389	0.3	913	2.2	56431	100	26947	48.1	6956	12.4	240	0.4	56133	100
	2018	23.743	37.3	356	0.7	913	2.3	63987	100	26984	45.8	7060	12.6	243	0.4	58894	100
	2019	23.931	35.2	354	0.8	913	1.9	54233	100	30110	47.6	7697	12.2	234	0.4	63262	100
	2020	24.543	35.8	361	0.7	1232	1.9	54322	100	32136	49.2	8508	12.8	252	0.4	65338	100
	2021	27.543	37.4	431	0.7	1872	2.3	63987	100	33368	49.1	8597	12.6	263	0.4	68023	100
	2022	28.871	39.8	432	0.8	2342	2.4	58986	100	34615	47.3	9060	12.4	262	0.4	73155	100
	2023	29.876	41.9	465	0.7	2431	2.6	63876	100	34651	46.2	9932	12.87	272	0.4	73165	100

References

1. Erdoğan, A., Güler, M.G.: Optimization and analysis of a hydrogen supply chain in terms of cost, CO2 emissions, and risk: the case of Turkey. Int. J. Hydrogen Energy. 48, 22752–22765 (2023). https://doi.org/https://doi.org/10.1016/j.ijhydene.2023.04.300
2. Caliskan, O., Sag, N.B., Ersoy, H.K.: Thermodynamic, environmental, and exergoeconomic analysis of multi-ejector expansion transcritical CO2 supermarket refrigeration cycles in different climate regions of Türkiye: Analyse thermodynamique, environnementale et exergoéconomique des cycles de ré. Int. J. Refrig. (2024). https://doi.org/https://doi.org/10.1016/j.ijrefrig.2024.05.006
3. Uzar, U., Eyuboglu, K.: Testing the asymmetric impacts of income inequality, financial development and human development on ecological footprint in Türkiye: A NARDL approach. J. Clean. Prod. 461, 142652 (2024). https://doi.org/https://doi.org/10.1016/j.jclepro.2024.142652
4. Isik, M., Sarica, K., Ari, I.: Driving forces of Turkey's transportation sector CO2 emissions: An LMDI approach. Transp. Policy. 97, 210–219 (2020). https://doi.org/https://doi.org/10.1016/j.tranpol.2020.07.006
5. March, A., Bennett, M., Germishuizen, M., Evans, T., Failler, P.: The status of Blue Economy development in Africa. Mar. Policy. 165, 106205 (2024). https://doi.org/https://doi.org/10.1016/j.marpol.2024.106205
6. Köprücü, Y., Acaroğlu, H.: How cereal yield is influenced by eco-environmental factors? ARDL and spectral causality analysis for Turkey. Clean. Environ. Syst. 10, 100128 (2023). https://doi.org/https://doi.org/10.1016/j.cesys.2023.100128
7. Abdallah, I., Udemba, E.N.B.T.-R.M. in S.S.: Industrial and agricultural progression amid pollution: A time series study of Turkey. Presented at the (2023)
8. Knorr, S., Weisener, C.G., Phillips, L.A.: Agricultural land management alters the biogeochemical cycling capacity of aquatic and sediment environments. Agric. Ecosyst. Environ. 357, 108661 (2023). https://doi.org/https://doi.org/10.1016/j.agee.2023.108661
9. Naimoglu, M., Akal, M.: The relationship between energy technology, energy efficiency, renewable energy, and the environment in Türkiye. J. Clean. Prod. 418, 138144 (2023). https://doi.org/https://doi.org/10.1016/j.jclepro.2023.138144
10. Deng, P., Tan, X., Bai, Y., Li, H.: Forecasting chaotic behavior of the drill bit in real-time using a DNN model integrated with rock surface morphology. Mech. Syst. Signal Process. 221, 111710 (2024). https://doi.org/https://doi.org/10.1016/j.ymssp.2024.111710
11. Ağbulut, Ü.: Forecasting of transportation-related energy demand and CO2 emissions in Turkey with different machine learning algorithms. Sustain. Prod. Consum. 29, 141–157 (2022). https://doi.org/https://doi.org/10.1016/j.spc.2021.10.001
12. Isik, M., Ari, I., Sarica, K.: Challenges in the CO2 emissions of the Turkish power sector: Evidence from a two-level decomposition approach. Util. Policy. 70, 101227 (2021). https://doi.org/https://doi.org/10.1016/j.jup.2021.101227
13. Goldenberg, M.G., Burian, A., Seppelt, R., Santibañez Ossa, F.A., Bagnato, C.E., Satorre, E.H., Martini, G.D., Garibaldi, L.A.: Effects of natural habitat composition and configuration, environment and agricultural input on soybean and maize yields in Argentina. Agric. Ecosyst. Environ. 339, 108133 (2022). https://doi.org/https://doi.org/10.1016/j.agee.2022.108133
14. Yurtkuran, S.: The effect of agriculture, renewable energy production, and globalization on CO2 emissions in Turkey: A bootstrap ARDL approach. Renew.

Energy. 171, 1236–1245 (2021). https://doi.org/https://doi.org/10.1016/j.renene.2021.03.009
15. Onenli, O., Ercan, H., Acikgoz, S., Kat, B.: Emission reduction pathways for satisfying Türkiye’s future electricity demand: A cross-country dynamic panel analysis with linear programming scenarios. Clean. Energy Syst. 6, 100085 (2023). https://doi.org/https://doi.org/10.1016/j.cles.2023.100085
16. Karki, E., Sharma, A., Timsina, P., Chaudhary, A., Sharma, R., Brown, B.: Strategies to overcome stagnation in agricultural adoption despite awareness and interest: a case study of conservation agriculture in South Asia. Renew. Agric. Food Syst. 39, e14 (2024). https://doi.org/DOI: 10.1017/S1742170524000073
17. Alatawneh, A., Ghunaim, D.: Towards vehicle electrification: A mathematical prediction of battery electric vehicle ownership growth, the case of Turkey. Green Energy Intell. Transp. 100166 (2024). https://doi.org/https://doi.org/10.1016/j.geits.2024.100166
18. Temizceri, F.T., Kara, S.S.: Towards sustainable logistics in Turkey: A bi-objective approach to green intermodal freight transportation enhanced by machine learning. Res. Transp. Bus. Manag. 55, 101145 (2024). https://doi.org/https://doi.org/10.1016/j.rtbm.2024.101145
19. Adebayo, T.S., Agyekum, E.B., Kamel, S., Zawbaa, H.M., Altuntaş, M.: Drivers of environmental degradation in Turkey: Designing an SDG framework through advanced quantile approaches. Energy Reports. 8, 2008–2021 (2022). https://doi.org/https://doi.org/10.1016/j.egyr.2022.01.020
20. Guven, D., Kayalica, M.O.: Analysing the determinants of the Turkish household electricity consumption using gradient boosting regression tree. Energy Sustain. Dev. 77, 101312 (2023). https://doi.org/https://doi.org/10.1016/j.esd.2023.101312
21. Türker, Y.Ö., Aydin, A.: How ready is the Turkish Legislation for the green deal? Energy Clim. Chang. 3, 100084 (2022). https://doi.org/https://doi.org/10.1016/j.egycc.2022.100084
22. Bakay, M.S., Ağbulut, Ü.: Electricity production based forecasting of greenhouse gas emissions in Turkey with deep learning, support vector machine and artificial neural network algorithms. J. Clean. Prod. 285, 125324 (2021). https://doi.org/https://doi.org/10.1016/j.jclepro.2020.125324
23. As, M., Bilir, T.: Enhancing energy efficiency and cost-effectiveness while reducing CO2 emissions in a hospital building. J. Build. Eng. 78, 107792 (2023). https://doi.org/https://doi.org/10.1016/j.jobe.2023.107792
24. Acar, S., Kat, B., Rogner, M., Saygin, D., Taranto, Y., Yeldan, A.E.: Transforming Türkiye’s power system: An assessment of economic, social, and external impacts of an energy transition by 2030. Clean. Energy Syst. 4, 100064 (2023). https://doi.org/https://doi.org/10.1016/j.cles.2023.100064
25. Gökçek, M., Paltrinieri, N., Liu, Y., Badia, E., Dokuz, A.Ş., Erdoğmuş, A., Urhan, B.B., Yoldaş, Ö.: Optimum sizing of hybrid renewable power systems for on-site hydrogen refuelling stations: Case studies from Türkiye and Spain. Int. J. Hydrogen Energy. 59, 715–729 (2024). https://doi.org/https://doi.org/10.1016/j.ijhydene.2024.02.068
26. Karakurt, I., Aydin, G.: Development of regression models to forecast the CO2 emissions from fossil fuels in the BRICS and MINT countries. Energy. 263, 125650 (2023). https://doi.org/https://doi.org/10.1016/j.energy.2022.125650
27. Cekinir, S., Ozgener, O., Ozgener, L.: Türkiye’s energy projection for 2050. Renew. Energy Focus. 43, 93–116 (2022). https://doi.org/https://doi.org/10.1016/j.ref.2022.09.003

28. Eslami, S., Noorollahi, Y., Marzband, M., Anvari-Moghaddam, A.: District heating planning with focus on solar energy and heat pump using GIS and the supervised learning method: Case study of Gaziantep, Turkey. Energy Convers. Manag. 269, 116131 (2022). https://doi.org/https://doi.org/10.1016/j.enconman.2022.116131
29. Destek, M.A.: Deindustrialization, reindustrialization and environmental degradation: Evidence from ecological footprint of Turkey. J. Clean. Prod. 296, 126612 (2021). https://doi.org/https://doi.org/10.1016/j.jclepro.2021.126612
30. Yilanci, V., Kilci, E.N.: A study on the macroeconomic and financial determinants of telecommunication infrastructure: Evidence from Turkiye. Technol. Soc. 77, 102573 (2024). https://doi.org/https://doi.org/10.1016/j.techsoc.2024.102573
31. Khan, R.: The impact of a new techno-nationalism era on eco-economic decoupling. Resour. Policy. 82, 103452 (2023). https://doi.org/https://doi.org/10.1016/j.resourpol.2023.103452
32. Khan, R.: Beta decoupling relationship between CO2 emissions by GDP, energy consumption, electricity production, value-added industries, and population in China. PLoS One. 16, 1–22 (2021). https://doi.org/10.1371/journal.pone.0249444
33. Khan, R.: Comparing the effects of agricultural intensification on CO2 emissions and energy consumption in developing and developed countries. Front. Environ. Sci. https://doi.org/10.3389/fenvs.2022.1065634
34. Wang, C., Abbasi, K.R., Irfan, M., Ben-Salha, O., Bandyopadhyay, A.: Navigating sustainability in the US: A comprehensive analysis of green energy, eco-innovation, and economic policy uncertainty on sectoral CO2 emissions. Energy Reports. 11, 5286–5299 (2024). https://doi.org/https://doi.org/10.1016/j.egyr.2024.05.014
35. Khan, R.: The impact of agricultural intensification on carbon dioxide emissions and energy consumption: a comparative study of developing and developed nations.
36. Erdoğan, A., Geçici, E., Güler, M.G.: Design of a future hydrogen supply chain: A multi-objective model for Turkey. Int. J. Hydrogen Energy. 48, 11775–11789 (2023). https://doi.org/https://doi.org/10.1016/j.ijhydene.2022.12.071
37. Khan, S.Z.R.: Performance evaluation of e-commerce firms in China: Using three-stage data envelopment analysis and the Malmquist productivity index. PLoS One. 11, (2021). https://doi.org/10.1371/journal.pone.0255851
38. Rabnawaz Khan, Y.K.: Effects of Energy Consumption on GDP: New Evidence of 24 Countries on Their Natural Resources and Production of Electricity. Ekonomika. 99, 26–49 (2020). https://doi.org/https://doi.org/10.15388/Ekon.2020.1.2
39. Gürel, B., Kurtuluş, K., Yurdakul, S., Karaca Dolgun, G., Akman, R., Önür, M.E., Varol, M., Keçebaş, A., Gürbüz, H.: Combustion of chicken manure and Turkish lignite mixtures in a circulating fluidized bed. Renew. Sustain. Energy Rev. 189, 113960 (2024). https://doi.org/https://doi.org/10.1016/j.rser.2023.113960
40. Kınay, U., Laukkarinen, A., Vinha, J.: Renovation wave of the residential building stock targets for the carbon-neutral: Evaluation by Finland and Türkiye case studies for energy demand. Energy Sustain. Dev. 75, 1–24 (2023). https://doi.org/https://doi.org/10.1016/j.esd.2023.04.014
41. Zambrano-Monserrate, M.A.: Clean energy production index and CO2 emissions in OECD countries. Sci. Total Environ. 907, 167852 (2024). https://doi.org/https://doi.org/10.1016/j.scitotenv.2023.167852
42. Atmaca, A., Atmaca, N.: Carbon footprint assessment of residential buildings, a review and a case study in Turkey. J. Clean. Prod. 340, 130691 (2022). https://doi.org/https://doi.org/10.1016/j.jclepro.2022.130691
43. Jonek-Kowalska, I.: Towards the reduction of CO2 emissions. Paths of pro-ecological transformation of energy mixes in European countries with an above-average share of

coal in energy consumption. Resour. Policy. 77, 102701 (2022). https://doi.org/https://doi.org/10.1016/j.resourpol.2022.102701
44. Najmuddin, Omaid, Zhihui Li, Rabnawaz Khan, and W.Z.: Valuation of Land-Use/Land-Cover-Based Ecosystem Services in Afghanistan—An Assessment of the Past and Future. Land. 11, (2022)
45. Khan, R.: Catch-up growth with alpha and beta decoupling and their relationships between CO2 emissions by GDP, population, energy production, and consumption. Heliyon. e31470 (2024). https://doi.org/https://doi.org/10.1016/j.heliyon.2024.e31470
46. Jebabli, I., Lahiani, A., Mefteh-Wali, S.: Quantile connectedness between CO2 emissions and economic growth in G7 countries. Resour. Policy. 81, 103348 (2023). https://doi.org/https://doi.org/10.1016/j.resourpol.2023.103348
47. Yin, C., Wang, C., Wang, Q., Ge, Y.: Effects of regional freight structure and energy intensity on CO2 emission of transport—a case study in Yangtze River Delta. Int. J. Sustain. Transp. 18, 379–392 (2024). https://doi.org/https://doi.org/10.1080/15568318.2023.2299918
48. Acaroğlu, H., Güllü, M.: Climate change caused by renewable and non-renewable energy consumption and economic growth: A time series ARDL analysis for Turkey. Renew. Energy. 193, 434–447 (2022). https://doi.org/https://doi.org/10.1016/j.renene.2022.04.138
49. Jahanger, A., Hossain, M.R., Awan, A.: Exploring the critical nexus among energy mineral, globalization, and CO2 emissions in NAFTA: What's the forum's response amid asymmetries? Resour. Policy. 90, 104825 (2024). https://doi.org/https://doi.org/10.1016/j.resourpol.2024.104825
50. Schilling, M.A., Esmundo, M.: Technology S-curves in renewable energy alternatives: Analysis and implications for industry and government. Energy Policy. 37, 1767–1781 (2009). https://doi.org/https://doi.org/10.1016/j.enpol.2009.01.004
51. Shahbaz, M., Patel, N., Du, A.M., Ahmad, S.: From black to green: Quantifying the impact of economic growth, resource management, and green technologies on CO2 emissions. J. Environ. Manage. 360, 121091 (2024). https://doi.org/https://doi.org/10.1016/j.jenvman.2024.121091
52. Sufyanullah, K., Ahmad, K.A., Sufyan Ali, M.A.: Does emission of carbon dioxide is impacted by urbanization? An empirical study of urbanization, energy consumption, economic growth and carbon emissions - Using ARDL bound testing approach. Energy Policy. 164, 112908 (2022). https://doi.org/https://doi.org/10.1016/j.enpol.2022.112908
53. ExxonMobil: Advancing climate solutions. (2022)
54. Lioudis, N.: What is the relationship between oil prices and inflation?, http://www.investopedia.com/ask/answers/06/oilpricesinflation.asp
55. Zhang, S., Zhang, X., Du, J., Wang, W., Pi, X.: Multi-target meridians classification based on the topological structure of anti-cancer phytochemicals using deep learning. J. Ethnopharmacol. 319, 117244 (2024). https://doi.org/https://doi.org/10.1016/j.jep.2023.117244
56. Ridoutt, B., Lehnert, S.A., Denman, S., Charmley, E., Kinley, R., Dominik, S.: Potential GHG emission benefits of Asparagopsis taxiformis feed supplement in Australian beef cattle feedlots. J. Clean. Prod. 337, 130499 (2022). https://doi.org/https://doi.org/10.1016/j.jclepro.2022.130499
57. Sheraz, M., Deyi, X., Sinha, A., Mumtaz, M.Z., Fatima, N.: The dynamic nexus among financial development, renewable energy and carbon emissions: Moderating roles of globalization and institutional quality across BRI countries. J. Clean. Prod. 343, 130995 (2022). https://doi.org/https://doi.org/10.1016/j.jclepro.2022.130995

58. Li, D., Guan, X., Tang, T., Zhao, L., Tong, W., Wang, Z.: The clean energy development path and sustainable development of the ecological environment driven by big data for mining projects. J. Environ. Manage. 348, 119426 (2023). https://doi.org/https://doi.org/10.1016/j.jenvman.2023.119426
59. Rehfeldt, M., Fleiter, T., Herbst, A., Eidelloth, S.: Fuel switching as an option for medium-term emission reduction - A model-based analysis of reactions to price signals and regulatory action in German industry. Energy Policy. 147, 111889 (2020). https://doi.org/https://doi.org/10.1016/j.enpol.2020.111889
60. Deng, X., Du, L.: Estimating the environmental efficiency, productivity, and shadow price of carbon dioxide emissions for the Belt and Road Initiative countries. J. Clean. Prod. 277, 123808 (2020). https://doi.org/https://doi.org/10.1016/j.jclepro.2020.123808
61. Shukla, N., Shah, K., Rathore, D., Soni, K., Shah, J., Vora, H., Dave, H.: Androgen receptor: Structure, signaling, function and potential drug discovery biomarker in different breast cancer subtypes. Life Sci. 348, 122697 (2024). https://doi.org/https://doi.org/10.1016/j.lfs.2024.122697
62. Van Poecke, A., Tabari, H., Hellinckx, P.: Unveiling the backbone of the renewable energy forecasting process: Exploring direct and indirect methods and their applications. Energy Reports. 11, 544–557 (2024). https://doi.org/https://doi.org/10.1016/j.egyr.2023.12.031
63. Yang, X., Ramos-Meza, C.S., Shabbir, M.S., Ali, S.A., Jain, V.: The impact of renewable energy consumption, trade openness, CO2 emissions, income inequality, on economic growth. Energy Strateg. Rev. 44, 101003 (2022). https://doi.org/https://doi.org/10.1016/j.esr.2022.101003
64. Perone, G.: The relationship between renewable energy production and CO2 emissions in 27 OECD countries: A panel cointegration and Granger non-causality approach. J. Clean. Prod. 434, 139655 (2024). https://doi.org/https://doi.org/10.1016/j.jclepro.2023.139655
65. Yu, Y., Radulescu, M., Ifelunini, A.I., Ogwu, S.O., Onwe, J.C., Jahanger, A.: Achieving Carbon Neutrality Pledge through Clean Energy Transition: Linking the Role of Green Innovation and Environmental Policy in E7 Countries. Energies. 15, (2022). https://doi.org/10.3390/en15176456
66. Mendonça, A.K. de S., de Andrade Conradi Barni, G., Moro, M.F., Bornia, A.C., Kupek, E., Fernandes, L.: Hierarchical modeling of the 50 largest economies to verify the impact of GDP, population and renewable energy generation in CO2 emissions. Sustain. Prod. Consum. 22, 58–67 (2020). https://doi.org/https://doi.org/10.1016/j.spc.2020.02.001
67. Ozdemir, A.C.: Decomposition and decoupling analysis of carbon dioxide emissions in electricity generation by primary fossil fuels in Turkey. Energy. 273, 127264 (2023). https://doi.org/https://doi.org/10.1016/j.energy.2023.127264
68. Adebayo, T.S., Acheampong, A.O.: Modelling the globalization-CO2 emission nexus in Australia: evidence from quantile-on-quantile approach. Environ. Sci. Pollut. Res. 29, 9867–9882 (2022). https://doi.org/10.1007/s11356-021-16368-y
69. Grodzicki, T., Jankiewicz, M.: The impact of renewable energy and urbanization on CO2 emissions in Europe – Spatio-temporal approach. Environ. Dev. 44, 100755 (2022). https://doi.org/https://doi.org/10.1016/j.envdev.2022.100755
70. Kwilinski, A., Lyulyov, O., Pimonenko, T.: Reducing transport sector CO2 emissions patterns: environmental technologies and renewable energy. J. Open Innov. Technol. Mark. Complex. 100217 (2024). https://doi.org/https://doi.org/10.1016/j.joitmc.2024.100217

71. Peiseler, L., Cabrera Serrenho, A.: How can current German and EU policies be improved to enhance the reduction of CO2 emissions of road transport? Revising policies on electric vehicles informed by stakeholder and technical assessments. Energy Policy. 168, 113124 (2022). https://doi.org/https://doi.org/10.1016/j.enpol.2022.113124
72. Kłosowski, G., Hoła, A., Rymarczyk, T., Mazurek, M., Niderla, K., Rzemieniak, M.: Use of the double-stage LSTM network in electrical tomography for 3D wall moisture imaging. Measurement. 213, 112741 (2023). https://doi.org/https://doi.org/10.1016/j.measurement.2023.112741
73. Llanos, C., Kristjanpoller, W., Michell, K., Minutolo, M.C.: Causal treatment effects in time series: CO2 emissions and energy consumption effect on GDP. Energy. 249, 123625 (2022). https://doi.org/https://doi.org/10.1016/j.energy.2022.123625
74. Meygoonpoury, E., Ghadim, M.K., Ziabakhsh-Ganji, Z.: Internationalization of renewable energy base businesses with a combined approach to networking and collaborative competition. Results Eng. 21, 101726 (2024). https://doi.org/https://doi.org/10.1016/j.rineng.2023.101726
75. Cui, Y., Khan, S.U., Deng, Y., Zhao, M.: Spatiotemporal heterogeneity, convergence and its impact factors: Perspective of carbon emission intensity and carbon emission per capita considering carbon sink effect. Environ. Impact Assess. Rev. 92, 106699 (2022). https://doi.org/https://doi.org/10.1016/j.eiar.2021.106699
76. Arora, T., Reddy, C.S., Sharma, R., Kilaparthi, S.D., Gupta, L.: Greenhouse gas emissions of Delhi, India: A trend analysis of sources and sinks for 2017–2021. Urban Clim. 51, 101634 (2023). https://doi.org/https://doi.org/10.1016/j.uclim.2023.101634
77. Khan, R., Zhuang, W., Najumddin, O., Butt, R.S., Ahmad, I., Al-Faryan, M.A.S.: The impact of agricultural intensification on carbon dioxide emissions and energy consumption: A comparative study of developing and developed nations. Front. Environ. Sci. 10, (2022). https://doi.org/10.3389/fenvs.2022.1036300
78. Ridzuan, N.H.A.M., Marwan, N.F., Khalid, N., Ali, M.H., Tseng, M.-L.: Effects of agriculture, renewable energy, and economic growth on carbon dioxide emissions: Evidence of the environmental Kuznets curve. Resour. Conserv. Recycl. 160, 104879 (2020). https://doi.org/https://doi.org/10.1016/j.resconrec.2020.104879
79. Chang, T., Hsu, C.-M., Chen, S.-T., Wang, M.-C., Wu, C.-F.: Revisiting economic growth and CO2 emissions nexus in Taiwan using a mixed-frequency VAR model. Econ. Anal. Policy. 79, 319–342 (2023). https://doi.org/https://doi.org/10.1016/j.eap.2023.05.022
80. Islam, M.J., Datta, R., Iqbal, A.: Actual rating calculation of the zoom cloud meetings app using user reviews on google play store with sentiment annotation of BERT and hybridization of RNN and LSTM. Expert Syst. Appl. 223, 119919 (2023). https://doi.org/https://doi.org/10.1016/j.eswa.2023.119919
81. Ben Jebli, M., Farhani, S., Guesmi, K.: Renewable energy, CO2 emissions and value added: Empirical evidence from countries with different income levels. Struct. Chang. Econ. Dyn. 53, 402–410 (2020). https://doi.org/https://doi.org/10.1016/j.strueco.2019.12.009
