# Supplementary material for: Exploring climate smart agriculture in Turkey: Enhancing food security and sustainable practices for the reduction of CO₂ emissions
Source: PLoS One. 2026 Mar 31;21(3):e0344924. doi: 10.1371/journal.pone.0344924 (PMC13037976; doi:10.1371/journal.pone.0344924)
Supplement: Attachment — Submitted filename: PONE-D-25-27665_R1_reviewer.pdf [file pone.0344924.s008.pdf]

PLOS ONE

Exploring Climate Smart Agriculture in Turkey: Enhancing Food Security and Sustainable Practices for the Reduction of CO2 Emissions

--Manuscript Draft--

**Manuscript Number:**	PONE-D-25-27665R1
**Article Type:**	Research Article
**Full Title:**	Exploring Climate Smart Agriculture in Turkey: Enhancing Food Security and Sustainable Practices for the Reduction of CO2 Emissions
**Short Title:**	Climate Smart Agriculture
**Corresponding Author:**	Nawaz Khan, PhD<br>Fujian University of Technology<br>Fuzhou , CHINA
**Keywords:**	Climate Smart-Agriculture; portfolio return, environment; CO_2 emissions; Turkey
**Abstract:**	Climate-smart agriculture entails the reduction of CO_2 emissions, the adaptation and modification of technology to enhance resilience to climate change, and the sustainable increase of incomes. The aim of this study is to evaluate the effectiveness of smart agriculture practices in Turkey, which have a significant impact on food security and lower CO_2 emissions. This study employed the decoupling technique to estimate the portfolio returns and analyze their relationship with climate-smart agriculture and CO_2 emissions during the anticipated period of 1992 to 2023. The decoupling technique is responsible for estimating the percentage change in portfolio returns with high and low weighted risk. Furthermore, it has been shown that a percentage change in continuous, single aeration, and multiple aeration practices results in a change in climate-smart agriculture. Food security and sustainable practices contribute to fluctuations in CO_2 emissions levels, and this method also facilitates the forecast of the next five years. The results suggest that the decoupling trend in portfolio return is significantly influenced by factors such as rice cultivation, field rise, and soil management, which also contribute to the highest weighted risk. Additionally, this factor consistently shows the highest weighted importance in determining overall portfolio returns, as it also exhibits the largest marginal effects. Consequently, this investigation substantiates the detrimental influence of these variables on CO_2 emissions. Turkey's sustainable smart agriculture process is essential for the efficient expansion of the economy, as it integrates climate change considerations into current policies and initiatives and reinforces the policy indicator of economic consideration with environmental protection in terms of CO_2 emissions.
**Order of Authors:**	Wang Jie
	Nawaz Khan, PhD
**Response to Reviewers:**	Response of reviewers have been attached.
**Additional Information:**	
**Question**	**Response**
**Financial Disclosure**<br><br>Enter a financial disclosure statement that describes the sources of funding for the work included in this submission. Review the submission guidelines for detailed requirements. View published research articles from *PLOS ONE* for specific examples.	The author(s) received no specific funding for this work.

Powered by Editorial Manager® and ProduXion Manager® from Aries Systems Corporation

This statement is required for submission and **will appear in the published article** if the submission is accepted. Please make sure it is accurate.<br><br>**Funded studies**<br>Enter a statement with the following details:<br>• Initials of the authors who received each award<br>• Grant numbers awarded to each author<br>• The full name of each funder<br>• URL of each funder website<br>• Did the sponsors or funders play any role in the study design, data collection and analysis, decision to publish, or preparation of the manuscript?<br><br>Did you receive funding for this work?	
**Competing Interests**<br><br>Use the instructions below to enter a competing interest statement for this submission. On behalf of all authors, disclose any competing interests that could be perceived to bias this work—acknowledging all financial support and any other relevant financial or non-financial competing interests.<br><br>This statement is required for submission and **will appear in the published article** if the submission is accepted. Please make sure it is accurate and that any funding sources listed in your Funding Information later in the submission form are also declared in your Financial Disclosure statement.<br><br>View published research articles from *PLOS ONE* for specific examples.	The authors have declared that no competing interests exist.

**NO authors have competing interests**<br><br>Enter: *The authors have declared that no competing interests exist.*<br><br>**Authors with competing interests**<br><br>Enter competing interest details beginning with this statement:<br><br>*I have read the journal's policy and the authors of this manuscript have the following competing interests: [insert competing interests here]*<br><br>* typeset	
**Ethics Statement**<br><br>Enter an ethics statement for this submission. This statement is required if the study involved:<br><br>• Human participants<br>• Human specimens or tissue<br>• Vertebrate animals or cephalopods<br>• Vertebrate embryos or tissues<br>• Field research<br><br>Write "N/A" if the submission does not require an ethics statement.<br><br>General guidance is provided below. Consult the submission guidelines for detailed instructions. **Make sure that all information entered here is included in the Methods section of the manuscript.**	N/A

**Format for specific study types**<br><br>**Human Subject Research (involving human participants and/or tissue)**<br>• Give the name of the institutional review board or ethics committee that approved the study<br>• Include the approval number and/or a statement indicating approval of this research<br>• Indicate the form of consent obtained (written/oral) or the reason that consent was not obtained (e.g. the data were analyzed anonymously)<br><br>**Animal Research (involving vertebrate animals, embryos or tissues)**<br>• Provide the name of the Institutional Animal Care and Use Committee (IACUC) or other relevant ethics board that reviewed the study protocol, and indicate whether they approved this research or granted a formal waiver of ethical approval<br>• Include an approval number if one was obtained<br>• If the study involved *non-human primates*, add *additional details* about animal welfare and steps taken to ameliorate suffering<br>• If anesthesia, euthanasia, or any kind of animal sacrifice is part of the study, include briefly which substances and/or methods were applied<br><br>**Field Research**<br><br>Include the following details if this study involves the collection of plant, animal, or other materials from a natural setting:<br>• Field permit number<br>• Name of the institution or relevant body that granted permission	
**Data Availability**<br><br>Authors are required to make all data underlying the findings described fully available, without restriction, and from the time of publication. PLOS allows rare exceptions to address legal and ethical concerns. See the PLOS Data Policy and FAQ for detailed information.	Yes - all data are fully available without restriction

A Data Availability Statement describing where the data can be found is required at submission. Your answers to this question constitute the Data Availability Statement and **will be published in the article**, if accepted.<br><br>**Important:** Stating 'data available on request from the author' is not sufficient. If your data are only available upon request, select 'No' for the first question and explain your exceptional situation in the text box.<br><br>Do the authors confirm that all data underlying the findings described in their manuscript are fully available without restriction?	
**Describe where the data may be found in full sentences. If you are copying our sample text, replace any instances of XXX with the appropriate details.**<br><br>• If the data are **held or will be held in a public repository**, include URLs, accession numbers or DOIs. If this information will only be available after acceptance, indicate this by ticking the box below. For example: *All XXX files are available from the XXX database (accession number(s) XXX, XXX.*).<br>• If the data are all contained **within the manuscript and/or Supporting Information files**, enter the following: *All relevant data are within the manuscript and its Supporting Information files.*<br>• If neither of these applies but you are able to provide **details of access elsewhere**, with or without limitations, please do so. For example:<br><br>*Data cannot be shared publicly because of [XXX]. Data are available from the XXX Institutional Data Access / Ethics Committee (contact via XXX) for researchers who meet the criteria for access to confidential data.*<br><br>*The data underlying the results presented in the study are available from (include the name of the third party*	n/a

and contact information or URL).<br>• This text is appropriate if the data are owned by a third party and authors do not have permission to share the data.<br><br>* typeset	
Additional data availability information:	Tick here if the URLs/accession numbers/DOIs will be available only after acceptance of the manuscript for publication so that we can ensure their inclusion before publication.; Tick here if your circumstances are not covered by the questions above and you need the journal's help to make your data available.

Powered by Editorial Manager® and ProduXion Manager® from Aries Systems Corporation

Manuscript

Click here to access/download;Manuscript;Manuscript Without Trach Changes- 2025-08-17 (A).docx

Exploring Climate Smart Agriculture in Turkey: Enhancing Food Security and Sustainable Practices for the Reduction of CO_2 Emissions

Wang Jie[1] Nawaz Khan[2]*

[1,2]*School of Internet Economics and Business, Fujian University of Technology, Fuzhou City, Fujian Province, China

*Corresponding Author:
Email: 61202008@fjut.edu.cn

Abstract

Climate-smart agriculture entails the reduction of CO_2 emissions, the adaptation and modification of technology to enhance resilience to climate change, and the sustainable increase of incomes. The aim of this study is to evaluate the effectiveness of smart agriculture practices in Turkey, which have a significant impact on food security and lower CO_2 emissions. This study employed the decoupling technique to estimate the portfolio returns and analyze their relationship with climate-smart agriculture and CO_2 emissions during the anticipated period of 1992 to 2023. The decoupling technique is responsible for estimating the percentage change in portfolio returns with high and low weighted risk. Furthermore, it has been shown that a percentage change in continuous, single aeration, and multiple aeration practices results in a change in climate-smart agriculture. Food security and sustainable practices contribute to fluctuations in CO_2 emissions levels, and this method also facilitates the forecast of the next five years. The results suggest that the decoupling trend in portfolio return is significantly influenced by factors such as rice cultivation, field rise, and soil management, which also contribute to the highest weighted risk. Additionally, this factor consistently shows the highest weighted importance in determining overall portfolio returns, as it also exhibits the largest marginal effects. Consequently, this investigation substantiates the detrimental influence of these variables on CO_2 emissions. Turkey's sustainable smart agriculture process is essential for the efficient expansion of the economy, as it integrates climate change considerations into current policies and initiatives and reinforces the policy indicator of economic consideration with environmental protection in terms of CO_2 emissions.

Keywords: Climate Smart Agriculture; portfolio return; environment; CO_2 emissions; Turkey

1. Introduction

Climate-smart agriculture (CLS-A) reduces CO_2 emissions by lowering the emission of CO_2 from agricultural activities and by helping to sequester CO_2 from the atmosphere [1]. Cover cropping practices, which sequester and add CO_2 into the year-end soil organic matter, and enhanced grazing management, which increases carbon storage in CLS-A [2]. First, Turkey is very susceptible initially to environmental changes [3]. It is in the Mediterranean basin, where increases in temperature as well as droughts due to extreme weather are already happening [4, 5]. Furthermore, CLS-A helps to shape the economy and uses sustainability in agriculture. As such, CLS-A practices resilience and buffers environmental change, making food security and a stable economy possible. Secondly, the huge role of GHG emissions (including CO_2 emission [6]. By implementing strategic CLS-A measures that will reduce emissions and support soil management and agroforestry, Turkey can significantly contribute to its efforts to meet and transition to a sustainable economy. In this case, the decoupling of CLS A development and rapid production from harmful processes of the environment is obtained. Correspondingly, it provides a framework for Turkey to modernize and reinforce its CLS-A sector and thereby benefit the environment and economy [7, 8].

The CLS-A is a holistic approach designed to transform agricultural systems to effectively and sustainably support food security and development under a changing climate. It integrates three main pillars: increased productivity, enhanced resilience, and reduced GHG emissions. Turkey, in a region highly vulnerable to climate change impacts, is increasingly focusing on CLS-A to address its agricultural challenges, ensure food security, and contribute to global climate goals. Turkey's agriculture sector is a vital source of economy and employment, but it faces some significant challenges regarding soil degradation, food security concerns, and GHG emissions. In case of soil degradation, threateningthe long-term sustainability [9]. The food security of agriculture has created an important role in reliance on imports and higher food prices. In 2023, the food inflation rates were over 55%, and the agriculture sector contributed 71.5 Mt CO_2 equivalent in 2023, which is an increase of 37.9% from 1990 [10]. We examine the environmental protection implications of CLS-A through agriculture, which involves the integration of trees into agricultural systems to achieve two objectives: the limitation of forest denudation and the sequestration of atmospheric CO_2 in biomass and soil [11, 12]. The CLS-A is also approaching productivity and income regarding agriculture, in which sustainable intensification and innovation systems play the most important role in climate, where climate-smart technology and practice control environmental degradation in the public and private sectors and help farmers to facilitate technology transfer and knowledge sharing [13]. Second, enhanced resilience shows that conservation agriculture and diversification of farming systems regarding the crops and livestock reduce the risk associated with climate impacts [14]. Third, mitigation is like reducing emissions by livestock, soil management, and land use management. Lastly, Turkey can move forward with the technological revolution in agriculture, and also the policy development and innovative financing mechanism will be crucial for the widespread adoption of CLS-A [15].

The CLS-A practice has two primary objectives: to minimize climate change and then maintain the sustainability of this aim [16, 17]. The other was the sustainable agriculture practice focusing on production methods that can diminish CO_2 emissions [17–19]. Moreover, the two main contributions demonstrated, by means of the application of decoupling techniques, the important contribution to CO_2 emissions as well as the vulnerability of climate change [20, 21]. This research argues that by decoupling, Turkey can meet its food security goals with limited environmental impacts [22]. To investigate and implement effective decoupling techniques, we need a multi-pronged approach that considers such technological developments

[23]. It may be possible to decouple these goals: by increasing food production, we can stop our environmental impact [24]. There are many ways to do this. Second, agricultural practices such as enhanced agricultural practices, including integrated pest management, conservation tillage, and precision CLS-A, have been tested to increase the crop yield while reducing water consumption, soil erosion, and dependence on pesticides [25]. Second, eco-friendly innovations: water for irrigation can be sourced from salt water, drought-resistant crops are grown, and a vertical farming approach is adopted, which will technically work around the constraints of available resources and lead to more efficient food production.

Third, a significant amount of food is wasted due to issues in the supply chain. Reducing food waste from farm to fork is one approach that can help with decoupling. Lastly, a significant portion of the food waste occurs throughout the supply chain. The results of this study suggest that the decoupling (portfolio returns) of the agricultural sector in Turkey could have a substantial influence on the reduction of CO_2 emissions and the improvement of food security. Decoupling can foster multifarious, sustainable, and fruitful agricultural methods by empowering producers to make decisions more in line with market forces. Turkey would benefit from implementing supplementary policies that encourage producers to cultivate crops using sustainable methods, thereby slowing down the process. Transitioning to a decoupled technique could be a substantial enhancement for Turkey in addressing the challenges of food security and high levels of CO_2 emissions [26]. This CLS-A research aims to improve low-cost, long-term farming systems. It could involve finding new sustainable food buyers or verifying fair trade [27]. As an outcome of decoupling, rural infrastructure, healthcare, and education are more readily accessible. Significant progress toward decoupling has been achieved through the implementation of waste-reduction techniques. By implementing these measures, Turkey can guarantee its food supply in the future and improve the global environment [28, 29].

This study emphasizes CLS-A's climate change adaptation through research. It underlines the need to focus on relevant research areas like drought management, climate change effects, adaptation, and evidence-based food security. It also emphasizes the need to collect and verify climate change data in each river basin and region. Authorities can affect future event preparations after accounting for the data. Research and development aims to improve land, water, and crop management in response to climate change. Thus, the literature emphasizes the following ideas and actions needed to reach this goal. This study emphasizes the need to study how climate change may affect CLS-A output, production, and local information. The main goal is to monitor climate change and create measures to limit its effects on agriculture. This study examines whether sustainable farming might reduce climate change's impact on Turkish food production. We investigate Turkey's environmental and economic policies using critical economic facts, global choices, and laws. According to studies, Turkish farmers can slow global warming by using modern farming methods. The legal application of Turkey's program reduces hazardous emissions, boosting economic growth and environmental security. Economic data can illustrate how financially feasible ideas are.

This study has two sections: background information and research on decoupling's environmental implications. Section 3 describes the research goals, theoretical and conceptual frameworks, and methodology and results. Estimation and results are in Section 4. Section 5 addresses results. The conclusion summarizes the investigation's findings and suggests relevant policies.

2. Literature Review

2.1. Climate Smart Agriculture

A number of sustainable CLS-A-related policies and projects have been established by the Turkish government [30, 31]. However, additional efforts are necessary to ensure the effectiveness of these policies and initiatives. To create new sustainable agricultural technology,

the agricultural sector must also engage in research and development. How Turkey deals with its CO_2 emissions problems will determine the fate of its CLS-A economy [32]. In addition, the Turkish Ministry of Agriculture and Forestry (TMAF) has stated that the objective is to enhance the sustainability of agricultural development and the competitiveness of agricultural food security. The CLS-A has three most critical components, which provide a comprehensive global perspective: productivity with food security and income, the adoption and development of resilience to environmental change, and the reduction of CO_2 emissions, as clearly identified by a framework [33]. As long as the solutions are within reasonable costs, individuals who are responsible for molding policies and farmers can identify the methods that effectively capture the most CO_2 emissions [34]. It is imperative to investigate the factors that influence individuals' decisions to adopt ecological and dining behaviors. [35]. More sustainable methods can help Turkey's CLS-A endure the impacts of climate change, water scarcity, and soil degradation [36]. Consequently, this sector will persist in the future, guaranteeing that the Turkish populace has an adequate supply of sustenance [37]. The sustainable growth also supports the transition to transformative change in smart agriculture through a variety of initiatives, such as innovation, capacity development, and the technological revolution in the context of smart farming. Currently, there is insufficient information available to determine whether various decoupling strategies are the most effective in mitigating the impact of climate change on CLS-A. Furthermore, it is imperative to conduct additional research to determine the most cost-effective decoupling solutions [38]. Research is necessary to develop effective laws that promote environmental protection, prevent detrimental actions, and ensure equal opportunity in agriculture [39, 40]. Additionally, research is necessary to investigate the consequences of policies that are currently in effect and planned for the future [26].

2.2. Sustainable practice of Turkey and CO_2 emissions

Turkey can address these challenges and fill these knowledge gaps by implementing a successful decoupling strategy that is financially feasible, socially acceptable, and sustainable. It guarantees the availability of food for individuals and guarantees the sustainability of food systems in the future. In spite of the adoption of CLS-A, there are still certain research areas in it that require improvement to achieve a more favorable outcome and acceptance. Currently, there are numerous critical regions in the globe [41]. It is imperative that we determine whether CLS-A is economically feasible for small and medium-sized farms in Turkey [42]. To facilitate the successful implementation of sustainable practices that capitalize on emerging technologies, it is imperative that farmers receive assistance in improving their digital literacy [43]. Subsidies and tax breaks, which are informed by research, are two policies that have been effective in motivating farmers to embrace sustainable practices [44]. In order to incentivize producers to embrace market mechanisms, sustainable practices, such as certification systems or premium pricing for sustainably produced food, could be investigated. Filling these knowledge shortfalls in agricultural sustainability can hasten Turkey's shift to ecologically friendly farming practices [45].

3. Research Methodology

3.1. The CLS-A Method and Decoupling

A dynamic development model of Turkey's global economy was employed in this segment to examine the interplay between food security and the environment. In this section, we analyzed decoupling as a potential solution to improve food security and reduce CO_2 emissions in Turkey. In the context of agriculture and CO_2 emissions in the environmental factors, the fundamental approach of this study is to decouple the determinants of portfolio return. First, the portfolio technique considers environmental factors and agricultural shocks as random market risks, thereby isolating their impact. We directly attribute the climate events—

droughts, food, consumer preferences, agriculture policies, and sustainable agriculture products in this portfolio—through the decoupling procedure. Consequently, this method is more detailed in its ability to comprehend the fundamental portfolio change in the context of high- and low-weighted risks in numerous, continuously inundated, and single aeration scenarios. Secondly, this technique also enables us to gain a more comprehensive understanding of the environmental degradation caused by CO_2 emissions in the agricultural system. Specifically, we can directly distinguish the performance of sustainable farming practices that are influenced by commodity market prices. Thirdly, we examine the investment strategies within the agricultural sector. Develop more targeted risk mitigation strategies for environmental and agricultural vulnerabilities through portfolio returns. In this regard, it is straightforward to distinguish between the financial performance that is influenced by the market's fluctuating prices and that that is influenced by the improvement of agricultural practices that provide the most optimal balance between economic viability and environmental sustainability. This can be achieved by reducing CO_2 emissions for a given level of economic return, or vice versa. Additionally, this method provides an informative tool for the determination of environmental policies, agriculture trade, resilience, and allocation. Furthermore, it has been consistent with sustainable development and market conditions, which has facilitated the development of a portfolio decoupling technique that is designed to reconcile environmental resilience with financial performance.

We have made some modifications to the decoupling technique and have discussed them in the context of decoupling methods for controlling energy output and ingestion. This work is a continuation of previous studies [46–48]. The economic exercise is not instantaneously disregarded on the basis of decoupling, as the previous research investigation on decoupling strategies [46] reveals a one percent change in each year and their predicted change regarding decoupling. Given that the robustness and relationship have already been established through decoupling and subsequent portfolio turnover, which is a modification of research methodologies based on the decoupling methodology. The portfolio return has been estimated following the computing decoupling, and it is possible to predict the next five years and determine a one percent shift in CO_2 emissions using other indicators.

This text begins with an examination of decoupling, followed by an estimation of portfolio return using weighted risk and return. Finally, we will discuss the fluctuation of this return. [47]. The negative portfolio return value represents the separated decoupling values. Consequently, for each category, the returns of the portfolio can be examined for a complete eight years [46]. The last step is to examine the variation in portfolio returns for every group. The next four steps make up the computation. At the onset, we make use of beta decoupling to ascertain the average transformation of the environment as well as the percentage change for each group individually. This implied the elastic range decoupling condition. Initially, indicators are evaluated by multiplying the covariance and variance by 8/7 and 1%, respectively. Next, portfolio returns are calculated with high and low weighted risk by decoupling positive and negative attitudes. The weighted risk indicated that the total risk and average return were contained in the weighted portfolio return. Consequently, the highest and lowest attitudes of decoupling in this weighted risk model result in a 1% change in CO_2 emissions. This 1% change in decoupling has caused a 1% shift in the following areas: rice cultivation, enteric fermentation, field fire, managed soil, urea application, and manure management. The second step involves determining the average return and overall risk for each cohort after decoupling [49, 50].

The mean return offers details about the conventional assessment, while the total risk indicates the standard deviation for each group over a period of eight years. Furthermore, the portfolio return with weighted risk is computed by adding the 100% risk premium to the adjusted return indicators of reduced environment, namely field fire, managed soil, urea

application, manure management, enteric fermentation, and rice cultivation, subsequent to the average return and total risk [51, 52]. A 1 percent change in the environment is then computed using the projected weighted return and risk of each cohort. In Section 3, the effects of the environment and the impact of a 1% change in food security on CO_2 emissions degradation are also evaluated individually. Moreover, the decoupling shift at different junctures is ascertained by the groups [53]. For instance, a 1% shift in environment decoupling can be attributed to modifications in CLS-A, which encompass enteric in the field burning, managed soil, urea application, manure management, enteric fermentation, and rice cultivation [54].

3.2. Sources of the Dataset

From 1992 to 2023, this study examines the CO_2 emissions implications of different agricultural techniques in Turkey, such as field burning, managed soil, urea application, manure management, enteric fermentation, and rice production, with the assistance of decoupling techniques. It then independently evaluates the portfolio returns using high and low weighted risk. The decoupling operation divides into four distinct sections (A–D) for the purpose of analysis. All of the subsets cover a full five years. Our Data in the World and the World Bank (WB) both have datasets that we analyzed using panel data (Appendix A). Inspired by eco-economic separation and a byproduct of techno-nationalism, accessibility is a determining element in the metrics' selection. The primary objective of this research is to assess the impact of CLS-A on the decoupling of CO_2 emissions in a statistically valid sample of Turkey. The significant increase in urea application in 2020 could be due to a combination of factors. These might include new government policies or subsidies that incentivized greater fertilizer use, an exceptionally favorable agricultural season that demanded more nutrients, or economic shifts such as a decrease in urea prices or an increase in crop prices making higher application more profitable. (Appendix B).

This increase is mostly attributable to the country's heavy use of coal to meet its growing energy demands. We investigate how these nations' actions affect decoupling on a global scale [55]. Based on their ecological situation and agricultural progress, we have ranked the nations [56]. Because of the association between reliance on these resources and volatility, it is widely believed that the price environment for commodities derived from them threatens the nation's purpose of sustainable economic growth. We determine that changes in the response variables are due to a decoupling estimate ranging from +1 to -2, which is a surrogate for measuring the effectiveness of agricultural initiatives to reduce CO_2 emissions harm [57]. The decoupling concept is further explained by looking at the performance of the portfolio. Conversely, if the decoupling analysis turns out to be unfavorable, we use techno-nationalism to rein in the CO_2 emissions decoupling effect and come up with workable solutions for the agricultural sector's transformation. The decoupling method was used to conduct a weighted calculation [58]. It demonstrates that as environmental conditions change by one percentage point, field burning, managed soil, urea application, manure management, enteric fermentation, and rice cultivation all change proportionately. The weighted values with a flexible range became readily apparent when distinct attitudes were uncoupled [59].

3.3. Portfolio Returns

The efficiency of six key CLS-A indicators—managed soil, field burning, urea application, enteric fermentation, manure management, and rice cultivation—is examined in this study. We calculate the portfolio return (average return and total risk) for each of them under three different aeration regimes. Through four distinct categories: continuously flooded, single aeration, and multiple aeration in groups (A to D). Thus, we use the current scientific

knowledge of these activities to interpret the predicted effect on the environment by CO_2 emissions, which is drawing a connection between these indicators. Furthermore, the portfolio returns indicate things like yield, operational risk, profitability, weather-related hazards, and input cost variations. This is different from financial market indicators like decoupling or risk premiums, which show how investors perceive the risk and return in the capital markets as a whole. A new dataset that includes these financial metrics or a theoretical framework that explicitly links the market-level financial performance of agricultural ventures to their greenhouse gas emissions would be needed to set up a direct, empirically observed causal relationship between these particular financial market indicators and CO_2 emissions. For the first time, we have the data needed to assess the potential environmental impacts, risks, and economic feasibility of individual agricultural practices on individual farms. But when looking at the economy and finance as a whole, it is possible to speculate about a correlation between financial indicators and CO_2 emissions; this is especially true when it comes to the explicit pricing of climate change risks on the market.

The CLS-A indicator's estimated weighted return value in the portfolio reveals how much of an impact the better decoupling approach has on the return. Two outcomes could occur. The first is the disparity between the variable's Turkish investment breadths. The indicators over the next eight years are a further factor to consider. The weighted risk and possible return are determined using the consequences of the environment, with the shared aid of decoupling. There are three steps to the calculation process [60]. The average return and risk value variable is one technical evaluation statistic that differentiates the four countries' economies (A–D) [48]. The average return is calculated in this research using total risk and average beta decoupling. That is consistent with the CO_2 emissions standard deviation for the past eight years. Secondly, we determined the portfolio weight by using the relative relevance of the three performance measures (agricultural) to assign a value to each. We allot a certain percentage of the portfolio to each variable according to criteria like CO_2 emissions impact and economic development. The third phase relies on the concept of weighted rewards and risk [61]. This portfolio's enormous potential gains and losses are shown by the big swings. Additionally, the disparity between risk and weighted return is brought to light. To reduce CO_2 emissions risks to manageable levels, it is necessary to evaluate the viewpoints of the most risk-tolerant individuals, which can be achieved through changes in technology, changes in CLS-A, and strategic policies [62]. With this data, we were able to compute the average return and total risk, which we then adjusted using weighted percent. For the most part, previous studies have used a stochastic approach to explaining the portfolio selection problem, treating asset returns as random variables, and have assumed that the statistical feature of agricultural stock returns will remain constant [63]. Although decoupling can be advantageous, modeling portfolio selection under weighted return is problematic owing to the dynamic environment in different regions. In addition, multiply the total risk, average return, and estimated weighted value of each category by their corresponding values [64]. We calculate the weighted return value by averaging it. The risk value is an inevitable starting point for the weighted risk value. In this case, we estimate the total risk and average return as we approach the beta decoupling levels. It was able to accomplish this by taking a more solid and sincere approach to the percentage change in agricultural technology [63, 65].

Pollutants released into the atmosphere by fertilizer applications, manure management, and other agricultural inputs are all part of the agricultural inputs that contribute to the degradation of naturally occurring soils. Industrial waste products, such as crop stubble and straw, are associated with emissions from field burning [66]. One major contributor to GHG emissions is the widespread use of urea fertilizer in CLS-A production. Among the numerous agricultural sources, such as manure management, field burning, urea application, enteric fermentation, and agricultural soils, among many others, urea—a nitrogen-based fertilizer—

can release nitrous oxide, a potent CLS-A. All emissions from agricultural sources add up to total CO_2 emissions. [67–70]. The findings unequivocally demonstrate that emissions in each of the four categories have exhibited a progressively increasing trend over the course of the years. The emissions from managed soils have registered the most significant increase, which is most likely attributable to the increased application of fertilizers and manure to improve crop yields [71]. In addition, the data clearly demonstrate that CLS-A plays a crucial part in contributing to Turkey's overall CO_2 emissions profile. In the year 2020, the agriculture sector was responsible for around twenty percent of the total emissions that were produced by the nation. The information presented in Appendix A provides a rundown of the CO_2 emissions that will be produced by Turkish CLS-A in the year 2020 [72]. One of the most significant contributors to carbon dioxide emissions within Turkish CLS-A is enteric fermentation, which accounts for approximately forty percent of the overall emissions [73]. The second-biggest source of carbon dioxide emissions from Turkish CLS-A, after manure management, accounts for about 25% of the overall emissions [74]. Animal manure has the potential to release two powerful greenhouse gases—methane and nitrous oxide—during the storage, processing, and application phases of manure management. Furthermore, for Turkey's CO_2 emissions, CLS-A soils account for more than 15% of the overall total. Agricultural soil organic matter cultivation and nitrogen fertilizer use both contribute to the formation of nitrous oxide, the gas responsible for these emissions [75].

3.4. Portfolio Equations

The weighted portfolio return was calculated for each agriculture management practice and strategy in various groups under four varying aeration conditions in terms of weighted risk (total risk) and weighted return (average return) in the portfolio return. Initially, weighted risk suggests a higher degree of unpredictability in the high- and low-risk zones. Similarly, the environmental impact and economic outcomes are subject to variation. In the event of economic outcomes, the potential for eccentricities from the average return is unstable yield and volatile profits. Additionally, the environmental impact—CO_2 emissions—is highly inconsistent across the various numbers of years in groups, which poses a high risk. Secondly, the agricultural strategies' weighted return outcomes are linked to both economic and environmental advantages. For example, a 1% modification in strategies results in an increase in profit, which in turn increases the return. The environmental strategies in terms of CO_2 emissions indicated that an average reduction and contraction in the environment are represented by a change of 1% in predictor variables. In the agricultural system, the average condition, such as continuous flooding and single and multiple aerations, is relevant in predictor variables that substantially influence CO_2 emissions. In the context of portfolio return, the most frequently used formulations for weighted average return and risk are as follows:

The weighted average return is denoted by $R_{i,j}$ (CO2 emissions) of agriculture strategy i under the condition of aeration, which is specified with i over n observed periods (Groups A-D) in Eq. 1. The weighted return specify with continuous flooding for managed soil, field fire, urea application, enteric fermentation, manure management, and rice cultivation is calculated as:

$$Weighted\ Average\ return_{i,j} = \frac{1}{n}\sum_{k=1}^{n} \mathrm{R_{i,j,k}} \qquad \text{Eq.1}$$

Eq. 2 illustrates the dispersion of individual outcomes in relation to the weighted average return, as denoted by

$$Weighted\ risk = \frac{1}{n\sum_{k=1}^{n}(\mathrm{R_{i,j,k}} - Weighted\ Average\ return_{i,j})^2} \qquad \text{Eq.2}$$

The most prevalent portfolio return formulations, which are calculated on the basis of decoupling, are the weighted average and weighted risk. The weighted risk in Eq.2 is typically represented by the σ of the return $R_{i,j,k}$, which is a measure of the dispersion of individual outcomes around the $Weighted\ Average\ return_{i,j}$. The agricultural strategy outcomes are displayed with the same value as i under the aeration condition j during a specific period “k”, and the total number of periods is represented by “n”. The individual effects of the individual predictor variables, such as managed soil, field burning, urea application, enteric fermentation, manure management, and rice cultivation, on the portfolio return have been demonstrated in terms of continuous, single, and multiple aeration.

4. Results

We used data for Turkey's nation from 1992 to 2023 and the portfolio return concept to look at how a resurgence with repeated and single aeration and continuous flooding affected high- and low-risk zones. The portfolio return is computed on the basis of the decoupling technique. Specifically, it is demonstrated that a 1 percent change in multiple and single aeration and continuous flooding leads to a 1 percent change in CLS-A. In Fig. 1, we can observe the pattern of wheat yields in Turkey throughout the four categories (A-D). The formatting condition for all CLS-A figures in this portfolio is evident in the returns, which feature three marks: a checkmark, an exclamation mark, and a heavy cross mark. The exclamation mark is recorded when the result is greater than 70%, and a hefty cross is indicated when the result is greater than 30%. The checkmark indicates a decoupling attitude of less than 70%. Furthermore, the marks indicate issues related to field burning, managed soil, and urban development within the CLS-A sectors [76, 77]. The increase from 2.4 metric tons/ha in 2013–14 to 2.5 metric tons/ha in 2022–23 indicates that wheat yields have been rising consistently. We expect wheat yields to rise even further in 2023–24, reaching 2.7 tons per hectare. Fig. 1 reveals that Turkey's wheat area remained virtually unchanged at 7,020,000 hectares (ha) between 2015/16 and 2019/20, compared to 7,860,000 ha in 2016/17. However, the wheat harvest has been more unpredictable, fluctuating between 15,000 metric tons in 2014–15 and 21,000 metric tons in 2017–18. The projected wheat harvest for 2023–24 is 19,500 million metric tons.

4.1 High-Risk Zone of CLS-A

According to Fig. 1, the portfolio's CLS-A needs are assessed for multiple and single aeration and continuously flooded separately in high- and low-risk zones. Firstly, we analyzed nations with a high-risk factor. In our assessment, along with each country's efforts to combat climate change, we discovered that new technical regulations impact the CLS-A separation characterized by three separate structural marks.

Similarly, in Fig. 1, portfolio-weighted results of more than 70% are shown by the exclamation mark in high- and low-risk countries, respectively. The portfolio shows the formatting condition with a checkbox, exclamation point, and heavy cross mark. Checkmarks indicate less than 70% decoupling, exclamation marks more than 70%, and heavy crosses more than 30%. In terms of total risk and average return in weighted portfolio return, the largest decoupling attitude is reported in Turkey (0.248% and 1.249%) and in field burning (0.567% and 0.297%) in groups D and C with indicated heavy crosses, respectively.

			Managed Soil	Field burining	Urea application	Enteric fermentation	Manure management	Rice cultivation
Continously Flooded	Av.return	Group A	✓ 0.164	✗ -0.511	! -0.190	✓ 0.130	! -0.090	✗ -0.105
		Group B	✗ -0.197	✓ -0.047	✓ 0.052	✗ -0.007	✗ -0.526	✗ -0.215
		Group C	✓ 0.202	✗ -0.394	✗ -0.396	✓ 0.099	✓ 0.204	✓ 0.154
		Group D	✗ -0.135	✓ -0.106	✓ -0.073	✗ -0.004	✓ 0.096	✓ 0.042
	Total Risk	Group A	✓ 0.064	✓ -0.023	✗ -0.047	✓ 0.455	✓ 0.237	✗ -0.035
		Group B	✓ 0.011	✓ 0.104	✓ 0.118	! 0.067	✗ 0.015	✓ 0.248
		Group C	✓ -0.012	✗ -0.358	! 0.025	✓ 0.231	! 0.118	✗ 0.000
		Group D	✗ -0.178	! -0.058	! 0.018	✗ -0.341	! 0.094	✗ -0.063
Single Aeration	Av.return	Group A	✗ -0.924	! 0.624	! 0.382	! 0.214	✓ 0.805	! 0.337
		Group B	✓ 0.526	✗ 0.192	✗ 0.105	✗ 0.100	✓ 0.475	✗ 0.240
		Group C	✓ 0.292	✓ 0.993	✓ 0.856	✓ 0.386	✗ -0.607	✗ 0.239
		Group D	✗ -0.491	✗ 0.357	✗ 0.130	✗ 0.034	✓ 0.400	✓ 0.442
	Total Risk	Group A	! 0.045	! 0.055	✗ -0.119	✓ -0.105	✓ 0.066	✓ 0.090
		Group B	! -0.122	✗ 0.000	✗ -0.127	✓ -0.157	✓ -0.003	! -0.171
		Group C	✓ 0.373	✗ 0.047	✓ 0.074	✓ -0.125	✓ -0.014	✓ -0.030
		Group D	✗ -0.553	✓ 0.147	✗ -0.177	✗ -0.318	✗ -0.178	✗ -0.360
Multiple Aeration	Av.return	Group A	✓ 0.331	✓ -0.273	✓ 0.129	✓ 0.139	✓ 0.556	✓ 0.401
		Group B	✓ -0.045	✓ 0.055	✓ -0.119	✓ -0.105	✓ -0.066	✓ -0.090
		Group C	✗ 0.292	✓ 0.993	✓ 0.856	✗ -0.386	✓ 0.607	✗ 0.239
		Group D	✓ 0.491	✗ 0.357	✗ 0.130	✓ 0.034	✗ -0.400	✓ 0.442
	Total Risk	Group A	✗ -0.122	✓ 0.000	✗ -0.127	✗ -0.157	✓ 0.003	✗ -0.171
		Group B	✓ 0.373	✗ -0.047	✓ 0.074	✓ -0.125	✗ -0.014	✓ -0.030
		Group C	✓ 1.249	✓ 0.567	✗ 0.108	✗ -0.570	✓ 0.456	✓ 0.244
		Group D	✗ 0.248	✗ 0.297	✓ 0.238	✓ 0.930	✗ 0.423	✗ 0.194

Fig 1: Portfolio return of multiple and single aeration and continuously flooded in high-risk zone.

Similarly, rice cultivation has the strongest attitudes toward (0.194% and 0.244%) and enteric fermentation in single aeration (-0.318% and -125%) in group C with the indicated heavy cross and checkmark. In the same scenario, the urea application in group D was 0.108%, and the corresponding value was 0.238. The managed soil decoupling attitudes demonstrate -0.553% and 0.373% in single aeration in groups C and D with heavy cross and checkmark, respectively [58]. The portfolio returns have been calculated using three dimensions: continuously inundated, single aeration, and multiple aeration. In groups C and D, the rise in cultivation is 0.154 and 0.042, respectively, with a checkmark in the average return. However, the total risk of continuously flooding is estimated to be 0.000 and -0.035, with a heavy cross, indicating the least decoupling. This demonstrates that a 1 percent change in continuous flooding results in a 1 percent change in CLS-A. In the same scenario, the single aeration result of rice cultivation indicated that the average return was 0.239 and 0.442 with a heavy cross and checkmark, and the total risk was -0.171 and -0.030 with an exclamation point and checkmark in groups B and C. It has been shown that a 1 percent change in single aeration results in a 1 percent change in CLS-A, which is comparatively higher than continuously flooded. The results of the managed soil also showed that the highest decoupling change was in groups A and B, with values of 0.045 and -0.122, respectively, as indicated by the exclamation marks. This indicates that CLS-A exhibited the highest decoupling change.

Hence, CLS-A from managed soils includes emissions resulting from the cultivation and management of agricultural areas, which encompass fertilizer applications, manure management, and other agricultural inputs. The combustion of crop leftovers, such as straw and stubble, is associated with field burning emissions [78]. Use of urea fertilizer in agricultural cultivation is the source of urea application emissions. The nitrogen fertilizer urea can release nitrous oxide, a powerful CO_2 emission. Emissions from all agricultural activities, such as manure management, field burning, urea application, enteric fermentation, and agricultural soils, make up CLS-A's total emissions.

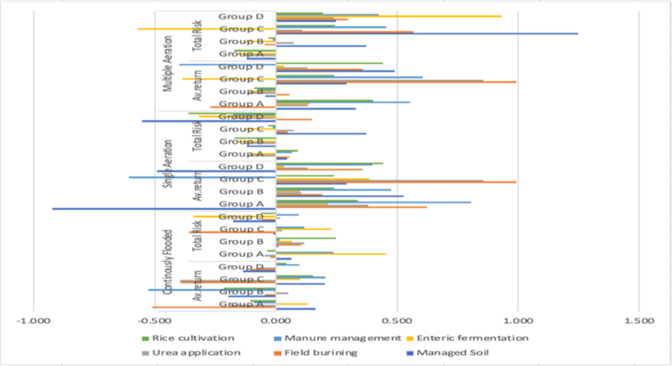

Fig 2: Portfolio return of multiple and single aeration and continuously flooded in high risk

Fig. 2 demonstrates that growing rice releases a lot of carbon dioxide. The predicted 2020 CO_2 equivalent emissions from rice agriculture were 261.53 million tons, as shown in the table. All told, this accounts for around 1.4% of the world's CO_2 emissions. Compared to intermittently flooded rice agriculture, continuously flooded rice cultivation produces greater carbon dioxide emissions [79]. This is due to the fact that, in anaerobic conditions, methane is more readily produced in continually flooded rice paddies, making them a powerful source of carbon dioxide emissions [80]. Over the years, emissions from rice farming have been on the rise. There has been a roughly 50% rise in emissions from rice cultivation since 1990, as seen in the table. Several factors have contributed to this, such as the rise in fertilizer use, the extension of rice agriculture into previously uncultivated areas (such as wetlands), and the overall increase in rice production [44].

4.2 Low-Risk Zone of CLS-A

Fig. 3 illustrates the portfolio return of low-risk countries weighted by risk and return with varying ratios in the CLS-A sector. Initially, the approximated results are analyzed for both single and multiple aerations and continuous flooding in the low-risk zone.

			Managed Soil	Field burining	Urea application	Enteric fermentation	Manure management	Rice cultivation
Continously Flooded	Av.return	Group A	0.038	-0.058	-0.042	0.694	-0.280	-0.111
		Group B	-0.203	0.277	0.097	-0.045	-0.036	-0.043
		Group C	0.126	0.045	0.023	-0.479	-0.574	-0.206
		Group D	-0.467	0.108	0.478	-0.663	0.264	0.255
	Total Risk	Group A	0.278	0.414	0.141	1.832	0.424	0.178
		Group B	0.708	0.742	0.168	0.052	0.217	0.175
		Group C	0.832	0.326	0.200	1.356	0.780	0.254
		Group D	0.834	0.891	1.079	0.668	0.525	0.434
Single Aeration	Av.return	Group A	0.187	0.023	0.141	-0.200	-0.353	-0.226
		Group B	-0.401	0.504	0.123	0.004	-0.347	-0.319
		Group C	-0.136	-0.085	-0.053	-0.317	-0.616	-0.114
		Group D	-0.021	0.125	-0.011	0.427	-0.337	-0.269
	Total Risk	Group A	0.402	0.448	0.566	0.813	0.355	0.261
		Group B	1.043	1.344	0.288	0.081	0.771	0.743
		Group C	0.159	0.107	0.054	0.774	0.800	0.595
		Group D	0.589	1.390	0.405	1.540	0.415	0.392
Multiple Aeration	Av.return	Group A	-0.026	-0.959	0.110	0.237	1.006	0.178
		Group B	-0.047	-0.028	-0.028	0.622	-0.012	0.486
		Group C	-0.186	-0.167	-0.086	-0.085	0.064	-0.217
		Group D	-0.305	-0.004	0.162	-0.252	-0.810	0.039
	Total Risk	Group A	0.231	1.169	0.366	0.680	1.826	0.614
		Group B	0.139	0.275	0.076	1.154	0.318	0.899
		Group C	0.791	0.490	0.245	0.566	0.216	0.287
		Group D	0.751	0.693	0.230	0.393	1.079	0.225

Fig. 3: Portfolio return of multiple and single aeration and continuous flooding in a low-risk zone.

The maximum weighted risk in group D is estimated to be field burning (1.390), multiple aeration (0.693), and manure management (1.079) in China, a high-risk country [81]. From about 70% in 2000 to about 75% in 2019, the percentage of CLS-A in Turkey's water abstractions has been steadily rising. This data points to the fact that CLS-A is using more and more water in Turkey. Additionally, Turkey's irrigated land share has been steadily rising, from about 20% in 2000 to about 25% in 2019. This indicates that irrigation is becoming more important for Turkish farmers when it comes to crop production [82]. Throughout the years, CLS-A's contribution to Turkey's CO_2 emissions has remained around 20%. Therefore, CLS-A probably isn't a big deal when it comes to Turkey's CO_2 emissions. From about 10% in 2000 to about 8% in 2019, CLS-A's proportion of Turkey's overall energy consumption has been steadily declining. This data points to an increase in CLS-A's energy efficiency in Turkey. It has been more common to take phosphorus from the soil in Turkey than to add it, resulting in a negative phosphorus balance since the year 2000.

It appears that the soils of Turkey are losing their phosphorus content. Since the year 2000, Turkey has also had a negative nitrogen balance, which means that the country is removing more nitrogen from the soil than it is adding. This indicates that the nitrogen levels in Turkey's soils are declining. These conclusions indicate that CLS-A in Turkey is facing difficulties related to water scarcity, soil degradation, and climate change. The Turkish government must take action to address these concerns if CLS-A continues to operate in the future. Consequently, the utilization of rice and urea has resulted in a 0.392 and 0.405 reduction, respectively, in agricultural productivity within group D. In the low-risk zone, group C has the highest weighted risk at 0.774, which is 1.98 times higher than group D's 1.540. Since 1990, when it was 37.9 million hectares, the irrigated area for growing rice has grown significantly, reaching 125.4 million hectares in 2020 [83]. From 1990 to 2020, the estimated emissions from rice agriculture have climbed continuously, reaching 261.530 million tons of CO_2 emissions equivalent. Figure 4 demonstrates that rice cultivation has the greatest attitude in single aeration, which is vital because rice is both a staple crop for many people's diets and a big agricultural crop in the country. Turkish rice yields have steadily increased over the previous decade, rising from a respectable 7.6 metric tons per hectare in 2013–14 to an impressive 8.30 metric tons per hectare in 2023–24. There are three major reasons for this impressive expansion. First, let's discuss the technological advancements [84]. Modern seed types, precision CLS-A methods, and integrated pest control concepts are just a few examples of how Turkish farmers are fully embracing cutting-edge agricultural technology [85].

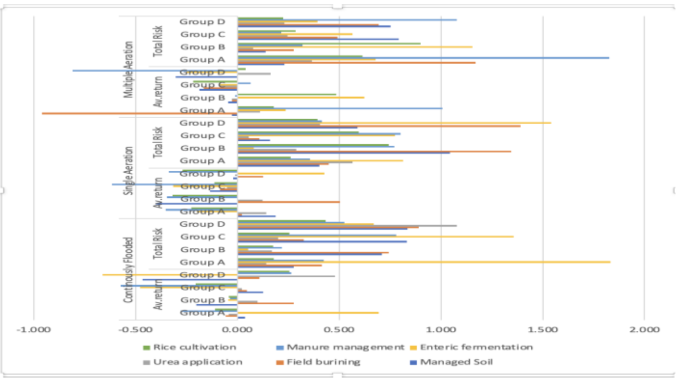

Fig 4: Portfolio return of multiple and single aeration and continuously flooded

There is no denying that these technological advancements have been the foundation of the increased rice harvests. Secondly, backing from the government: The Turkish government has done an admirable job of becoming a reliable backer of rice farming, offering

crucial assistance in the form of subsidies, loans, and outreach programs. Thanks to this strategic backing, the nation's rice farming has become much more efficient and productive [86]. Thirdly, beneficial climatic conditions have emerged; recently, Turkey's climate has been highly favorable for rice cultivation. The positive dynamics of CO_2 emissions have created an enabling environment for the enhancement of rice yields. Figure 5 illustrates that under group D, the Turkish economy and agricultural landscape have benefited greatly from the increasing rice yields achieved through enteric fermentation, manure management, and rice planting. Increased yields allow farmers to extract more rice out of their land, which improves their financial situation. Additionally, Turkish rice gains a competitive edge on the world stage due to a dramatic decrease in production overheads brought about by an increase in yields.

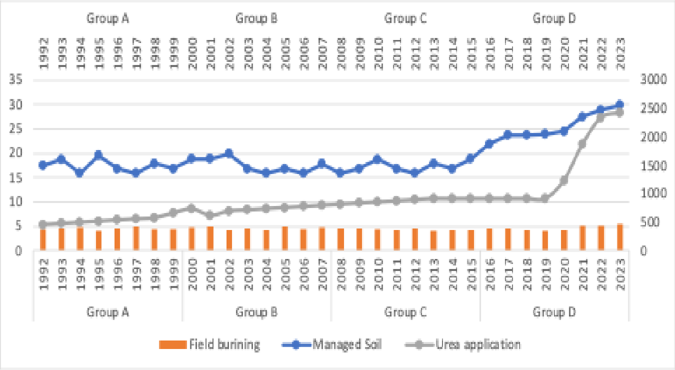

Fig. 5: Managed Soil, Field Burning, and Urea application

Like the rest of the world, Turkey is facing the daunting prospect of climate change as it pertains to agricultural output. Our farming community may have to adjust to changing weather conditions and look into new agricultural technology as a safety net if we want to survive this enormous challenge and keep or increase our rice harvests for the future. Agricultural activities such as liming, field burning, and rice planting also contribute to emissions. Additionally, the table highlights how the agricultural sector plays a major part in Turkey's CO_2 emissions profile, accounting for around 20.21 percent of the overall emissions. From the table, we may deduce several important things.

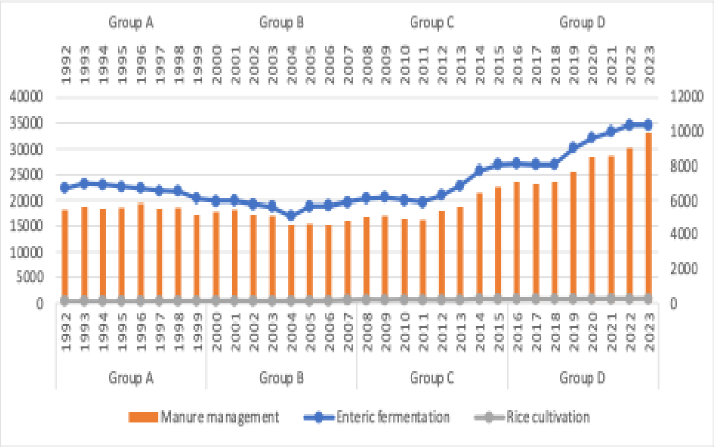

Fig. 6: Enteric fermentation, manure management and rice cultivation

The critical need to decrease emissions the data emphasizes the critical importance of the Turkish agricultural industry reducing its CO_2 emissions immediately [87, 88]. To lower Turkey's total carbon footprint and achieve its goals for mitigating climate change, this step is

crucial. Measures to decrease emissions: the agricultural sector in Turkey can decrease its CO_2 emissions by implementing a number of proven solutions. Improving manure management, reducing nitrogen fertilizer use, and switching to more environmentally friendly farming methods are all part of these steps [66].From 2014 to 2023, the line graph in figure 6 displays the maize yields in Turkey. Crop yields for maize have been trending upwards for a while now. This trend is a response to climate change and aims to secure a future food supply, which ranks somewhat higher in group D for growing rice. Rising rice harvests in Turkey are an encouraging trend [66]. Improving efficiency and output while capitalizing on favorable weather conditions is a hallmark of the remarkable transition that the country's rice farmers are presently experiencing. But we must not lose sight of the fact that increasing rice yields may not be sustainable forever [67].

In addition, the sustainable development of Turkey's agricultural sector is demonstrated by the diverse agricultural practices in terms of portfolio return under varying aeration conditions. This development has the potential to significantly enhance food security and reduce the sector's CO_2 emissions impact in terms of portfolio return. The CLS-A need is assessed for multiple and single aeration and continuously flooded separately in high- and low-risk zones with average and total risk. The portfolio returns the value of soil and agriculture, as indicated by the estimated results. Additionally, the portfolio return has the potential to promote the development of productive, sustainable, and diverse agricultural practices in Turkey by enabling producers to make decisions that are more in accordance with market forces, thereby improving food security. The estimated results are also consistent with the stated objectives, which promote sustainable methods and fruitful cultivation, as well as the reduction of environmental degradation caused by CO_2 emissions in Turkey. Additionally, the practice of enteric fermentation, urea application, and manure management directly correlate with greenhouse gas emissions, providing an empirical foundation for evaluating the potential of a unique method to contribute significant CO_2 emissions. Consequently, the technological revolution and policy implications in agricultural methods are slowing down the process of emissions. Ultimately, these practices yield positive returns while reducing risk, providing dynamic evidence to inform sustainable policies that encourage producers to adopt more market-aligned methods and promote sustainable development, thereby contributing to both the goals of emission reduction and food security.

5. Discussion

We are observing that the frequency of droughts, floods, and heat waves is increasing, and they are inflicting greater damage with each occurrence. It is presenting producers with additional obstacles in the areas of animal husbandry and plantation. The data in the image is not reliable for scientific interpretation as a result of the modifications. It is imperative to possess the most recent data on CLS-A to make informed decisions regarding agriculture [89]. Adopting CLS-A practices is necessary to mitigate the consequences of climate change on food security and nutrition [40, 90]. These methods facilitate the integration of food production and climate change adaptation, thereby providing a means to enhance food security [91]. The CLS-A program presents a critical opportunity to improve the fortunes of smallholder farmers in Turkey, including limited land and the effects of climate change. Some of the most significant CLS-A strategies are the promotion of regenerative agriculture, crop diversification, biochar application, and agroforestry, all of which enhance soil health and decrease greenhouse gas emissions [31, 92]. Both crop yield and nutrient content are directly influenced by soil health, which is fundamental to crop nutrition. By providing farmers with climate-resilient technologies and sustainable practices, such as precision agriculture and regenerative agriculture technologies, we can assist in the reversal of diminishing food quality trends [93].

Household incomes can be increased, self-sufficiency in food production can be improved, and food security can be strengthened because of the increased agricultural productivity that CLS-A has achieved. CLS-A practices are more likely to result in improved food consumption, increased dietary diversity, and enhanced overall food security in households [93].

Additionally, the food supply and food markets are at risk due to climate change; however, these risks can be mitigated by enhancing producers' adaptive capacity and improving resource use efficiency [94]. It is imperative to adopt an integrated strategy for mitigating climate change, which involves collaboration at the local, national, and international levels. Comprehensive economic, social, and environmental strategies must be implemented by governments and stakeholders to enhance climate risk management, mitigate vulnerability, and enhance resilience [95]. As a result, the farmers have been unable to expand their options for livestock and produce cultivation. The modifications have rendered the information in the image unsuitable for scientific research. Accurate and up-to-date information regarding CLS-A should be the foundation for any modifications to agriculture policy and practice [96]. Turkey is a prominent producer of wheat, and it is a staple in the cuisines of numerous individuals worldwide [97]. Consequently, producers are applying an increased quantity of fertilizers to the soil [98].

Farmers' adaptive capacity and resource utilization efficiency can be improved to mitigate the risks associated with climate change, which disrupts food markets and threatens the food supply [90]. An integrated strategy that involves collaboration at the local, national, and international levels is critically important for mitigating climate change in Turkey [99]. The implementation of comprehensive economic, social, and environmental strategies by governments and stakeholders is essential for the enhancement of climate risk management, the reduction of vulnerability, and the increase of resilience [100]. Climate-resilient pathways should prioritize evidence-based solutions, strengthen local institutions, harmonize agricultural and climate policies, and integrate climate and agricultural financing, as noted by the work. By emphasizing adaptable and context-driven solutions, this integrated approach is distinguished by its innovative policies and financing mechanisms [101].

Numerous Turkish farmers are currently employing various techniques, including crop rotation, to not only maintain the soil but also to prevent the excessive use of water. A further contributing factor to Turkey's record-breaking wheat harvest was the favorable weather [102]. Turkey has recently encountered mild winters and an abundance of rainfall in the course of the growing season [103]. That is why growing wheat yielded such excellent results. [104]. An increase in wheat harvests is a promising sign for Turkey, especially when considering the worldwide need for food. Turkey's dependence on imports will diminish as its wheat production increases. The conflict in Ukraine is a contributing factor to the global food shortage; therefore, it is unnecessary to disregard this issue [105]. The Turkish government should guarantee that producers continue to receive support payments to assist them in enhancing their wheat harvest [106].

6. Conclusion

Turkey can reduce maize harvest risks by taking the following steps: The CLS-A sector can improve collaboration among many agencies, strengthen an important ministry of food, CLS-A, and livestock components, and take other positive actions to prevent climate change. We intend to disseminate information regarding the possible effects of climate change on CLS-A and the potential measures that can be implemented to mitigate the risks. Every one of these goals has its set of essential steps that will lead us to success. Our primary objective is to enhance interagency collaboration and develop capabilities. Secondly, we aim to improve the capacity to manage drought. The objective underscores the necessity of enhancing the capacity

of the committees and agencies that are responsible for the administration of agricultural droughts. A third point is that there are training and research programs in place to address the impacts of local and regional climate change. It is highly recommended that the ministries of food, CLS-A, and cattle, in conjunction with their affiliated institutions, establish a special department within their respective provincial organizations to address climate change-related issues.

To adapt to the CLS-A sector's impacts of climate change, it is crucial to coordinate Turkey's policies, strategies, and legislative framework, as stated in the objective. The goal of these programs is to promote sustainable farming practices while simultaneously ensuring the safety and security of our food supply. Incorporating climate change considerations into existing policies and initiatives is crucial for effectively addressing these issues. Second, it highlights the importance of conducting and expanding scientific studies and research to understand the impacts of climate change on CLS-A and to facilitate adaptation to these changes. The agricultural sector should increase its utilization of biofuels to mitigate environmental contamination. Furthermore, it is imperative to implement initiatives that prioritize financial incentives for organic agriculture, sustainable agricultural practices, and land conservation. Although the agricultural sector must decrease its reliance on fossil fuels and increase its utilization of renewable energy sources, the implementation of alternative renewable energy production methods, particularly hydroelectric systems, may result in environmental issues.

The paper focuses on the following main points. To begin, economic development goals are a common impetus for agricultural research and development projects, which have traditionally prioritized increasing production. This has led to less focus on how to adjust to a changing climate. Second, CLS-A's shift to climate revolution adaptation: The goal emphasizes the need to redirect research efforts towards adapting to climate change, especially those that focus on soil and water resource protection. Developing irrigation systems, adjusting production patterns, increasing crop diversity, and disaster management strategies are all necessary to address the impacts of climate change on CLS-A on a national scale, especially in relation to drought. The third component of the plan is socio-economic research. This study will focus on CLS-A, food, the environment, and rural development on a national scale. It will take climate change into account. The findings of this study will pave the way for innovative policies that encourage the expansion of the country's agricultural sector in response to climate change, and they will also assist in securing the livelihoods of specific communities, such as women farmers. The fourth aspect is ensuring long-term food security. The plan stresses building a food security system around data uncovered by R&D initiatives that account for the impacts of climate change. Aiming to meet customer expectations while responding to changing factors affecting CO_2 emissions, this system is constantly evolving. As for the fifth objective, building a database and information system, it highlights the importance of developing such a system at a regional or basin scale, or even nationally. Lastly, the scale effect is valid in Turkey, as evidenced by a positive economic globalization coefficient. Foreign direct investment in environmentally favorable and technology-intensive practices should be promoted, as these investments have the potential to contribute to economic growth and employment. Additionally, the application of several environmental standards in international business can contribute to the mitigation of environmental contamination.

As a result, the identification of appropriate CLS-A strategies for the reduction of GHG emissions and the preservation of crop productivity necessitates a thorough examination of local environmental factors, such as soil condition, climate, and their interactions with alternative management systems. Various sociocultural, economic, and demographic factors influence the adoption of CLS-A. Although practices such as crop rotation, integrated soil fertility management, crop diversification, and intercropping are frequently implemented, their

widespread adoption remains low as a result of limitations associated with farm size, infrastructure, equipment, machinery, irrigation access, extension services, and obtaining weather information. The adoption of CLS-A can be improved by providing producers with the necessary inputs, providing context-specific CLS-A technologies and training, and enhancing extension programs, as recommended by this study. The successful implementation of CLS-A practices can be substantially impeded by a lack of farmer awareness and expertise, as training is essential for connecting farmers with supportive institutions, reliable input suppliers, and access to credit facilities. This method can be revised on a regular basis to accommodate evolving climatic effects; it will facilitate the assessment and monitoring of the impact of climate change on agriculture.

Appendix A

https://data.worldbank.org/topic/agriculture-and-rural-development and https://ourworldindata.org/search?q=agriculture

Appendix B

		Managed Soil		Field burining		Urea application		Agriculture total		Enteric fermentation		Manure management		Rice cultivation		Agriculture total	
	Years	(Kt CO2 eq.)	(%)	(Kt CO2 eq.)	(%)	(Kt CO2 eq.)	(%)	(Kt CO2 eq.)	(%)	(kt COz eq.)	(%)	(kt COz eq.	(%)	(kt COz eq.)	(%)	(kt COz eq.)	(%)
Group A	1992	17.433	40.2	373	0.7	456	1.0	46541	100	22397	48.6	5436	11.8	100	0.2	46054	100
	1993	18.764	39.6	391	0.8	476	1.3	47654	100	23221	49.5	5657	12.1	100	0.2	46928	100
	1994	15.876	39.0	411	0.7	496	1.0	44987	100	23025	49.6	5533	11.8	100	0.2	46979	100
	1995	19.543	38.4	353	0.8	516	1.0	44651	100	22636	47.7	5597	11.8	101	0.2	47407	100
	1996	16.876	37.8	393	0.7	536	1.2	44651	100	22339	49.7	5793	12.9	100	0.2	44926	100
	1997	15.875	37.2	425	0.8	556	1.1	45761	100	21815	49.5	5523	12.5	113	0.3	44080	100
	1998	17.876	36.6	372	0.7	576	1.0	45871	100	21792	48.7	5570	12.4	126	0.3	44757	100
	1999	16.875	36.0	372	0.8	658	1.5	43988	100	20313	47.8	5166	12.2	124	0.3	42505	100
Group B	2000	18.876	35.4	408	0.8	733	1.7	44312	100	19890	45.5	5348	12.2	135	0.3	43720	100
	2001	18.888	34.8	415	0.7	617	1.5	43987	100	19963	45.1	5448	12.3	147	0.3	44276	100
	2002	19.876	37.9	357	0.8	696	1.3	47611	100	19234	45.4	5142	12.1	128	0.3	42332	100
	2003	16.876	40.2	395	0.7	715	1.4	42876	100	18714	46.9	5096	12.8	132	0.3	39894	100
	2004	15.875	39.6	357	0.7	735	1.4	48761	100	16975	45.1	4540	12.1	135	0.4	37608	100
	2005	16.876	40.2	416	0.8	755	1.5	42987	100	18874	46.5	4596	11.3	143	0.4	40558	100
	2006	15.875	39.0	380	0.7	775	1.4	42998	100	18969	45.9	4590	11.1	156	0.4	41298	100
	2007	17.777	38.4	412	0.8	795	1.3	43123	100	19680	46.4	4781	11.3	183	0.4	42439	100
Group C	2008	15.875	39.0	398	0.7	814	1.3	43211	100	20352	46.4	5027	11.5	212	0.5	43900	100
	2009	16.876	38.4	385	0.8	834	1.4	41765	100	20575	47.4	5081	11.7	203	0.5	43421	100
	2010	18.762	39.0	375	0.7	854	1.4	42987	100	20084	48.6	4929	11.9	216	0.5	41302	100
	2011	16.876	38.4	362	0.5	874	1.5	44877	100	19606	46.6	4863	11.6	208	0.5	42032	100
	2012	15.875	37.8	391	0.5	894	1.2	46876	100	20946	47.2	5391	12.1	202	0.5	44409	100
	2013	17.876	37.2	354	0.4	913	1.2	52987	100	22847	48.7	5639	12.7	204	0.4	46901	100
	2014	16.875	36.6	361	0.4	913	1.4	55766	100	25790	49.5	6425	12.2	249	0.5	52662	100
	2015	18.876	36.0	365	0.4	913	1.4	56112	100	26906	48.2	6769	12.1	231	0.4	55858	100
Group D	2016	21.876	35.4	391	0.3	913	1.4	56332	100	27154	48.3	7068	12.6	229	0.4	56219	100
	2017	23.765	34.8	389	0.3	913	2.2	56431	100	26947	48.1	6956	12.4	240	0.4	56133	100
	2018	23.743	37.3	356	0.7	913	2.3	63987	100	26984	45.8	7060	12.6	243	0.4	58894	100
	2019	23.931	35.2	354	0.8	913	1.9	54233	100	30110	47.6	7697	12.2	234	0.4	63262	100
	2020	24.543	35.8	361	0.7	1232	1.9	54322	100	32136	49.2	8508	12.8	252	0.4	65338	100
	2021	27.543	37.4	431	0.7	1872	2.3	63987	100	33368	49.1	8597	12.6	263	0.4	68023	100
	2022	28.871	39.8	432	0.8	2342	2.4	58986	100	34615	47.3	9060	12.4	262	0.4	73155	100
	2023	29.876	41.9	465	0.7	2431	2.6	63876	100	34651	46.2	9932	12.87	272	0.4	73165	100

References

1. Erdoğan, A., Güler, M.G.: Optimization and analysis of a hydrogen supply chain in terms of cost, CO2 emissions, and risk: the case of Turkey. Int. J. Hydrogen Energy. 48, 22752–22765 (2023). https://doi.org/https://doi.org/10.1016/j.ijhydene.2023.04.300
2. Caliskan, O., Sag, N.B., Ersoy, H.K.: Thermodynamic, environmental, and exergoeconomic analysis of multi-ejector expansion transcritical CO2 supermarket refrigeration cycles in different climate regions of Türkiye: Analyse thermodynamique, environnementale et exergoéconomique des cycles de ré. Int. J. Refrig. (2024). https://doi.org/https://doi.org/10.1016/j.ijrefrig.2024.05.006
3. Uzar, U., Eyuboglu, K.: Testing the asymmetric impacts of income inequality, financial development and human development on ecological footprint in Türkiye: A NARDL approach. J. Clean. Prod. 461, 142652 (2024). https://doi.org/https://doi.org/10.1016/j.jclepro.2024.142652
4. Isik, M., Sarica, K., Ari, I.: Driving forces of Turkey's transportation sector CO2 emissions: An LMDI approach. Transp. Policy. 97, 210–219 (2020). https://doi.org/https://doi.org/10.1016/j.tranpol.2020.07.006
5. March, A., Bennett, M., Germishuizen, M., Evans, T., Failler, P.: The status of Blue Economy development in Africa. Mar. Policy. 165, 106205 (2024). https://doi.org/https://doi.org/10.1016/j.marpol.2024.106205
6. Köprücü, Y., Acaroğlu, H.: How cereal yield is influenced by eco-environmental factors? ARDL and spectral causality analysis for Turkey. Clean. Environ. Syst. 10, 100128 (2023). https://doi.org/https://doi.org/10.1016/j.cesys.2023.100128
7. Abdallah, I., Udemba, E.N.B.T.-R.M. in S.S.: Industrial and agricultural progression amid pollution: A time series study of Turkey. Presented at the (2023)
8. Knorr, S., Weisener, C.G., Phillips, L.A.: Agricultural land management alters the biogeochemical cycling capacity of aquatic and sediment environments. Agric. Ecosyst. Environ. 357, 108661 (2023). https://doi.org/https://doi.org/10.1016/j.agee.2023.108661
9. Sharma, S., Wait, D.A., Khanal, C., Khanal, P.: Native bamboo Arundinaria gigantea restoration for climate smart land management: A review. Adv. Bamboo Sci. 10, 100125 (2025). https://doi.org/https://doi.org/10.1016/j.bamboo.2025.100125
10. Bowditch, E., Santopuoli, G., Neroj, B., Svetlik, J., Tominlson, M., Pohl, V., Avdagić, A., del Rio, M., Zlatanov, T., Maria, H., Jamnická, G., Serengil, Y., Sarginci, M., Brynleifsdóttir, S.J., Lesinki, J., Azevedo, J.C.: Application of climate-smart forestry – Forest manager response to the relevance of European definition and indicators. Trees, For. People. 9, 100313 (2022). https://doi.org/https://doi.org/10.1016/j.tfp.2022.100313
11. Naimoglu, M., Akal, M.: The relationship between energy technology, energy efficiency, renewable energy, and the environment in Türkiye. J. Clean. Prod. 418, 138144 (2023). https://doi.org/https://doi.org/10.1016/j.jclepro.2023.138144
12. Deng, P., Tan, X., Bai, Y., Li, H.: Forecasting chaotic behavior of the drill bit in real-time using a DNN model integrated with rock surface morphology. Mech. Syst. Signal Process. 221, 111710 (2024). https://doi.org/https://doi.org/10.1016/j.ymssp.2024.111710
13. Zul Azlan, Z.H., Junaini, S.N., Bolhassan, N.A., Wahi, R., Arip, M.A.: Harvesting a sustainable future: An overview of smart agriculture's role in social, economic, and environmental sustainability. J. Clean. Prod. 434, 140338 (2024). https://doi.org/https://doi.org/10.1016/j.jclepro.2023.140338

14. Büyüközkan, G., Uztürk, D.: Integrated design framework for smart agriculture: Bridging the gap between digitalization and sustainability. J. Clean. Prod. 449, 141572 (2024). https://doi.org/https://doi.org/10.1016/j.jclepro.2024.141572
15. Ağbulut, Ü.: Forecasting of transportation-related energy demand and CO2 emissions in Turkey with different machine learning algorithms. Sustain. Prod. Consum. 29, 141–157 (2022). https://doi.org/https://doi.org/10.1016/j.spc.2021.10.001
16. Yurtkuran, S.: The effect of agriculture, renewable energy production, and globalization on CO2 emissions in Turkey: A bootstrap ARDL approach. Renew. Energy. 171, 1236–1245 (2021). https://doi.org/https://doi.org/10.1016/j.renene.2021.03.009
17. Goldenberg, M.G., Burian, A., Seppelt, R., Santibañez Ossa, F.A., Bagnato, C.E., Satorre, E.H., Martini, G.D., Garibaldi, L.A.: Effects of natural habitat composition and configuration, environment and agricultural input on soybean and maize yields in Argentina. Agric. Ecosyst. Environ. 339, 108133 (2022). https://doi.org/https://doi.org/10.1016/j.agee.2022.108133
18. Isik, M., Ari, I., Sarica, K.: Challenges in the CO2 emissions of the Turkish power sector: Evidence from a two-level decomposition approach. Util. Policy. 70, 101227 (2021). https://doi.org/https://doi.org/10.1016/j.jup.2021.101227
19. Coruh, E., Bilgic, A., Cengiz, V., Urak, F.: Uncovering the determinants of bottom-up CO2 emissions among households in Türkiye: Analysis and policy recommendations. J. Clean. Prod. 469, 143197 (2024). https://doi.org/https://doi.org/10.1016/j.jclepro.2024.143197
20. Onenli, O., Ercan, H., Acikgoz, S., Kat, B.: Emission reduction pathways for satisfying Türkiye's future electricity demand: A cross-country dynamic panel analysis with linear programming scenarios. Clean. Energy Syst. 6, 100085 (2023). https://doi.org/https://doi.org/10.1016/j.cles.2023.100085
21. Karki, E., Sharma, A., Timsina, P., Chaudhary, A., Sharma, R., Brown, B.: Strategies to overcome stagnation in agricultural adoption despite awareness and interest: a case study of conservation agriculture in South Asia. Renew. Agric. Food Syst. 39, e14 (2024). https://doi.org/DOI: 10.1017/S1742170524000073
22. Alatawneh, A., Ghunaim, D.: Towards vehicle electrification: A mathematical prediction of battery electric vehicle ownership growth, the case of Turkey. Green Energy Intell. Transp. 100166 (2024). https://doi.org/https://doi.org/10.1016/j.geits.2024.100166
23. Tayyar, A.E., Gasim, N., Biçen, Ö.F., Mukhtarov, S.: An analysis of climate impacts on agriculture production: Evidence from Türkiye by BMA and A-ARDL approaches. J. Environ. Manage. 389, 126111 (2025). https://doi.org/https://doi.org/10.1016/j.jenvman.2025.126111
24. Temizceri, F.T., Kara, S.S.: Towards sustainable logistics in Turkey: A bi-objective approach to green intermodal freight transportation enhanced by machine learning. Res. Transp. Bus. Manag. 55, 101145 (2024). https://doi.org/https://doi.org/10.1016/j.rtbm.2024.101145
25. Adebayo, T.S., Agyekum, E.B., Kamel, S., Zawbaa, H.M., Altuntaş, M.: Drivers of environmental degradation in Turkey: Designing an SDG framework through advanced quantile approaches. Energy Reports. 8, 2008–2021 (2022). https://doi.org/https://doi.org/10.1016/j.egyr.2022.01.020
26. Guven, D., Kayalica, M.O.: Analysing the determinants of the Turkish household electricity consumption using gradient boosting regression tree. Energy Sustain. Dev. 77, 101312 (2023). https://doi.org/https://doi.org/10.1016/j.esd.2023.101312
27. Türker, Y.Ö., Aydin, A.: How ready is the Turkish Legislation for the green deal?

Energy Clim. Chang. 3, 100084 (2022). https://doi.org/https://doi.org/10.1016/j.egycc.2022.100084
28. Bakay, M.S., Ağbulut, Ü.: Electricity production based forecasting of greenhouse gas emissions in Turkey with deep learning, support vector machine and artificial neural network algorithms. J. Clean. Prod. 285, 125324 (2021). https://doi.org/https://doi.org/10.1016/j.jclepro.2020.125324
29. As, M., Bilir, T.: Enhancing energy efficiency and cost-effectiveness while reducing CO2 emissions in a hospital building. J. Build. Eng. 78, 107792 (2023). https://doi.org/https://doi.org/10.1016/j.jobe.2023.107792
30. Tao, W., Zhao, L., Wang, G., Liang, R.: Review of the internet of things communication technologies in smart agriculture and challenges. Comput. Electron. Agric. 189, 106352 (2021). https://doi.org/https://doi.org/10.1016/j.compag.2021.106352
31. Tuğaç, Ç.: Climate change adaptation: The missing component in the local climate change action plans of Turkish metropolitan municipalities. Urban Clim. 61, 102396 (2025). https://doi.org/https://doi.org/10.1016/j.uclim.2025.102396
32. Gungor, G., Sari, R.: Meta-analysis of the Turkish energy and climate pathways to achieve the net-zero emission target. Energy. 305, 132337 (2024). https://doi.org/https://doi.org/10.1016/j.energy.2024.132337
33. Yazar, M., Daloglu Cetinkaya, I., Baykal Fide, E., Haarstad, H.: Diffusion of global climate policy: National depoliticization, local repoliticization in Turkey. Glob. Environ. Chang. 81, 102699 (2023). https://doi.org/https://doi.org/10.1016/j.gloenvcha.2023.102699
34. Acar, S., Kat, B., Rogner, M., Saygin, D., Taranto, Y., Yeldan, A.E.: Transforming Türkiye’s power system: An assessment of economic, social, and external impacts of an energy transition by 2030. Clean. Energy Syst. 4, 100064 (2023). https://doi.org/https://doi.org/10.1016/j.cles.2023.100064
35. Gökçek, M., Paltrinieri, N., Liu, Y., Badia, E., Dokuz, A.Ş., Erdoğmuş, A., Urhan, B.B., Yoldaş, Ö.: Optimum sizing of hybrid renewable power systems for on-site hydrogen refuelling stations: Case studies from Türkiye and Spain. Int. J. Hydrogen Energy. 59, 715–729 (2024). https://doi.org/https://doi.org/10.1016/j.ijhydene.2024.02.068
36. Nayir, T., Uskun, E., Ilter, H., Ozkan, S., Kiraz, E.D.E.: Developing a climate change health literacy scale: A methodological study in Turkish adults. J. Clim. Chang. Heal. 23, 100451 (2025). https://doi.org/https://doi.org/10.1016/j.joclim.2025.100451
37. Raihan, A., Hasan, M.A., Voumik, L.C., Pattak, D.C., Akter, S., Ridwan, M.: Sustainability in Vietnam: Examining economic growth, energy, innovation, agriculture, and forests’ impact on CO2 emissions. World Dev. Sustain. 4, 100164 (2024). https://doi.org/https://doi.org/10.1016/j.wds.2024.100164
38. Zakaria, A., Azumah, S.B., Appiah-Twumasi, M., Dagunga, G.: Adoption of climate-smart agricultural practices among farm households in Ghana: The role of farmer participation in training programmes. Technol. Soc. 63, 101338 (2020). https://doi.org/https://doi.org/10.1016/j.techsoc.2020.101338
39. Boz, Y., Cay, T.: How smart and sustainable are the cities in Turkiye? - National policies and the enthusiasm level of the local governments. Heliyon. 10, e26002 (2024). https://doi.org/https://doi.org/10.1016/j.heliyon.2024.e26002
40. Bhatnagar, S., Chaudhary, R., Sharma, S., Janjhua, Y., Thakur, P., Sharma, P., Keprate, A.: Exploring the dynamics of climate-smart agricultural practices for sustainable resilience in a changing climate. Environ. Sustain. Indic. 24, 100535 (2024). https://doi.org/https://doi.org/10.1016/j.indic.2024.100535

41. Karakurt, I., Aydin, G.: Development of regression models to forecast the CO2 emissions from fossil fuels in the BRICS and MINT countries. Energy. 263, 125650 (2023). https://doi.org/https://doi.org/10.1016/j.energy.2022.125650
42. Cekinir, S., Ozgener, O., Ozgener, L.: Türkiye’s energy projection for 2050. Renew. Energy Focus. 43, 93–116 (2022). https://doi.org/https://doi.org/10.1016/j.ref.2022.09.003
43. Eslami, S., Noorollahi, Y., Marzband, M., Anvari-Moghaddam, A.: District heating planning with focus on solar energy and heat pump using GIS and the supervised learning method: Case study of Gaziantep, Turkey. Energy Convers. Manag. 269, 116131 (2022). https://doi.org/https://doi.org/10.1016/j.enconman.2022.116131
44. Destek, M.A.: Deindustrialization, reindustrialization and environmental degradation: Evidence from ecological footprint of Turkey. J. Clean. Prod. 296, 126612 (2021). https://doi.org/https://doi.org/10.1016/j.jclepro.2021.126612
45. Yilanci, V., Kilci, E.N.: A study on the macroeconomic and financial determinants of telecommunication infrastructure: Evidence from Turkiye. Technol. Soc. 77, 102573 (2024). https://doi.org/https://doi.org/10.1016/j.techsoc.2024.102573
46. Khan, R.: Beta decoupling relationship between CO2 emissions by GDP, energy consumption, electricity production, value-added industries, and population in China. PLoS One. 16, 1–22 (2021). https://doi.org/10.1371/journal.pone.0249444
47. Khan, R.: The impact of a new techno-nationalism era on eco-economic decoupling. Resour. Policy. 82, 103452 (2023). https://doi.org/https://doi.org/10.1016/j.resourpol.2023.103452
48. Khan, R.: Catch-up growth with alpha and beta decoupling and their relationships between CO2 emissions by GDP, population, energy production, and consumption. Heliyon. e31470 (2024). https://doi.org/https://doi.org/10.1016/j.heliyon.2024.e31470
49. Khan, R.: Comparing the effects of agricultural intensification on CO2 emissions and energy consumption in developing and developed countries. Front. Environ. Sci. https://doi.org/10.3389/fenvs.2022.1065634
50. Wang, C., Abbasi, K.R., Irfan, M., Ben-Salha, O., Bandyopadhyay, A.: Navigating sustainability in the US: A comprehensive analysis of green energy, eco-innovation, and economic policy uncertainty on sectoral CO2 emissions. Energy Reports. 11, 5286–5299 (2024). https://doi.org/https://doi.org/10.1016/j.egyr.2024.05.014
51. Khan, R.: The impact of agricultural intensification on carbon dioxide emissions and energy consumption: a comparative study of developing and developed nations.
52. Erdoğan, A., Geçici, E., Güler, M.G.: Design of a future hydrogen supply chain: A multi-objective model for Turkey. Int. J. Hydrogen Energy. 48, 11775–11789 (2023). https://doi.org/https://doi.org/10.1016/j.ijhydene.2022.12.071
53. Khan, S.Z.R.: Performance evaluation of e-commerce firms in China: Using three-stage data envelopment analysis and the Malmquist productivity index. PLoS One. 11, (2021). https://doi.org/10.1371/journal.pone.0255851
54. Rabnawaz Khan, Y.K.: Effects of Energy Consumption on GDP: New Evidence of 24 Countries on Their Natural Resources and Production of Electricity. Ekonomika. 99, 26–49 (2020). https://doi.org/https://doi.org/10.15388/Ekon.2020.1.2
55. Gürel, B., Kurtuluş, K., Yurdakul, S., Karaca Dolgun, G., Akman, R., Önür, M.E., Varol, M., Keçebaş, A., Gürbüz, H.: Combustion of chicken manure and Turkish lignite mixtures in a circulating fluidized bed. Renew. Sustain. Energy Rev. 189, 113960 (2024). https://doi.org/https://doi.org/10.1016/j.rser.2023.113960
56. Kınay, U., Laukkarinen, A., Vinha, J.: Renovation wave of the residential building stock targets for the carbon-neutral: Evaluation by Finland and Türkiye case studies for energy demand. Energy Sustain. Dev. 75, 1–24 (2023).

https://doi.org/https://doi.org/10.1016/j.esd.2023.04.014
57. Zambrano-Monserrate, M.A.: Clean energy production index and CO2 emissions in OECD countries. Sci. Total Environ. 907, 167852 (2024). https://doi.org/https://doi.org/10.1016/j.scitotenv.2023.167852
58. Atmaca, A., Atmaca, N.: Carbon footprint assessment of residential buildings, a review and a case study in Turkey. J. Clean. Prod. 340, 130691 (2022). https://doi.org/https://doi.org/10.1016/j.jclepro.2022.130691
59. Jonek-Kowalska, I.: Towards the reduction of CO2 emissions. Paths of pro-ecological transformation of energy mixes in European countries with an above-average share of coal in energy consumption. Resour. Policy. 77, 102701 (2022). https://doi.org/https://doi.org/10.1016/j.resourpol.2022.102701
60. Najmuddin, Omaid, Zhihui Li, Rabnawaz Khan, and W.Z.: Valuation of Land-Use/Land-Cover-Based Ecosystem Services in Afghanistan—An Assessment of the Past and Future. Land. 11, (2022)
61. Jebabli, I., Lahiani, A., Mefteh-Wali, S.: Quantile connectedness between CO2 emissions and economic growth in G7 countries. Resour. Policy. 81, 103348 (2023). https://doi.org/https://doi.org/10.1016/j.resourpol.2023.103348
62. Yin, C., Wang, C., Wang, Q., Ge, Y.: Effects of regional freight structure and energy intensity on CO2 emission of transport—a case study in Yangtze River Delta. Int. J. Sustain. Transp. 18, 379–392 (2024). https://doi.org/https://doi.org/10.1080/15568318.2023.2299918
63. Acaroğlu, H., Güllü, M.: Climate change caused by renewable and non-renewable energy consumption and economic growth: A time series ARDL analysis for Turkey. Renew. Energy. 193, 434–447 (2022). https://doi.org/https://doi.org/10.1016/j.renene.2022.04.138
64. Jahanger, A., Hossain, M.R., Awan, A.: Exploring the critical nexus among energy mineral, globalization, and CO2 emissions in NAFTA: What's the forum's response amid asymmetries? Resour. Policy. 90, 104825 (2024). https://doi.org/https://doi.org/10.1016/j.resourpol.2024.104825
65. Schilling, M.A., Esmundo, M.: Technology S-curves in renewable energy alternatives: Analysis and implications for industry and government. Energy Policy. 37, 1767–1781 (2009). https://doi.org/https://doi.org/10.1016/j.enpol.2009.01.004
66. Shahbaz, M., Patel, N., Du, A.M., Ahmad, S.: From black to green: Quantifying the impact of economic growth, resource management, and green technologies on CO2 emissions. J. Environ. Manage. 360, 121091 (2024). https://doi.org/https://doi.org/10.1016/j.jenvman.2024.121091
67. Sufyanullah, K., Ahmad, K.A., Sufyan Ali, M.A.: Does emission of carbon dioxide is impacted by urbanization? An empirical study of urbanization, energy consumption, economic growth and carbon emissions - Using ARDL bound testing approach. Energy Policy. 164, 112908 (2022). https://doi.org/https://doi.org/10.1016/j.enpol.2022.112908
68. ExxonMobil: Advancing climate solutions. (2022)
69. Lioudis, N.: What is the relationship between oil prices and inflation?, http://www.investopedia.com/ask/answers/06/oilpricesinflation.asp
70. Zhang, S., Zhang, X., Du, J., Wang, W., Pi, X.: Multi-target meridians classification based on the topological structure of anti-cancer phytochemicals using deep learning. J. Ethnopharmacol. 319, 117244 (2024). https://doi.org/https://doi.org/10.1016/j.jep.2023.117244
71. Ridoutt, B., Lehnert, S.A., Denman, S., Charmley, E., Kinley, R., Dominik, S.: Potential GHG emission benefits of Asparagopsis taxiformis feed supplement in

Australian beef cattle feedlots. J. Clean. Prod. 337, 130499 (2022). https://doi.org/https://doi.org/10.1016/j.jclepro.2022.130499
72. Sheraz, M., Deyi, X., Sinha, A., Mumtaz, M.Z., Fatima, N.: The dynamic nexus among financial development, renewable energy and carbon emissions: Moderating roles of globalization and institutional quality across BRI countries. J. Clean. Prod. 343, 130995 (2022). https://doi.org/https://doi.org/10.1016/j.jclepro.2022.130995
73. Li, D., Guan, X., Tang, T., Zhao, L., Tong, W., Wang, Z.: The clean energy development path and sustainable development of the ecological environment driven by big data for mining projects. J. Environ. Manage. 348, 119426 (2023). https://doi.org/https://doi.org/10.1016/j.jenvman.2023.119426
74. Rehfeldt, M., Fleiter, T., Herbst, A., Eidelloth, S.: Fuel switching as an option for medium-term emission reduction - A model-based analysis of reactions to price signals and regulatory action in German industry. Energy Policy. 147, 111889 (2020). https://doi.org/https://doi.org/10.1016/j.enpol.2020.111889
75. Deng, X., Du, L.: Estimating the environmental efficiency, productivity, and shadow price of carbon dioxide emissions for the Belt and Road Initiative countries. J. Clean. Prod. 277, 123808 (2020). https://doi.org/https://doi.org/10.1016/j.jclepro.2020.123808
76. Shukla, N., Shah, K., Rathore, D., Soni, K., Shah, J., Vora, H., Dave, H.: Androgen receptor: Structure, signaling, function and potential drug discovery biomarker in different breast cancer subtypes. Life Sci. 348, 122697 (2024). https://doi.org/https://doi.org/10.1016/j.lfs.2024.122697
77. Van Poecke, A., Tabari, H., Hellinckx, P.: Unveiling the backbone of the renewable energy forecasting process: Exploring direct and indirect methods and their applications. Energy Reports. 11, 544–557 (2024). https://doi.org/https://doi.org/10.1016/j.egyr.2023.12.031
78. Yang, X., Ramos-Meza, C.S., Shabbir, M.S., Ali, S.A., Jain, V.: The impact of renewable energy consumption, trade openness, CO2 emissions, income inequality, on economic growth. Energy Strateg. Rev. 44, 101003 (2022). https://doi.org/https://doi.org/10.1016/j.esr.2022.101003
79. Perone, G.: The relationship between renewable energy production and CO2 emissions in 27 OECD countries: A panel cointegration and Granger non-causality approach. J. Clean. Prod. 434, 139655 (2024). https://doi.org/https://doi.org/10.1016/j.jclepro.2023.139655
80. Yu, Y., Radulescu, M., Ifelunini, A.I., Ogwu, S.O., Onwe, J.C., Jahanger, A.: Achieving Carbon Neutrality Pledge through Clean Energy Transition: Linking the Role of Green Innovation and Environmental Policy in E7 Countries. Energies. 15, (2022). https://doi.org/10.3390/en15176456
81. Mendonça, A.K. de S., de Andrade Conradi Barni, G., Moro, M.F., Bornia, A.C., Kupek, E., Fernandes, L.: Hierarchical modeling of the 50 largest economies to verify the impact of GDP, population and renewable energy generation in CO2 emissions. Sustain. Prod. Consum. 22, 58–67 (2020). https://doi.org/https://doi.org/10.1016/j.spc.2020.02.001
82. Ozdemir, A.C.: Decomposition and decoupling analysis of carbon dioxide emissions in electricity generation by primary fossil fuels in Turkey. Energy. 273, 127264 (2023). https://doi.org/https://doi.org/10.1016/j.energy.2023.127264
83. Adebayo, T.S., Acheampong, A.O.: Modelling the globalization-CO2 emission nexus in Australia: evidence from quantile-on-quantile approach. Environ. Sci. Pollut. Res. 29, 9867–9882 (2022). https://doi.org/10.1007/s11356-021-16368-y
84. Grodzicki, T., Jankiewicz, M.: The impact of renewable energy and urbanization on

CO2 emissions in Europe – Spatio-temporal approach. Environ. Dev. 44, 100755 (2022). https://doi.org/https://doi.org/10.1016/j.envdev.2022.100755
85. Kwilinski, A., Lyulyov, O., Pimonenko, T.: Reducing transport sector CO2 emissions patterns: environmental technologies and renewable energy. J. Open Innov. Technol. Mark. Complex. 100217 (2024). https://doi.org/https://doi.org/10.1016/j.joitmc.2024.100217
86. Peiseler, L., Cabrera Serrenho, A.: How can current German and EU policies be improved to enhance the reduction of CO2 emissions of road transport? Revising policies on electric vehicles informed by stakeholder and technical assessments. Energy Policy. 168, 113124 (2022). https://doi.org/https://doi.org/10.1016/j.enpol.2022.113124
87. Kłosowski, G., Hoła, A., Rymarczyk, T., Mazurek, M., Niderla, K., Rzemieniak, M.: Use of the double-stage LSTM network in electrical tomography for 3D wall moisture imaging. Measurement. 213, 112741 (2023). https://doi.org/https://doi.org/10.1016/j.measurement.2023.112741
88. Llanos, C., Kristjanpoller, W., Michell, K., Minutolo, M.C.: Causal treatment effects in time series: CO2 emissions and energy consumption effect on GDP. Energy. 249, 123625 (2022). https://doi.org/https://doi.org/10.1016/j.energy.2022.123625
89. Meygoonpoury, E., Ghadim, M.K., Ziabakhsh-Ganji, Z.: Internationalization of renewable energy base businesses with a combined approach to networking and collaborative competition. Results Eng. 21, 101726 (2024). https://doi.org/https://doi.org/10.1016/j.rineng.2023.101726
90. Cevher, C., Feridun, G., Ebegil, M., Özkan, U., Coskun-Cevher, S.: Analysis of the effects of climate change on rangeland livestock: The example of the Central Anatolia Region in Türkiye. Small Rumin. Res. 245, 107464 (2025). https://doi.org/https://doi.org/10.1016/j.smallrumres.2025.107464
91. Ntawuruhunga, D., Ngowi, E.E., Mangi, H.O., Salanga, R.J., Shikuku, K.M.: Geospatial characterization of climate-smart agroforestry in two contrasting physiographic zones of Rwanda. Trees, For. People. 14, 100446 (2023). https://doi.org/https://doi.org/10.1016/j.tfp.2023.100446
92. Zaigham Abbas Naqvi, S.M., Hussain, S., Awais, M., Tahir, M.N., Saleem, S.R., Al-Yarimi, F.A.M., Ashurov, M., Saidani, O., Khan, M.I., Wu, J., Wei, Z., Hu, J.: Climate-resilient water management: Leveraging IoT and AI for sustainable agriculture. Egypt. Informatics J. 30, 100691 (2025). https://doi.org/https://doi.org/10.1016/j.eij.2025.100691
93. Avila, F.R. de, Barbosa, J.L.V.: Smart environments in digital agriculture: a systematic review and taxonomy. Comput. Electron. Agric. 236, 110393 (2025). https://doi.org/https://doi.org/10.1016/j.compag.2025.110393
94. Mdoe, C.N., Mahonge, C.P., Ngowi, E.E.: Mapping the trends, knowledge production, and practices of climate-smart aquaculture scholarship: Empirical insights from bibliometric analysis. Aquaculture. 598, 741939 (2025). https://doi.org/https://doi.org/10.1016/j.aquaculture.2024.741939
95. Kutlu Gündoğdu, F.: Analyzing critical barriers of smart energy city in Turkey based on two-dimensional uncertainty by hesitant z-fuzzy linguistic terms. Eng. Appl. Artif. Intell. 113, 104935 (2022). https://doi.org/https://doi.org/10.1016/j.engappai.2022.104935
96. Cui, Y., Khan, S.U., Deng, Y., Zhao, M.: Spatiotemporal heterogeneity, convergence and its impact factors: Perspective of carbon emission intensity and carbon emission per capita considering carbon sink effect. Environ. Impact Assess. Rev. 92, 106699 (2022). https://doi.org/https://doi.org/10.1016/j.eiar.2021.106699

97. Arora, T., Reddy, C.S., Sharma, R., Kilaparthi, S.D., Gupta, L.: Greenhouse gas emissions of Delhi, India: A trend analysis of sources and sinks for 2017–2021. Urban Clim. 51, 101634 (2023). https://doi.org/https://doi.org/10.1016/j.uclim.2023.101634
98. Khan, R., Zhuang, W., Najumddin, O., Butt, R.S., Ahmad, I., Al-Faryan, M.A.S.: The impact of agricultural intensification on carbon dioxide emissions and energy consumption: A comparative study of developing and developed nations. Front. Environ. Sci. 10, (2022). https://doi.org/10.3389/fenvs.2022.1036300
99. Shaikh, T.A., Rasool, T., Mir, W.A.: Fields of the future: Digital transformation in smart agriculture with large language models and generative AI. Comput. Stand. Interfaces. 94, 104005 (2025). https://doi.org/https://doi.org/10.1016/j.csi.2025.104005
100. Ahsan, T.M.A., Hassan, A.A., Thakur, A.K., Ahamed, M.S.: Transitioning from conventional systems to ground source heat pump for climate-smart greenhouse conditioning. Case Stud. Therm. Eng. 72, 106314 (2025). https://doi.org/https://doi.org/10.1016/j.csite.2025.106314
101. Morchid, A., Alblushi, I.G., Khalid, H.M., Alami, R. El, Said, Z., Qjidaa, H., Cuce, E., Muyeen, S.M., Jamil, M.O.: Fire detection and anti-fire system to enhance food security: A concept of smart agriculture systems-based IoT and embedded systems with machine-to-machine protocol. Sci. African. 27, e02559 (2025). https://doi.org/https://doi.org/10.1016/j.sciaf.2025.e02559
102. Ridzuan, N.H.A.M., Marwan, N.F., Khalid, N., Ali, M.H., Tseng, M.-L.: Effects of agriculture, renewable energy, and economic growth on carbon dioxide emissions: Evidence of the environmental Kuznets curve. Resour. Conserv. Recycl. 160, 104879 (2020). https://doi.org/https://doi.org/10.1016/j.resconrec.2020.104879
103. Christidis, P., Vetters, N., Ulpiani, G., Guardiola, M.O., Soler, J.R.L., Radics, M., Gonzalo, M.V.: Urban transport in the EU Mission on Climate Neutral and Smart Cities: preliminary data from applicants' expressions of interest. Transp. Res. Procedia. 72, 580–587 (2023). https://doi.org/https://doi.org/10.1016/j.trpro.2023.11.442
104. Chang, T., Hsu, C.-M., Chen, S.-T., Wang, M.-C., Wu, C.-F.: Revisiting economic growth and CO2 emissions nexus in Taiwan using a mixed-frequency VAR model. Econ. Anal. Policy. 79, 319–342 (2023). https://doi.org/https://doi.org/10.1016/j.eap.2023.05.022
105. Islam, M.J., Datta, R., Iqbal, A.: Actual rating calculation of the zoom cloud meetings app using user reviews on google play store with sentiment annotation of BERT and hybridization of RNN and LSTM. Expert Syst. Appl. 223, 119919 (2023). https://doi.org/https://doi.org/10.1016/j.eswa.2023.119919
106. Ben Jebli, M., Farhani, S., Guesmi, K.: Renewable energy, CO2 emissions and value added: Empirical evidence from countries with different income levels. Struct. Chang. Econ. Dyn. 53, 402–410 (2020). https://doi.org/https://doi.org/10.1016/j.strueco.2019.12.009

Exploring Climate Smart Agriculture in Turkey: Enhancing Food Security and Sustainable Practices for the Reduction of CO_2 Emissions

Wang Jie[1] Nawaz Khan[2]*

[1,2]*School of Internet Economics and Business, Fujian University of Technology, Fuzhou City, Fujian Province, China

Formatted: Superscript

*Corresponding Author:
Email: 61202008@fjut.edu.cn

Abstract

Climate-smart smart agriculture, which entails the reduction of CO_2 emissions, the adaptation and modification of technology to enhance resilience to climate change, and the sustainable increase of incomes. The aim of this study is to evaluate the effectiveness of smart agriculture practices in Turkey, which have a significant impact on food security and lower CO_2 emissions.To assess the efficacy of smart agriculture practices, which significantly enhance food security and reduce CO_2 emissions, this investigation implements the decoupling method. The decoupling process is responsible for estimating the percentage change in portfolio returns with high and low weighted risk over the projected five-year period from 1992 to 2023. This study employed the decoupling technique to estimate the portfolio returns and analyze their relationship with climate-smart agriculture and CO_2 emissions during the anticipated period of 1992 to 2023. The decoupling technique is responsible for estimating the percentage change in portfolio returns with high and low weighted risk. Furthermore, it has been shown that a percentage change in continuous, single aeration, and multiple aeration practices results in a change in climate-smart agriculture. Food security and sustainable practices contribute to fluctuations in CO_2 emissions levels, and this method also facilitates the forecast of the next five years. The results suggest that the decoupling trend in portfolio return is significantly influenced by factors such as rice cultivation, field rise, and soil management, which also contribute to the highest weighted risk. Additionally, this factor consistently shows the highest weighted importance in determining overall portfolio returns, as it also exhibits the largest marginal effects. Consequently, this investigation substantiates the detrimental influence of these variables on CO_2 emissions. Turkey's sustainable smart agriculture process is essential for the efficient expansion of the economy, as it integrates climate change considerations into current policies and initiatives and reinforces the policy indicator of economic consideration with environmental protection in terms of CO_2 emissions.

The estimated results of decoupling CO_2 emissions are subject to a 1% variation in rice cultivation, field fire, and managed soil. Rice cultivation and soil management are the factors that contribute to the highest weighted risk. The sustainable smart agriculture process in Turkey is crucial for the effective growth of the economy, as it integrates climate change considerations into existing policies and initiatives and supports the policy indicator of economic consideration with environmental protection in terms of CO_2 emissions.

The estimated results of decoupling CO_2 emissions are subject to a 1% variation in rice cultivation, field fire, and managed soil. Rice cultivation and soil management are the factors

Formatted: Highlight

that contribute to the highest weighted risk. The sustainable smart agriculture process in Turkey is crucial for the effective growth of the economy, as it integrates climate change considerations into existing policies and initiatives and supports the policy indicator of economic consideration with environmental protection in terms of CO_2 emissions.

Keywords: Climate Smart_ -Agriculture; portfolio return;return, environment; CO_2CO2 emissions; Turkey

1. Introduction

Climate-smart~~-smart~~ agriculture (CLS-A) reduces CO_2~~CO2~~ emissions by lowering the emission of CO_2~~CO2~~ from agricultural activities and by helping to sequester CO_2~~CO2~~ from the atmosphere [1]. Cover cropping practices,~~practices~~ which sequester and add CO_2~~CO2~~ into the year-end~~end~~ soil organic matter,~~matter~~ and enhanced grazing management,~~management~~ which increases carbon storage in CLS-A [2]. First, Turkey is very susceptible initially to environmental changes [3]. It is in the Mediterranean basin, where increases in temperature as well as droughts due to extreme weather are already happening [4, 5]. Furthermore, CLS-A helps to shape the economy~~economy~~ and uses sustainability in agriculture. As such, CLS-A practices resilience and buffers~~buffer~~ environmental change, making food security and a stable economy possible. Secondly, the huge role of GHG emissions (including CO_2 emission [6]. By implementing strategic CLS-A measures that will reduce emissions and support soil management and agroforestry, Turkey can significantly contribute to its efforts to meet and transition to a sustainable economy. ~~By taking CLS-A strategic measures that will reduce such emissions and support soil management and agroforestry, Turkey can make a big contribution to its effort to meet and transition to a greener economy.~~ In this case, the decoupling of CLS A development and rapid production from harmful processes of the environment is obtained. Correspondingly, it provides a framework for Turkey to modernize and reinforce its CLS-A sector and thereby benefit the environment~~environment~~ and economy [7, 8].

The CLS-A is a holistic approach designed to transform agricultural systems to effectively and sustainably support food security and development under a changing climate. It integrates three main pillars: increased productivity, enhanced resilience, and reduced GHG emissions. Turkey, in a region highly vulnerable to climate change impacts, is increasingly focusing on CLS-A to address its agricultural challenges, ensure food security, and contribute to global climate goals. Turkey's agriculture sector is a vital source of economy and employment, but it faces some significant challenges regarding soil degradation, food security concerns, and GHG emissions. In case of soil degradation, threateningthe long-term sustainability [9]. The food security of agriculture has created an important role in reliance on imports and higher food prices. In 2023, the food inflation rates were over 55%, and the agriculture sector contributed 71.5 Mt CO_2 equivalent in 2023, which is an increase of 37.9% from 1990 [10]. ~~I examine th e CLS-A consequence in environmental protection by way of agroforestry — the integration of trees into agricultural systems, achieving twin goals: limiting forest denudation and sequestering atmospheric CO2 in biomass and soil~~We examine the environmental protection implications of CLS-A through agriculture, which involves the integration of trees into agricultural systems to achieve two objectives: the limitation of forest denudation and the sequestration of atmospheric CO_2 in biomass and soil [11, 12]. The CLS-A is also approaching productivity and income regarding agriculture, in which sustainable intensification and innovation systems play the most important role in climate, where climate-smart technology and practice control environmental degradation in the public and private sectors and help farmers to facilitate technology transfer and knowledge sharing [13]. Second, enhanced resilience shows that conservation agriculture and diversification of farming systems regarding the crops and livestock reduce the risk associated with climate impacts [14]. Third, mitigation is like reducing emissions by livestock, soil management, and land use management. Lastly, Turkey can move forward with the technological revolution in agriculture, and also the policy development and innovative financing mechanism will be crucial for the widespread adoption of CLS-A ~~Environmental policies related to CO2 emission applied to Turkey's agricultural sector have been studied~~ [15].

Formatted: Indent: First line: 0.5"

Formatted: Font: Times New Roman, 12 pt

Formatted: Indent: First line: 0.5", Space Before: 0 pt, After: 0 pt

Formatted: Font: Times New Roman, 12 pt

Formatted: Font: Times New Roman, 12 pt

Formatted: Font: Times New Roman, 12 pt

The two CLS-A practice primary objectives are climate change mitigation and its sustainability. [12, 13]. The CLS-A practice has two primary objectives:objectives, to minimize climate change and then maintain the sustainability of this aim [16, 17]. The other was the sustainable agriculture practice focusing on production methods thatwhich can diminish CO_2CO2 emissions [17–19]. Moreover, the two main contributions demonstrated, by means of the application of decoupling techniques, the important contribution to CO_2CO2 emissions as well as the vulnerability of climate change [20, 21]. This research argues that by decoupling, Turkey can meet its food security goals with limited environmentalenvironment impacts [22]. To investigate and implement effective decoupling techniques, we need a multi-pronged prong approach that considers such technological developments [23]. It may be possible to decouple these goals: byBy increasing food production, we can stop our environmental impact [24]. There are many ways to do this. Second, agricultural practices such as enhanced agricultural practices,practices including integrated pest management, conservation tillage,tillage and precision CLS-A, have been tested to increase the crop yield while reducing water consumption, soil erosion,erosion and dependence on pesticidesof pesticides, [25]. Second, eco-friendly friendly innovations: water for irrigation can be sourced from salt water, drought-resistant resistant crops are grown,grown and a vertical farming approach is adopted,adopted which will technically work around the constraints of available resources and lead to more efficient food production.

Third, a significant amount of food is wasted due to issues inthere is a lot of wasted food because of the supply chain. Reducing food waste from farm to fork is one approach that can helpaid with decoupling. Lastly, a significant portion of the food waste occurs throughout the supply chain. The results of this study suggest that the decoupling (portfolio returns) of the agricultural sector in Turkey could have a substantial influence on the reduction of CO_2 emissions and the improvement of food security. Decoupling direct payments in Turkey's agricultural sector may significantly contribute to enhancing food security and reducing the sector's CO_2 emissions impact, according to the findings of this study. Decoupling can foster multifarious, sustainable, and fruitful agricultural methods by empowering producers to make decisions more in line with market forces. Turkey would benefit from implementing supplementary policies that encourage producers to cultivate crops using sustainable methods, thereby slowing down the process. Transitioning to a decoupled technique could be a substantial enhancement for Turkey in addressing the challenges of food security and high levels of CO_2 emissionsTransitioning to a decoupled direct payment system could be a significant improvement for Turkey in addressing the challenges of food security and high levels of CO_2 emissions [26]. This CLS-A research aims to improve low-cost, long-term farming systems. It could involve finding new sustainable food buyers or verifying fair trade [27]. Rural education, healthcare, and infrastructure are more accessible with decoupling. A step toward decoupling is the implementation of waste-reduction techniques all the way from the farm to the table. These are things Turkey can do to improve the world and secure its food supply for the future [24, 25].As an outcome of decoupling, rural infrastructure, healthcare, and education are more readily accessible. Significant progress toward decoupling has been achieved through the implementation of waste-reduction techniques. By implementing these measures, Turkey can guarantee its food supply in the future and improve the global environment [28, 29].

This study emphasizes CLS-A's climate change adaptation through research. It underlines the need to focus on relevant research areas like drought management, climate change effects, adaptation, and evidence-based food security. It also emphasizes the need to collect and verify climate change data in each river basin and region. Authorities can affect future event preparations after accounting for the data. Research and development aims to improve land, water, and crop management in response to climate change. Thus, the literature

Formatted: Font: Times New Roman, 12 pt, Font color: Auto, English (United States)

Formatted: Font: Times New Roman, 12 pt, Font color: Auto, English (United States)

Formatted: Font: Times New Roman, 12 pt, Font color: Auto

emphasizes the following ideas and actions needed to reach this goal. This study emphasizes the need to study how climate change may affect CLS-A output, production, and local information. The main goal is to monitor climate change and create measures to limit its effects on agriculture. This study examines whether sustainable farming might reduce climate change's impact on Turkish food production. We investigate Turkey's environmental and economic policies using critical economic facts, global choices, and laws. According to studies, Turkish farmers can slow global warming by using modern farming methods. The legal application of Turkey's program reduces hazardous emissions, boosting economic growth and environmental security. Economic data can illustrate how financially feasible ideas are.

This study has two sections: background information and research on decoupling's environmental implications. Section 3 describes the research goals, theoretical and conceptual frameworks, and methodology and results. Estimation and results are in Section 4. Section 5 addresses results. The conclusion summarizes the investigation's findings and suggests relevantconcludes the investigation and suggests policies.

2. Literature Review

2.1. Climate Smart Agriculture

A number of sustainable CLS-A-related policies and projects have been established by the Turkish government [30, 31]. However, additional efforts are necessary to ensure the effectiveness of these policies and initiatives.To make these policies and initiatives work, though, more work is required. ToIn order to create new sustainable agricultural technology, the agricultural sector must also engage in research and development. How Turkey deals with its CO_2 emissions problems will determine the fate of its CLS-A economy [32]. In addition, the Turkish Ministry of Agriculture and Forestry (TMAF) has stated that the objective is to enhance the sustainability of agricultural development and the competitiveness of agricultural food security. The CLS-A has three most critical components, which provide a comprehensive global perspective: productivity with food security and income, the adoption and development of resilience to environmental change, and the reduction of CO_2 emissions, as clearly identified by a framework [33]. As long as the solutions are within reasonable costs, individuals who are responsible for molding policies and farmers can identify the methods that effectively capture the most CO_2 emissions [34]. It is imperative to investigate the factors that influence individuals' decisions to adopt ecological and dining behaviors. [35]. More sustainable methods can help Turkey's CLS-A endure the impacts of climate change, water scarcity, and soil degradation [36]. Consequently, this sector will persist in the future, guaranteeing that the Turkish populace has an adequate supply of sustenance [37]. The sustainable growth also supports the transition to transformative change in smart agriculture through a variety of initiatives, such as innovation, capacity development, and the technological revolution in the context of smart farming. More sustainable methods can help Turkey's CLS-A endure the impacts of climate change, water scarcity, and soil degradation. Consequently, this sector will persist in the future, guaranteeing that the Turkish populace has an adequate supply of sustenance.

Currently, there is insufficient information available to determine whether various decoupling strategies are the most effective in mitigating the impact of climate change on CLS-A. Furthermore, it is imperative to conduct additional research toin order to determine the most cost-effective decoupling solutions [38]. As long as the solutions are within reasonable costs, individuals who are responsible for molding policies and farmers can identify the methods that effectively capture the most CO_2 emissions [29]. It is imperative to investigate the factors that influence individuals' decisions to adopt ecological and dining behaviors. [30]. Research is necessary to develop effective laws that promote environmental protection, prevent detrimental actions, and ensure equal opportunity in agriculture [39, 40]. Additionally, research is

Formatted: Indent: First line: 0.5"

necessary to investigate the consequences of policies that are currently in effect and planned for the future [26].

2.2. Sustainable practice of Turkey and CO_2~~CO_2~~ emissions

Turkey can address these challenges and fill these knowledge gaps by implementing a successful decoupling strategy that is financially feasible, socially acceptable, and sustainable. It guarantees the availability of food for individuals and guarantees the sustainability of food systems in the future. In spite of the adoption of CLS-A, there are still certain research areas in it~~CLS-A~~ that require improvement to achieve a more favorable outcome and acceptance. Currently, there are numerous critical regions in the globe [41]. It is imperative that we determine whether CLS-A is economically feasible for small and medium-sized farms in Turkey [42]. ~~The implementation of a program that assists farmers in enhancing their digital literacy is necessary to enable them to implement sustainable practices that leverage new technologies two successful policy instruments that can be used to encourage farmers to adopt sustainable practices [39] are subsidies and tax breaks, which are informed by research [40].~~ To facilitate the successful implementation of sustainable practices that capitalize on emerging technologies, it is imperative that farmers receive assistance in improving their digital literacy [43]. Subsidies and tax breaks, which are informed by research, are two policies that have been effective in motivating farmers to embrace sustainable practices [44]. In order to incentivize producers to embrace market mechanisms, sustainable practices, such as certification systems or premium pricing for sustainably produced food,~~food~~ could be investigated. Filling these knowledge shortfalls in agricultural sustainability can hasten Turkey's shift to ecologically friendly farming practices [45].

3. Research Methodology

3.1. The CLS-A Method and Decoupling

A dynamic development model of Turkey's global economy was employed in this segment to examine the interplay between food security and the environment. In this section, we analyzed decoupling as a potential solution to improve food security and reduce CO_2 emissions in Turkey. In the context of agriculture and CO_2 emissions in the environmental factors, the fundamental approach of this study is to decouple the determinants of portfolio return. First, the portfolio technique considers environmental factors and agricultural shocks as random market risks, thereby isolating their impact. We directly attribute the climate events—droughts, food, consumer preferences, agriculture policies, and sustainable agriculture products in this portfolio—through the decoupling procedure. Consequently, this method is more detailed in its ability to comprehend the fundamental portfolio change in the context of high- and low-weighted risks in numerous, continuously inundated, and single aeration scenarios. Secondly, this technique also enables us to gain a more comprehensive understanding of the environmental degradation caused by CO_2 emissions in the agricultural system. Specifically, we can directly distinguish the performance of sustainable farming practices that are influenced by commodity market prices. Thirdly, we examine the investment strategies within the agricultural sector. Develop more targeted risk mitigation strategies for environmental and agricultural vulnerabilities through portfolio returns. In this regard, it is straightforward to distinguish between the financial performance that is influenced by the market's fluctuating prices and that that is influenced by the improvement of agricultural practices that provide the most optimal balance between economic viability and environmental sustainability. This can be achieved by reducing CO_2 emissions for a given level of economic return, or vice versa. Additionally, this method provides an informative tool for the

Formatted: Subscript

Formatted: Font color: Black

Formatted: Font: Times New Roman, 12 pt

Formatted: Font: Times New Roman, 12 pt

Formatted: Font: Times New Roman, 12 pt

Formatted: Font: Times New Roman, 12 pt

Formatted: Font: Times New Roman, 12 pt

Formatted: Font: Times New Roman, 12 pt

Formatted: Font: Times New Roman, 12 pt

Formatted: Font: Times New Roman, 12 pt

Formatted: Font: Times New Roman, 12 pt, Subscript

Formatted: Font: Times New Roman, 12 pt

Formatted: Font: Times New Roman, 12 pt

Formatted: Font: Times New Roman, 12 pt

Formatted: Font: Times New Roman, 12 pt

determination of environmental policies, agriculture trade, resilience, and allocation. Furthermore, it has been consistent with sustainable development and market conditions, which has facilitated the development of a portfolio decoupling technique that is designed to reconcile environmental resilience with financial performance.

We have made some modifications to the decoupling technique and have discussed them in the context of decoupling methods for controlling energy output and ingestion. This work is a continuation of previous studies [46–48]. The economic exercise is not instantaneously disregarded on the basis of decoupling, as the previous research investigation on decoupling strategies [46] reveals a one percent change in each year and their predicted change regarding decoupling. Given that the robustness and relationship have already been established through decoupling and subsequent portfolio turnover, which is a modification of research methodologies based on the decoupling methodology. The portfolio return has been estimated following the computing decoupling, and it is possible to predict the next five years and determine a one percent shift in CO_2 emissions using other indicators.

This text begins with an examination of decoupling, followed by an estimation ofWhat follows is an examination of decoupling, and after that we go on to estimating portfolio return using weighted risk and return. Finally, we will discuss the fluctuation of this return. [47]. The negative portfolio return value represents the separated decoupling values. Consequently, for each category, the returns of the portfolio can be examined for a complete eight years [46]. The last step is to examine the variation in portfolio returns for every group. The next four steps make up the computation. At the onset, we make use of beta decoupling toin order to ascertain the average transformation of the environment as well as the percentage change for each group individually. This implied the elastic range decoupling condition. Initially, indicators are evaluated by multiplying the covariance and variance by 8/7 and 1%, respectively. Next, portfolio returns are calculated with high and low weighted risk by decoupling positive and negative attitudes. The weighted risk indicated that the total risk and average return were contained in the weighted portfolio return. Consequently, the highest and lowest attitudes of decoupling in this weighted risk model result in a 1% change in CO_2 emissions. This 1% change in decoupling has caused a 1% shift in the following areas: rice cultivation, enteric fermentation, field fire, managed soil, urea application, and manure management. The second step involves determining the average return and overall risk for each cohort after decoupling (covariance/variance multiply with 8/7 and 1%, respectively)The 1% variation in decoupling has caused a 1% shift in the following areas: rice cultivation, enteric fermentation, field fire, managed soil, urea application, and manure management. Second, upon decoupling, we calculate the overall risk and average return for each individual group [49, 50].

. The 1% variation in decoupling has caused a 1% shift in the following areas: rice cultivation, enteric fermentation, field fire, managed soil, urea application, and manure management. Second, upon decoupling, we calculate the overall risk and average return for each individual group [45, 46].

The mean return offers details aboutprovides insight into the conventional assessment, while the total risk indicates the standard deviation for each group over a period of eight years. Furthermore, the portfolio return with weighted risk is computed by adding the 100% risk premium to the adjusted return indicators of reduced environment, namely field fire, managed soil, urea application, manure management, enteric fermentation, and rice cultivation, subsequent to the average return and total risk [51, 52]. A 1 percent change in the environment is then computed using the projected weighted return and risk of each cohort. In Section 3, the effects of the environment and the impact of a 1% change in food security on CO_2 emissions degradation are also evaluated individually. Moreover, the decoupling shift at different junctures is ascertained by the groups [53]. For instance, a 1% shift in environment decoupling can be attributed to modifications in CLS-A, which encompass enteric in the field burning,

managed soil, urea application, manure management, enteric fermentation, and rice cultivation [54].

3.2. Sources of the Dataset

~~From 1992 to 2023, this study examines the CO_2 emissions impacts of several agricultural practices in Turkey, including field burning, managed soil, urea application, manure management, enteric fermentation, and rice farming.~~

Formatted: Indent: First line: 0"

From 1992 to 2023, this study examines the CO_2 emissions implications of different agricultural techniques in Turkey, such as field burning, managed soil, urea application, manure management, enteric fermentation, and rice production, with the assistance of decoupling techniques. It then independently evaluates the portfolio returns using high and low weighted risk. The decoupling operation divides into four distinct sections (A–D) for the purpose of analysis. All of the subsets cover a full five years. Our Data in the World and the World Bank (WB) both have datasets that we analyzed using panel data (Appendix A). Inspired by eco-economic separation and a byproduct of techno-nationalism, accessibility is a determining element in the metrics' selection. The primary objective of this research is to assess the impact of CLS-A on the decoupling of CO_2 emissions in a statistically valid sample of Turkey. The significant increase in urea application in 2020 could be due to a combination of factors. These might include new government policies or subsidies that incentivized greater fertilizer use, an exceptionally favorable agricultural season that demanded more nutrients, or economic shifts such as a decrease in urea prices or an increase in crop prices making higher application more profitable. (Appendix B).

This increase is mostly attributable to the country's heavy use of coal to meet its growing energy demands. We investigate how these nations' actions affect decoupling on a global scale [55]. Based on their ecological situation and agricultural progress, we have ranked the nations [56]. Because of the association between reliance on these resources and volatility, it is widely believed that the price environment for commodities derived from them threatens the nation's purpose of sustainable economic growth. We determine that changes in the response variables are due to a decoupling estimate ranging from +1 to -2, which is a surrogate for measuring the effectiveness of agricultural initiatives to reduce CO_2~~CO_2~~ emissions harm [57]. The decoupling concept is further explained by looking at the performance of the portfolio. Conversely, if the decoupling analysis turns out to be unfavorable, we use techno-nationalism to rein in the CO_2 emissions decoupling effect and come up with workable solutions for the agricultural sector's transformation. The decoupling method was used to conduct a weighted calculation [58]. It demonstrates that as environmental conditions change by one percentage point, field burning, managed soil, urea application, manure management, enteric fermentation, and rice cultivation all change proportionately. The weighted values with a flexible range became readily apparent when distinct attitudes were uncoupled [59].

3.3. Portfolio Returns

~~3.3. Portfolio Returns~~

Formatted: Afar (Ethiopia)

Formatted: Heading 3

Formatted: Indent: First line: 0"

Formatted: Space Before: 0 pt, After: 0 pt

The efficiency of six key CLS-A indicators—managed soil, field burning, urea application, enteric fermentation, manure management, and rice cultivation—is examined in this study. We calculate the portfolio return (average return and total risk) for each of them

under three different aeration regimes. Through four distinct categories: continuously flooded, single aeration, and multiple aeration in groups (A to D). Thus, we use the current scientific knowledge of these activities to interpret the predicted effect on the environment by CO_2 emissions, which is drawing a connection between these indicators. Furthermore, the portfolio returns indicate things like yield, operational risk, profitability, weather-related hazards, and input cost variations. This is different from financial market indicators like decoupling or risk premiums, which show how investors perceive the risk and return in the capital markets as a whole. A new dataset that includes these financial metrics or a theoretical framework that explicitly links the market-level financial performance of agricultural ventures to their greenhouse gas emissions would be needed to set up a direct, empirically observed causal relationship between these particular financial market indicators and CO_2 emissions. For the first time, we have the data needed to assess the potential environmental impacts, risks, and economic feasibility of individual agricultural practices on individual farms. But when looking at the economy and finance as a whole, it is possible to speculate about a correlation between financial indicators and CO_2 emissions; this is especially true when it comes to the explicit pricing of climate change risks on the market.

The CLS-A indicator's estimated weighted return value in the portfolio reveals how much of an impact the better decoupling approach has on the return. Two outcomes could occur. The first is the disparity between the variable's Turkish investment breadths. The indicators over the next eight years are a further factor to considerthink about. The weighted risk and possible return are determined using the consequences of the environment, with the shared aid of decoupling. There are three steps to the calculation process [60]. The average return and risk value variable is one technical evaluation statistic that differentiates the four countries' economies (A–D) [48]. The average return is calculated in this research using total risk and average beta decoupling. That is consistent with the CO_2 emissions standard deviation for the past eight years. Secondly, we determined the portfolio weight by using the relative relevance of the three performance measures (agricultural) to assign a value to each. We allot a certain percentage of the portfolio to each variable according to criteria like CO_2 emissions impact and economic development. The third phase relies on the concept of weighted rewards and risk [61]. –This portfolio's enormous potential gains and losses are shown by the big swings. Additionally, the disparity between risk and weighted return is brought to light. To reduce CO_2 emissions risks to manageable levels, it is necessary to evaluate the viewpoints of the most risk-tolerant individuals, which can be achieved through changes in technology,technology changes in CLS-A,CLS-A and strategic policies [62]. With this data, we were able to compute the average return and total risk, which we then adjusted using weighted percent. For the most part, previous studies have used a stochastic approach to explaining the portfolio selection problem, treating asset returns as random variables, and have assumed that the statistical feature of agricultural stock returns will remain constant [63]. Although decoupling can be advantageous,helpful, modeling portfolio selection under weighted return is problematic owing to the dynamic environment in different regions. In addition, multiply the total risk, average return, and estimated weighted value of each category by their corresponding values [64]. We calculate the weighted return value by averaging itthe return value. The risk value is an inevitable starting point for the weighted risk value. In this case, we estimate the total risk and average return as we approach the beta decoupling levels. It was able to accomplish this by taking a more solid and sincere approach to the percentage change in agricultural technology [63, 65].

Formatted: Font color: Black, Afar (Ethiopia)

Pollutants released into the atmosphere by fertilizer applications, manure management, and other agricultural inputs are all part of the agricultural inputs that contribute to the degradation of naturally occurring soils. Industrial waste products, such as crop stubble and straw, are associated with emissions from field burning [66]. One major contributor to GHG

emissions is the widespread use of urea fertilizer in CLS-A production. Among the numerous agricultural sources, such as manure management, field burning, urea application, enteric fermentation, and agricultural soils, among many others, urea—a nitrogen-based fertilizer—can release nitrous oxide, a potent CLS-A. All emissions from agricultural sources add up to total CO_2 emissions. [67–70]. The findings unequivocally demonstrate that emissions in each of the four categories have exhibited~~demonstrated~~ a progressively increasing trend over the course of the years. The emissions from managed soils have registered the most significant increase, which is most likely attributable to the increased application of fertilizers and manure to~~in order to~~ improve crop yields [71]. In addition, the data clearly demonstrate that CLS-A plays a crucial part in contributing to Turkey's overall CO_2 emissions profile. In the year 2020, the agriculture sector was responsible for around twenty percent of the total emissions that were produced by the nation. The information presented in Appendix A provides a rundown of the CO_2~~CO_2~~ emissions that will be produced by Turkish CLS-A in the year 2020 [72]. One of the most significant contributors to carbon dioxide emissions within Turkish CLS-A is enteric fermentation, which accounts for approximately forty percent of the overall emissions [73]. The second-biggest source of carbon dioxide emissions from Turkish CLS-A, after manure management, accounts for about 25% of the overall emissions [74]. Animal manure has the potential to release two powerful greenhouse gases—methane and nitrous oxide—during the storage, processing, and application phases of manure management. Furthermore, for Turkey's CO_2 emissions, CLS-A soils account for more than 15% of the overall total. Agricultural soil organic matter cultivation and nitrogen fertilizer use both contribute to the formation of nitrous oxide, the gas responsible for these emissions [75].

3.4. Portfolio Equations

The weighted portfolio return was calculated for each agriculture management practice and strategy in various groups under four varying aeration conditions in terms of weighted risk (total risk) and weighted return (average return) in the portfolio return. Initially, weighted risk suggests a higher degree of unpredictability in the high- and low-risk zones. Similarly, the environmental impact and economic outcomes are subject to variation. In the event of economic outcomes, the potential for eccentricities from the average return is unstable yield and volatile profits. Additionally, the environmental impact—CO_2 emissions—is highly inconsistent across the various numbers of years in groups, which poses a high risk. Secondly, the agricultural strategies' weighted return outcomes are linked to both economic and environmental advantages. For example, a 1% modification in strategies results in an increase in profit, which in turn increases the return. The environmental strategies in terms of CO_2 emissions indicated that an average reduction and contraction in the environment are represented by a change of 1% in predictor variables. In the agricultural system, the average condition, such as continuous flooding and single and multiple aerations, is relevant in predictor variables that substantially influence CO_2 emissions. In the context of portfolio return, the most frequently used formulations for weighted average return and risk are as follows:

The weighted average return is denoted by $R_{i,j}$ (CO2 emissions) of agriculture strategy i under the condition of aeration, which is specified with i over n observed periods (Groups A-D) in Eq. 1. The weighted return specify with continuous flooding for managed soil, field fire, urea application, enteric fermentation, manure management, and rice cultivation is calculated as:

$$Weighted\ Average\ return_{i,j} = \frac{1}{n}\sum_{k=1}^{n} \mathrm{R}_{i,j,k} \qquad \text{Eq.1}$$

Eq. 2 illustrates the dispersion of individual outcomes in relation to the weighted average return, as denoted by

Formatted: Indent: First line: 0"
Formatted: Font: Times New Roman, 12 pt
Formatted: Font: Times New Roman, 12 pt
Formatted: Font: Times New Roman, 12 pt
Formatted: Font: Times New Roman, 12 pt
Formatted: Font: Times New Roman, 12 pt
Formatted: Font: Times New Roman, 12 pt
Formatted: Font: Times New Roman, 12 pt
Formatted: Font: Times New Roman, 12 pt
Formatted: Font: Times New Roman, 12 pt
Formatted: Font: Times New Roman, 12 pt

$$Weighted\ risk = \frac{1}{n\sum_{k=1}^{n}(\mathrm{R}_{i,j,k} - Weighted\ Average\ return_{i,j})^2} \qquad \text{Eq.2}$$

The most prevalent portfolio return formulations, which are calculated on the basis of decoupling, are the weighted average and weighted risk. The weighted risk in Eq.2 is typically represented by the σ of the return $\mathrm{R}_{i,j,k}$, which is a measure of the dispersion of individual outcomes around the $Weighted\ Average\ return_{i,j}$. The agricultural strategy outcomes are displayed with the same value as i under the aeration condition j during a specific period “k”, and the total number of periods is represented by “n”. The individual effects of the individual predictor variables, such as managed soil, field burning, urea application, enteric fermentation, manure management, and rice cultivation, on the portfolio return have been demonstrated in terms of continuous, single, and multiple aeration.

Formatted: Font: Times New Roman, 12 pt

Formatted: Font: Times New Roman, 12 pt

Formatted: Font: Times New Roman, 12 pt

Formatted: Font: Times New Roman, 12 pt

Formatted: Font: Times New Roman, 12 pt

Formatted: Font: Times New Roman, 12 pt

Formatted: Font: Times New Roman, 12 pt

4. Results

We used data for Turkey's nation from 1992 to 2023 and the portfolio return concept to look at how a~~Turkey nations from 1992 to 2023 and the portfolio return concept to look at how~~ resurgence with repeated and single aeration and continuous flooding affected high- and low-risk zones. The portfolio return is computed on the basis of the decoupling technique. Specifically, it is demonstrated that a 1 percent change in multiple and single aeration and continuous flooding leads to a 1 percent change in CLS-A. In Fig. 1, we can observe the pattern of wheat yields in Turkey throughout the four categories (A-D). The formatting condition for all CLS-A figures in this portfolio is evident in the returns, which feature three marks: a checkmark, an exclamation mark, and a heavy cross mark. The exclamation mark is recorded when the result is greater than 70%, and a hefty cross is indicated when the result is greater than 30%. The checkmark indicates a decoupling attitude of less than 70%. ~~In this portfolio, returns for all CLS-A figures show the formatting condition with three marks, like a checkmark, an exclamation mark, and a heavy cross mark. The checkmark shows the less than 70% decoupling attitude when the result is more than 70%, so it records an exclamation mark, and when the result is more than 30%, it indicates heavy cross.~~ Furthermore, the marks indicate issues related to field burning, managed soil, and urban development within the~~marks show that field burning, managed soil and urban development in terms of~~ CLS-A sectors [76, 77]. The increase from 2.4 metric tons/ha in 2013–14 to 2.5 metric tons/ha in 2022–23 indicates~~From 2.4 metric tons/ha in 2013–14 to 2.5 metric tons/ha in 2022–23, it showed~~ that wheat yields have been rising consistently. We expect wheat yields to rise even further in 2023–24, reaching 2.7 tons per hectare. Fig. 1 reveals that Turkey's wheat area remained virtually unchanged at 7,020,000 hectares (ha) between 2015/16 and 2019/20, compared to 7,860,000 ha in 2016/17. However, the wheat harvest has been more unpredictable, fluctuating between 15,000 metric tons in 2014–15 and 21,000 metric tons in 2017–18. The projected wheat harvest for 2023–24 is 19,500 million metric tons.

4.1 High-Risk Zone of CLS-A

According to Fig. 1, the portfolio's CLS-A needs are~~portfolio CLS-A need is~~ assessed for multiple and single aeration and continuously flooded separately in high- and low-risk zones. Firstly, we analyzed nations with a high-risk factor. In our assessment, along with each country's efforts to combat climate change, we discovered that new technical regulations impact the CLS-A separation characterized by three separate structural marks.

Similarly, in Fig. 1, portfolio-weighted results of more than 70% are shown by the exclamation mark in high- and low-risk countries, respectively. The portfolio shows the formatting condition with a checkbox, exclamation point, and heavy cross mark. Checkmarks indicate less than 70% decoupling, exclamation marks more than 70%, and heavy crosses more than 30%. In terms of total risk and average return in weighted portfolio return, the largest decoupling attitude is reported in Turkey (0.248% and 1.249%) and in field burning (0.567% and 0.297%) in groups D and C with indicated heavy crosses, respectively.

Formatted: Indent: First line: 0.5"

			Managed Soil	Field burining	Urea application	Enteric fermentation	Manure management	Rice cultivation
Continously Flooded	Av.return	Group A	✔ 0.164	✖ -0.511	! -0.190	✔ 0.130	! -0.090	✖ -0.105
		Group B	✖ -0.197	✔ -0.047	✔ 0.052	✖ -0.007	✖ -0.526	✖ -0.215
		Group C	✔ 0.202	✖ -0.394	✖ -0.396	✔ 0.099	✔ 0.204	✔ 0.154
		Group D	✖ -0.135	✔ -0.106	✔ -0.073	✖ -0.004	✔ 0.096	✔ 0.042
	Total Risk	Group A	✔ 0.064	✔ -0.023	✖ -0.047	✔ 0.455	✔ 0.237	✖ -0.035
		Group B	✔ 0.011	✔ 0.104	✔ 0.118	! 0.067	✖ 0.015	✔ 0.248
		Group C	✔ -0.012	✖ -0.358	! 0.025	✔ 0.231	! 0.118	✖ 0.000
		Group D	✖ -0.178	! -0.058	! 0.018	✖ -0.341	! 0.094	✖ -0.063
Single Aeration	Av.return	Group A	✖ -0.924	! 0.624	! 0.382	! 0.214	✔ 0.805	! 0.337
		Group B	✔ 0.526	✖ 0.192	✖ 0.105	✖ 0.100	✔ 0.475	✖ 0.240
		Group C	✔ 0.292	✔ 0.993	✔ 0.856	✔ 0.386	✖ -0.607	✖ 0.239
		Group D	✖ -0.491	✖ 0.357	✖ 0.130	✖ 0.034	✔ 0.400	✔ 0.442
	Total Risk	Group A	! 0.045	! 0.055	✖ -0.119	✔ -0.105	✔ 0.066	✔ 0.090
		Group B	! -0.122	✖ 0.000	✖ -0.127	✔ -0.157	✔ -0.003	! -0.171
		Group C	✔ 0.373	✖ 0.047	✔ 0.074	✔ -0.125	✔ -0.014	✔ -0.030
		Group D	✖ -0.553	✔ 0.147	✖ -0.177	✖ -0.318	✖ -0.178	✖ -0.360
Multiple Aeration	Av.return	Group A	✔ 0.331	✔ -0.273	✔ 0.129	✔ 0.139	✔ 0.556	✔ 0.401
		Group B	✔ -0.045	✔ 0.055	✔ -0.119	✔ -0.105	✔ -0.066	✔ -0.090
		Group C	✖ 0.292	✔ 0.993	✔ 0.856	✖ -0.386	✔ 0.607	✖ 0.239
		Group D	✔ 0.491	✖ 0.357	✖ 0.130	✔ 0.034	✖ -0.400	✔ 0.442
	Total Risk	Group A	✖ -0.122	✔ 0.000	✖ -0.127	✖ -0.157	✔ 0.003	✖ -0.171
		Group B	✔ 0.373	✖ -0.047	✔ 0.074	✔ -0.125	✖ -0.014	✔ -0.030
		Group C	✔ 1.249	✔ 0.567	✖ 0.108	✖ -0.570	✔ 0.456	✔ 0.244
		Group D	✖ 0.248	✖ 0.297	✔ 0.238	✔ 0.930	✖ 0.423	✖ 0.194

Fig 1: Portfolio return of multiple and single aeration and continuously flooded in ~~high risk~~high-risk zone.

~~Similarly, in Fig. 1, portfolio-weighted results of more than 70% are shown by the exclamation mark in high- and low-risk countries, respectively. In terms of total risk and average return in weighted portfolio return, the largest decoupling attitude is reported in Turkey (0.248% and 1.249%) in field burning (0.567% and 0.297%) in groups D and C, respectively.~~ Similarly, rice cultivation has the strongest attitudes toward (0.194% and 0.244%) and enteric fermentation in single aeration (-0.318% and -125%) in group C with the indicated heavy cross and checkmark. In the same scenario, the urea application in group D was 0.108%, and the corresponding value was 0.238. ~~In group D, the urea application of -0.341% with a 0.231.~~ The managed soil decoupling attitudes demonstrate -0.553% and 0.373% in single aeration in groups C and D with heavy cross and checkmark, respectively [58]. The portfolio returns have been calculated using three dimensions: continuously inundated, single aeration, and multiple aeration. In groups C and D, the rise in cultivation is 0.154 and 0.042, respectively, with a checkmark in the average return. However, the total risk of continuously flooding is estimated to be 0.000 and -0.035, with a heavy cross, indicating the least decoupling. This demonstrates that a 1 percent change in continuous flooding results in a 1 percent change in CLS-A. In the

same scenario, the single aeration result of rice cultivation indicated that the average return was 0.239 and 0.442 with a heavy cross and checkmark, and the total risk was -0.171 and -0.030 with an exclamation point and checkmark in groups B and C. It has been shown that a 1 percent change in single aeration results in a 1 percent change in CLS-A, which is comparatively higher than continuously flooded. The results of the managed soil also showed that the highest decoupling change was in groups A and B, with values of 0.045 and -0.122, respectively, as indicated by the exclamation marks. This indicates that CLS-A exhibited the highest decoupling change.

Turkey has the most positive attitude about urea application, with 0.018% and 0.025% in groups D. Hence, CLS-A from managed soils includes emissions resulting from the cultivation and management of agricultural areas, which encompassfrom cultivating and managing agricultural areas, which includes fertilizer applications, manure management, and other agricultural inputs. The combustion of crop leftovers, such as straw and stubble, is associated with field burning emissions [78]. Use of urea fertilizer in agricultural cultivation is the source of urea application emissions. The nitrogen fertilizer urea canhas the ability to release nitrous oxide, a powerful CO_2 emission. Emissions from all agricultural activities, such as manure management, field burning, urea application, enteric fermentation, and agricultural soils, make up CLS-A's total emissions.

Formatted: Indent: First line: 0.5"

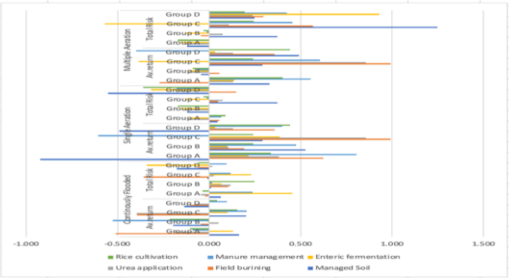

Fig 2: Portfolio return of multiple and single aeration and continuously flooded in high risk

Fig. 2 demonstrates that growing rice releases a lot of carbon dioxide. The predicted 2020 CO_2 equivalent emissions from rice agriculture were 261.53 million tons, as shown in the table. All told, this accounts for around 1.4% of the world's CO_2 emissions. Compared to intermittently flooded rice agriculture, continuously flooded rice cultivation produces greater carbon dioxide emissions [79]. This is due to the fact that, in anaerobic conditions, methane is more readily produced in continually flooded rice paddies, making them a powerful source of carbon dioxide emissions [80]. Over the years, emissions from rice farming have been on the rise. There has been a roughly 50% rise in emissions from rice cultivation since 1990, as seen in the table. Several factors have contributed to this, such as the rise in fertilizer use, the extension of rice agriculture into previously uncultivated areas (such as wetlands), and the overall increase in rice production [44].

4.2 Low-Risk Zone of CLS-A

Fig. 3 illustrates the portfolio return of low-risk countries weighted by risk and return with varying ratios in the CLS-A sector. Initially, the approximated results are analyzed for both single and multiple aerations~~aeration~~ and continuous flooding in the low-risk zone.

			Managed Soil	Field burining	Urea application	Enteric fermentation	Manure management	Rice cultivation
Continously Flooded	Av.return	Group A	✓ 0.038	✗ -0.058	✗ -0.042	✓ 0.694	! -0.280	✗ -0.111
		Group B	! -0.203	✓ 0.277	✗ 0.097	! -0.045	! -0.036	! -0.043
		Group C	✓ 0.126	✗ 0.045	✗ 0.023	✗ -0.479	✗ -0.574	✗ -0.206
		Group D	✗ -0.467	! 0.108	✓ 0.478	✗ -0.663	✓ 0.264	✓ 0.255
	Total Risk	Group A	✗ 0.278	✗ 0.414	✗ 0.141	✓ 1.832	! 0.424	✗ 0.178
		Group B	✓ 0.708	✓ 0.742	✗ 0.168	✗ 0.052	✗ 0.217	✗ 0.175
		Group C	✓ 0.832	✗ 0.326	✗ 0.200	✓ 1.356	✓ 0.780	✗ 0.254
		Group D	✓ 0.834	✓ 0.891	✓ 1.079	! 0.668	! 0.525	✓ 0.434
Single Aeration	Av.return	Group A	✓ 0.187	✗ 0.023	✓ 0.141	✗ -0.200	✓ -0.353	! -0.226
		Group B	✗ -0.401	✓ 0.504	✓ 0.123	! 0.004	✓ -0.347	✗ -0.319
		Group C	! -0.136	✗ -0.085	✗ -0.053	✗ -0.317	✗ -0.616	✓ -0.114
		Group D	! -0.021	! 0.125	✗ -0.011	✓ 0.427	✓ -0.337	✗ -0.269
	Total Risk	Group A	✗ 0.402	✗ 0.448	✓ 0.566	! 0.813	✗ 0.355	✗ 0.261
		Group B	✓ 1.043	✓ 1.344	! 0.288	✗ 0.081	✓ 0.771	✓ 0.743
		Group C	✗ 0.159	✗ 0.107	✗ 0.054	! 0.774	✓ 0.800	✓ 0.595
		Group D	! 0.589	✓ 1.390	✓ 0.405	✓ 1.540	✗ 0.415	✗ 0.392
Multiple Aeration	Av.return	Group A	✓ -0.026	✗ -0.959	✓ 0.110	! 0.237	✓ 1.006	! 0.178
		Group B	✓ -0.047	✓ -0.028	✗ -0.028	✓ 0.622	! -0.012	✓ 0.486
		Group C	! -0.186	✓ -0.167	✗ -0.086	✗ -0.085	! 0.064	✗ -0.217
		Group D	✗ -0.305	✓ -0.004	✓ 0.162	✗ -0.252	✗ -0.810	! 0.039
	Total Risk	Group A	✗ 0.231	✓ 1.169	✓ 0.366	! 0.680	✓ 1.826	! 0.614
		Group B	✗ 0.139	✗ 0.275	✗ 0.076	✓ 1.154	✗ 0.318	✓ 0.899
		Group C	✓ 0.791	✗ 0.490	! 0.245	✗ 0.566	✗ 0.216	✗ 0.287
		Group D	✓ 0.751	! 0.693	! 0.230	✗ 0.393	! 1.079	✗ 0.225

Fig. 3: Portfolio return of multiple and single aeration and continuous flooding in a low-risk zone.

The maximum weighted risk in group D is estimated to be field burning (1.390), multiple aeration (0.693), and manure management (1.079) in China, a high-risk country [81]. From about 70% in 2000 to about 75% in 2019, the percentage of CLS-A in Turkey's water abstractions has been steadily rising. This data points to the fact that CLS-A is using more and more water in Turkey. Additionally, Turkey's irrigated land share has been steadily rising, from about 20% in 2000 to about 25% in 2019. This indicates that irrigation is becoming more important for Turkish farmers when it comes to crop production [82]. Throughout the years, CLS-A's contribution to Turkey's CO_2 emissions has remained around 20%. Therefore, CLS-A probably isn't a big deal when it comes to Turkey's CO_2 emissions. From about 10% in 2000 to about 8% in 2019, CLS-A's proportion of Turkey's overall energy consumption has been steadily declining. This data points to an increase in CLS-A's energy efficiency in Turkey. It has been more common to take phosphorus from the soil in Turkey than to add it, resulting in a negative phosphorus balance since the year 2000.~~2000.~~

It appears that the soils of Turkey are losing their phosphorus content. Since the year 2000, Turkey has also had a negative nitrogen balance, which means that the country is removing more nitrogen from the soil than it is adding. This indicates that the nitrogen levels in Turkey's soils are declining. These conclusions indicate that CLS-A in Turkey is facing difficulties related to water scarcity, soil degradation, and climate change.~~These conclusions point to water scarcity, soil degradation, and climate change as some of the difficulties that CLS-A in Turkey is encountering.~~ The Turkish government must take action to address these concerns if CLS-A continues to operate~~is to continue operating~~ in the future. Consequently, the utilization of rice and urea has resulted in a 0.392 and 0.405 reduction, respectively, in agricultural productivity within group D. In the low-risk zone, group C has the highest weighted risk at 0.774, which is 1.98 times higher than group D's 1.540. Since 1990, when it was 37.9 million hectares, the irrigated area for growing rice has grown significantly, reaching 125.4 million hectares in 2020 [83]. From 1990 to 2020, the estimated emissions from rice agriculture have climbed continuously, reaching 261.530 million tons of CO_2 emissions

equivalent. Figure 4 demonstrates that rice cultivation has the greatest attitude in single aeration, which is vital~~really important~~ because rice is both a staple crop for many people's diets and a big agricultural crop in the country. Turkish rice yields have steadily increased over the previous decade, rising from a respectable 7.6 metric tons per hectare in 2013–14 to an impressive 8.30 metric tons per hectare in 2023–24.~~From a respectable 7.6 metric tons per hectare in 2013–14 to an impressive 8.30 metric tons per hectare in 2023 to 2024, Turkish rice yields have been steadily increasing over the previous decade.~~ There are three major reasons for this impressive expansion. First, let's discuss the technological advancements [84].~~As a first point, the advancements in technology [84].~~ Modern seed types, precision CLS-A methods, and integrated pest control concepts are just a few examples of how Turkish farmers are fully embracing cutting-edge agricultural technology [85].

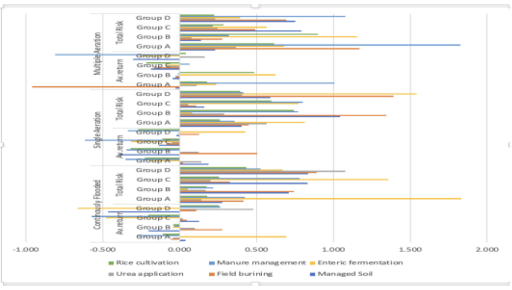

Fig 4: Portfolio return of multiple and single aeration and continuously flooded

There is no denying that these technological advancements have been the foundation of the increased rice harvests. Secondly, backing from the government: The Turkish government has done an admirable job of becoming a reliable backer of rice farming, offering crucial assistance in the form of subsidies, loans, and outreach programs. Thanks to this strategic backing, the nation's rice farming has become much more efficient and productive [86]. Thirdly, beneficial climatic conditions have emerged; recently, Turkey's climate has been highly favorable for rice cultivation.~~Thirdly, climatic provisions that are beneficial; in recent years, Turkey's climate has been great for rice growing.~~ The positive dynamics of CO_2 emissions have created an enabling environment for the enhancement of rice yields. Figure 5 illustrates that under group D, the Turkish economy and agricultural landscape have benefited greatly from the increasing rice yields achieved through enteric fermentation, manure management, and rice planting. Increased yields allow farmers to extract~~get~~ more rice out of their land, which improves their financial situation. Additionally, Turkish rice gains a competitive edge on the world stage due to a dramatic decrease in production overheads brought about by an increase in yields.

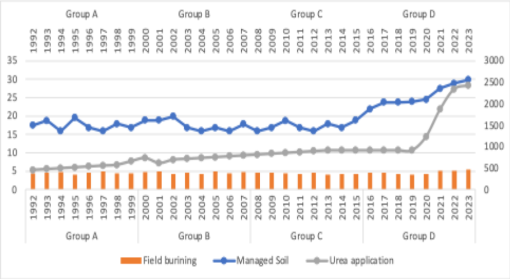

Fig. 5: Managed Soil, Field Burning,~~burning~~ and Urea application

Like the rest of the world, Turkey is facing the daunting prospect of climate change as it pertains to agricultural output. Our farming community may have to adjust to changing weather conditions and look into new agricultural technology as a safety net if we want to survive this enormous challenge and keep or increase our rice harvests for the future. Agricultural activities such as liming, field burning, and rice planting also contribute to emissions. Additionally, the table highlights how the agricultural sector plays a major part in Turkey's CO_2 emissions profile, accounting for around 20.21 percent~~percentage~~ of the overall emissions. From the table, we may deduce several important things.

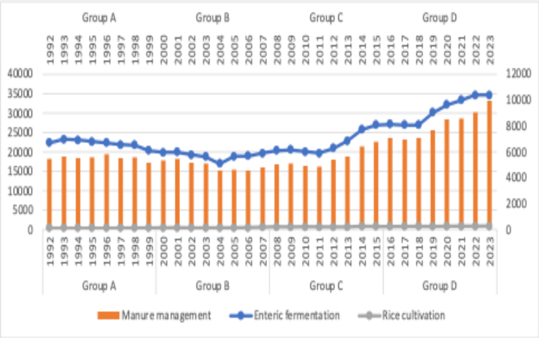

Fig. 6: Enteric fermentation, manure management and rice cultivation

The critical need to decrease emissions the data emphasizes the critical importance of the Turkish agricultural industry reducing its CO_2 emissions immediately [87, 88]. To~~In order to~~ lower Turkey's total carbon footprint and achieve its goals for mitigating climate change~~climate change mitigation goals~~, this step is crucial. Measures to decrease emissions: the agricultural sector in Turkey can decrease its CO_2 emissions by implementing a number of proven solutions. Improving manure management, reducing nitrogen fertilizer use, and switching to more environmentally friendly farming methods are all part of these steps [66].

From 2014 to 2023, the line graph in figure 6 displays the maize yields in Turkey. Crop yields for maize have been trending upwards for a while now. This trend is a response to climate change and aims to secure a future food supply, which ranks somewhat higher in group D for growing rice.~~responding to climate change and securing a future food supply, which ranks somewhat higher in group D when it comes to growing rice.~~ Rising rice harvests in Turkey are an encouraging trend [66]. Improving efficiency and output while capitalizing on favorable weather conditions is a hallmark of the remarkable transition that the country's rice farmers are presently experiencing. But we must not lose sight of the fact that increasing rice yields may not be sustainable forever [67].

In addition, the sustainable development of Turkey's agricultural sector is demonstrated by the diverse agricultural practices in terms of portfolio return under varying aeration conditions. This development has the potential to significantly enhance food security and reduce the sector's CO_2 emissions impact in terms of portfolio return. The CLS-A need is assessed for multiple and single aeration and continuously flooded separately in high- and low-risk zones with average and total risk. The portfolio returns the value of soil and agriculture,

as indicated by the estimated results. Additionally, the portfolio return has the potential to promote the development of productive, sustainable, and diverse agricultural practices in Turkey by enabling producers to make decisions that are more in accordance with market forces, thereby improving food security. The estimated results are also consistent with the stated objectives, which promote sustainable methods and fruitful cultivation, as well as the reduction of environmental degradation caused by CO_2 emissions in Turkey. Additionally, the practice of enteric fermentation, urea application, and manure management directly correlate with greenhouse gas emissions, providing an empirical foundation for evaluating the potential of a unique method to contribute significant CO_2 emissions. Consequently, the technological revolution and policy implications in agricultural methods are slowing down the process of emissions. Ultimately, these practices yield positive returns while reducing risk, providing dynamic evidence to inform sustainable policies that encourage producers to adopt more market-aligned methods and promote sustainable development, thereby contributing to both the goals of emission reduction and food security.

Formatted: Font color: Auto

5. Discussion

We are observing that the frequency of droughts, floods, and heat waves is increasing, and they are inflicting greater damage with each occurrence. It is presenting producers with additional obstacles in the areas of animal husbandry and plantation. The data in the image is not reliable for scientific interpretation as a result of the modifications. ~~-~~It is imperative to possess the most recent data on CLS-A to~~in order to~~ make informed decisions regarding agriculture [89]. ~~. Farmers have consequently restricted alternatives for cultivating produce and livestock. The information in the image is no longer applicable for scientific research as a result of these modifications.~~ Adopting CLS-A practices is necessary to mitigate the consequences of climate change on food security and nutrition [40, 90]. These methods facilitate the integration of food production and climate change adaptation, thereby providing a means to enhance food security [91]. The CLS-A program presents a critical opportunity to improve the fortunes of smallholder farmers in Turkey, including limited land and the effects of climate change. Some of the most significant CLS-A strategies are the promotion of regenerative agriculture, crop diversification, biochar application, and agroforestry, all of which enhance soil health and decrease greenhouse gas emissions [31, 92]. Both crop yield and nutrient content are directly influenced by soil health, which is fundamental to crop nutrition. By providing farmers with climate-resilient technologies and sustainable practices, such as precision agriculture and regenerative agriculture technologies, we can assist in the reversal of diminishing food quality trends [93]. Household incomes can be increased, self-sufficiency in food production can be improved, and food security can be strengthened because of the increased agricultural productivity that CLS-A has achieved. CLS-A practices are more likely to result in improved food consumption, increased dietary diversity, and enhanced overall food security in households [93].~~Farmers have consequently restricted alternatives for cultivating produce and livestock. The information in the image is no longer applicable for scientific research as a result of these modifications.~~

Additionally, the food supply and food markets are at risk due to climate change; however, these risks can be mitigated by enhancing producers' adaptive capacity and improving resource use efficiency [94]. It is imperative to adopt an integrated strategy for mitigating climate change, which involves collaboration at the local, national, and international levels. Comprehensive economic, social, and environmental strategies must be implemented by governments and stakeholders to enhance climate risk management, mitigate vulnerability, and enhance resilience [95]. As a result, the farmers have been unable to expand their options for

Formatted: Font: Times New Roman, 12 pt
Formatted: Font: Times New Roman, 12 pt
Formatted: Font: Times New Roman, 12 pt
Formatted: Font: Times New Roman, 12 pt
Formatted: Font: Times New Roman, 12 pt
Formatted: Font: Times New Roman, 12 pt
Formatted: Font: Times New Roman, 12 pt
Formatted: Font: Times New Roman, 12 pt
Formatted: Font: Times New Roman, 12 pt
Formatted: Font: Times New Roman, 12 pt
Formatted: Font: Times New Roman, 12 pt

livestock and produce cultivation. The modifications have rendered the information in the image unsuitable for scientific research. Accurate and up-to-date information regarding CLS-A should be the foundation for any modifications to agriculture policy and practice [96]. Turkey is a prominent producer of wheat, and it~~wheat~~ is a staple in the cuisines of numerous individuals worldwide [97]. Consequently, producers are applying an increased quantity of fertilizers to the soil [98].

~~.~~Farmers' adaptive capacity and resource utilization efficiency can be improved to mitigate the risks associated with climate change, which disrupts food markets and threatens the food supply [90]. An integrated strategy that involves collaboration at the local, national, and international levels is critically important for mitigating climate change in Turkey [99]. The implementation of comprehensive economic, social, and environmental strategies by governments and stakeholders is essential for the enhancement of climate risk management, the reduction of vulnerability, and the increase of resilience [100]. Climate-resilient pathways should prioritize evidence-based solutions, strengthen local institutions, harmonize agricultural and climate policies, and integrate climate and agricultural financing, as noted by the work. By emphasizing adaptable and context-driven solutions, this integrated approach is distinguished by its innovative policies and financing mechanisms [101].

Formatted: Afar (Ethiopia)

Numerous Turkish farmers are currently employing various~~a variety of~~ techniques, including crop rotation, to not only maintain the soil but also to prevent the excessive use of water. A further contributing factor to Turkey's record-breaking wheat harvest was the favorable weather [102]. Turkey has recently encountered mild winters and an abundance of rainfall in the course of the growing season [103]. That is why growing wheat yielded such excellent results. [104]. An increase in wheat harvests is a promising sign for Turkey, especially when considering the worldwide need for food.~~An increase in wheat harvests in when looking at the worldwide need for food, Turkey is a promising sign, and the country itself.~~ Turkey's dependence on imports will diminish as its wheat production increases. The conflict in Ukraine is a contributing factor to the global food shortage; therefore, it is unnecessary to disregard this issue [105]. The Turkish government should guarantee that producers continue to receive support payments to assist them in enhancing their wheat harvest [106].

6. Conclusion

Turkey can reduce maize harvest risks by taking the following steps: The CLS-A sector can improve collaboration among many agencies, strengthen an~~strengthen~~ important ministry of food, CLS-A, and livestock components, and take~~make~~ other positive actions to prevent climate change. We intend to disseminate information regarding the possible~~potential~~ effects of climate change on CLS-A and the potential measures that can be implemented to mitigate the risks. Every one of these goals has its~~its own~~ set of essential steps that will lead us to success. Our primary objective is to enhance interagency collaboration and develop capabilities. Secondly, we aim to improve the capacity to manage drought. The objective underscores the necessity of enhancing the capacity of the committees and agencies that are responsible for the administration of agricultural droughts. A third point is that there are training and research programs in place to address the impacts of local and regional climate change. It is highly recommended that the ministries of food, CLS-A, and cattle, in conjunction with their affiliated institutions, establish a special department within their respective provincial organizations to address climate change-related issues.~~First and foremost, we aim to increase interagency cooperation and build capability. Second, strengthening drought management capacity: The objective stresses the need to strengthen the capacity of the committees and agencies responsible for agricultural drought management. As a third point, there are training and research programs to deal with the effects of local and regional climate change. It is strongly~~

recommended that the ministries of food, CLS-A, and cattle, along with their affiliated institutes, establish a distinct department within their respective provincial organizations to address matters pertaining to climate change.

To adapt to the CLS-A sector's impacts of climate change, it is crucial to coordinate Turkey's policies, strategies, and legislative framework, as stated in the objective. The goal of these programs is to promote sustainable farming practices while simultaneously ensuring the safety and security of our food supply. Incorporating climate change considerations into existing policies and initiatives is crucial for effectively addressing these issues. Second, itSecond, highlights the importance of conducting and expanding scientific studies and research toin order to understand the impacts of climate change on CLS-A and to facilitate adaptation to these changes. The agricultural sector should increase its utilization of biofuels to mitigate environmental contamination. Furthermore, it is imperative to implement initiatives that prioritize financial incentives for organic agriculture, sustainable agricultural practices, and land conservation. Although the agricultural sector must decrease its reliance on fossil fuels and increase its utilization of renewable energy sources, the implementation of alternative renewable energy production methods, particularly hydroelectric systems, may result in environmental issues.

The paper focuses on the following main points. To begin, economic development goals are a common impetus for agricultural research and development projects, which have traditionally prioritized increasing production. This has led to less focus on how to adjust to a changing climate. Second, CLS-A's shift to climate revolution adaptation: The goal emphasizes the need to redirect research efforts towards adapting to climate change, especially those that focus on soil and water resource protection. –Developing irrigation systems, adjusting production patterns, increasing crop diversity, and disaster management strategies are all necessary to address the impacts of climate change on CLS-A on a national scale, especially in relation to drought. The third component of the plan is socio-economic research. This study will focus on CLS-A, food, the environment, and rural development on a national scale. It will take climate change into account. The findings of this study will pave the way for innovative policies that encourage the expansion of the country's agricultural sector in response to climate change, and they will also assist in securing the livelihoods of specific communities, such as women farmers. The fourth aspect is ensuringFourth, long-term food security.: The plan stresses building a food security system around data uncovered by R&D initiatives that account for the impacts of climate change. Aiming to meet customer expectations while responding to changing factors affecting $CO_2$$CO_2$ emissions, this system is constantlyalways evolving. As for the fifth objective, building a database and information system, it highlights the importance of developing such a system at a regional or basin scale,basin-scale, or even nationally. Lastly, the scale effect is valid in Turkey, as evidenced by a positive economic globalization coefficient. Foreign direct investment in environmentally favorable and technology-intensive practices should be promoted, as these investments have the potential to contribute to economic growth and employment. Additionally, the application of several environmental standards in international business can contribute to the mitigation of environmental contamination.

As a result, the identification of appropriate CLS-A strategies for the reduction of GHG emissions and the preservation of crop productivity necessitates a thorough examination of local environmental factors, such as soil condition, climate, and their interactions with alternative management systems. Various sociocultural, economic, and demographic factors influence the adoption of CLS-A. Although practices such as crop rotation, integrated soil fertility management, crop diversification, and intercropping are frequently implemented, their widespread adoption remains low as a result of limitations associated with farm size, infrastructure, equipment, machinery, irrigation access, extension services, and obtaining weather information. The adoption of CLS-A can be improved by providing producers with

Formatted: Font: Times New Roman, 12 pt

the necessary inputs, providing context-specific CLS-A technologies and training, and enhancing extension programs, as recommended by this study. The successful implementation of CLS-A practices can be substantially impeded by a lack of farmer awareness and expertise, as training is essential for connecting farmers with supportive institutions, reliable input suppliers, and access to credit facilities. This method can be revised on a regular basis to accommodate evolving climatic effects; it will facilitate the assessment and monitoring of the impact of climate change on agriculture.

This approach can be adjusted on a regular basis to account for changing climatic impacts; it will help evaluate and monitor how climate change is affecting farming.

Appendix A

https://data.worldbank.org/topic/agriculture-and-rural-development and https://ourworldindata.org/search?q=agriculture

Appendix BA:

		Managed Soil		Field burining		Urea application		Agriculture total		Enteric fermentation		Manure management		Rice cultivation		Agriculture total	
	Years	(Kt CO2 eq.)	(%)	[Kt CO2 eq.]	(%)	(Kt CO2 eq.)	(%)	(Kt CO2 eq.)	(%)	(kt COz eq.)	(%)	(kt COz eq.	(%)	(kt COz eq.)	(%)	(kt COz eq.)	(%)
Group A	1992	17.433	40.2	373	0.7	456	1.0	46541	100	22397	48.6	5436	11.8	100	0.2	46054	100
	1993	18.764	39.6	391	0.8	476	1.3	47654	100	23221	49.5	5657	12.1	100	0.2	46928	100
	1994	15.876	39.0	411	0.7	496	1.0	44987	100	23025	49.6	5533	11.8	100	0.2	46979	100
	1995	19.543	38.4	353	0.8	516	1.0	44651	100	22636	47.7	5597	11.8	101	0.2	47407	100
	1996	16.876	37.8	393	0.7	536	1.2	44651	100	22339	49.7	5793	12.9	100	0.2	44926	100
	1997	15.875	37.2	425	0.8	556	1.1	45761	100	21815	49.5	5523	12.5	113	0.3	44080	100
	1998	17.876	36.6	372	0.7	576	1.0	45871	100	21792	48.7	5570	12.4	126	0.3	44757	100
	1999	16.875	36.0	372	0.8	658	1.5	43988	100	20313	47.8	5166	12.2	124	0.3	42505	100
Group B	2000	18.876	35.4	408	0.8	733	1.7	44312	100	19890	45.5	5348	12.2	135	0.3	43720	100
	2001	18.888	34.8	415	0.7	617	1.5	43987	100	19963	45.1	5448	12.3	147	0.3	44276	100
	2002	19.876	37.9	357	0.8	696	1.3	47611	100	19234	45.4	5142	12.1	128	0.3	42332	100
	2003	16.876	40.2	395	0.7	715	1.4	42876	100	18714	46.9	5096	12.8	132	0.3	39894	100
	2004	15.875	39.6	357	0.7	735	1.4	48761	100	16975	45.1	4540	12.1	135	0.4	37608	100
	2005	16.876	40.2	416	0.8	755	1.5	42987	100	18874	46.5	4596	11.3	143	0.4	40558	100
	2006	15.875	39.0	380	0.7	775	1.4	42998	100	18969	45.9	4590	11.1	156	0.4	41298	100
	2007	17.777	38.4	412	0.8	795	1.3	43123	100	19680	46.4	4781	11.3	183	0.4	42439	100
Group C	2008	15.875	39.0	398	0.7	814	1.3	43211	100	20352	46.4	5027	11.5	212	0.5	43900	100
	2009	16.876	38.4	385	0.8	834	1.4	41765	100	20575	47.4	5081	11.7	203	0.5	43421	100
	2010	18.762	39.0	375	0.7	854	1.4	42987	100	20084	48.6	4929	11.9	216	0.5	41302	100
	2011	16.876	38.4	362	0.5	874	1.5	44877	100	19606	46.6	4863	11.6	208	0.5	42032	100
	2012	15.875	37.8	391	0.5	894	1.2	46876	100	20946	47.2	5391	12.1	202	0.5	44409	100
	2013	17.876	37.2	354	0.4	913	1.2	52987	100	22847	48.7	5639	12.7	204	0.4	46901	100
	2014	16.875	36.6	361	0.4	913	1.4	55766	100	25790	49.5	6425	12.2	249	0.5	52662	100
	2015	18.876	36.0	365	0.4	913	1.4	56112	100	26906	48.2	6769	12.1	231	0.4	55858	100
Group D	2016	21.876	35.4	391	0.3	913	1.4	56332	100	27154	48.3	7068	12.6	229	0.4	56219	100
	2017	23.765	34.8	389	0.3	913	2.2	56431	100	26947	48.1	6956	12.4	240	0.4	56133	100
	2018	23.743	37.3	356	0.7	913	2.3	63987	100	26984	45.8	7060	12.6	243	0.4	58894	100
	2019	23.931	35.2	354	0.8	913	1.9	54233	100	30110	47.6	7697	12.2	234	0.4	63262	100
	2020	24.543	35.8	361	0.7	1232	1.9	54322	100	32136	49.2	8508	12.8	252	0.4	65338	100
	2021	27.543	37.4	431	0.7	1872	2.3	63987	100	33368	49.1	8597	12.6	263	0.4	68023	100
	2022	28.871	39.8	432	0.8	2342	2.4	58986	100	34615	47.3	9060	12.4	262	0.4	73155	100
	2023	29.876	41.9	465	0.7	2431	2.6	63876	100	34651	46.2	9932	12.87	272	0.4	73165	100

Formatted: Heading 3, Left

Formatted: Default Paragraph Font, English (United States)

Field Code Changed

Formatted: Heading 3, Left

References

1. Erdoğan, A., Güler, M.G.: Optimization and analysis of a hydrogen supply chain in terms of cost, CO2 emissions, and risk: the case of Turkey. Int. J. Hydrogen Energy. 48, 22752–22765 (2023). https://doi.org/https://doi.org/10.1016/j.ijhydene.2023.04.300
2. Caliskan, O., Sag, N.B., Ersoy, H.K.: Thermodynamic, environmental, and exergoeconomic analysis of multi-ejector expansion transcritical CO2 supermarket refrigeration cycles in different climate regions of Türkiye: Analyse thermodynamique, environnementale et exergoéconomique des cycles de ré. Int. J. Refrig. (2024). https://doi.org/https://doi.org/10.1016/j.ijrefrig.2024.05.006
3. Uzar, U., Eyuboglu, K.: Testing the asymmetric impacts of income inequality, financial development and human development on ecological footprint in Türkiye: A NARDL approach. J. Clean. Prod. 461, 142652 (2024). https://doi.org/https://doi.org/10.1016/j.jclepro.2024.142652

4. Isik, M., Sarica, K., Ari, I.: Driving forces of Turkey’s transportation sector CO2 emissions: An LMDI approach. Transp. Policy. 97, 210–219 (2020). https://doi.org/https://doi.org/10.1016/j.tranpol.2020.07.006
5. March, A., Bennett, M., Germishuizen, M., Evans, T., Failler, P.: The status of Blue Economy development in Africa. Mar. Policy. 165, 106205 (2024). https://doi.org/https://doi.org/10.1016/j.marpol.2024.106205
6. Köprücü, Y., Acaroğlu, H.: How cereal yield is influenced by eco-environmental factors? ARDL and spectral causality analysis for Turkey. Clean. Environ. Syst. 10, 100128 (2023). https://doi.org/https://doi.org/10.1016/j.cesys.2023.100128
7. Abdallah, I., Udemba, E.N.B.T.-R.M. in S.S.: Industrial and agricultural progression amid pollution: A time series study of Turkey. Presented at the (2023)
8. Knorr, S., Weisener, C.G., Phillips, L.A.: Agricultural land management alters the biogeochemical cycling capacity of aquatic and sediment environments. Agric. Ecosyst. Environ. 357, 108661 (2023). https://doi.org/https://doi.org/10.1016/j.agee.2023.108661
9. Sharma, S., Wait, D.A., Khanal, C., Khanal, P.: Native bamboo Arundinaria gigantea restoration for climate smart land management: A review. Adv. Bamboo Sci. 10, 100125 (2025). https://doi.org/https://doi.org/10.1016/j.bamboo.2025.100125
10. Bowditch, E., Santopuoli, G., Neroj, B., Svetlik, J., Tominlson, M., Pohl, V., Avdagić, A., del Rio, M., Zlatanov, T., Maria, H., Jamnická, G., Serengil, Y., Sarginci, M., Brynleifsdóttir, S.J., Lesinki, J., Azevedo, J.C.: Application of climate-smart forestry – Forest manager response to the relevance of European definition and indicators. Trees, For. People. 9, 100313 (2022). https://doi.org/https://doi.org/10.1016/j.tfp.2022.100313
11. Naimoglu, M., Akal, M.: The relationship between energy technology, energy efficiency, renewable energy, and the environment in Türkiye. J. Clean. Prod. 418, 138144 (2023). https://doi.org/https://doi.org/10.1016/j.jclepro.2023.138144
12. Deng, P., Tan, X., Bai, Y., Li, H.: Forecasting chaotic behavior of the drill bit in real-time using a DNN model integrated with rock surface morphology. Mech. Syst. Signal Process. 221, 111710 (2024). https://doi.org/https://doi.org/10.1016/j.ymssp.2024.111710
13. Zul Azlan, Z.H., Junaini, S.N., Bolhassan, N.A., Wahi, R., Arip, M.A.: Harvesting a sustainable future: An overview of smart agriculture’s role in social, economic, and environmental sustainability. J. Clean. Prod. 434, 140338 (2024). https://doi.org/https://doi.org/10.1016/j.jclepro.2023.140338
14. Büyüközkan, G., Uztürk, D.: Integrated design framework for smart agriculture: Bridging the gap between digitalization and sustainability. J. Clean. Prod. 449, 141572 (2024). https://doi.org/https://doi.org/10.1016/j.jclepro.2024.141572
15. Ağbulut, Ü.: Forecasting of transportation-related energy demand and CO2 emissions in Turkey with different machine learning algorithms. Sustain. Prod. Consum. 29, 141–157 (2022). https://doi.org/https://doi.org/10.1016/j.spc.2021.10.001
16. Yurtkuran, S.: The effect of agriculture, renewable energy production, and globalization on CO2 emissions in Turkey: A bootstrap ARDL approach. Renew. Energy. 171, 1236–1245 (2021). https://doi.org/https://doi.org/10.1016/j.renene.2021.03.009
17. Goldenberg, M.G., Burian, A., Seppelt, R., Santibañez Ossa, F.A., Bagnato, C.E., Satorre, E.H., Martini, G.D., Garibaldi, L.A.: Effects of natural habitat composition and configuration, environment and agricultural input on soybean and maize yields in Argentina. Agric. Ecosyst. Environ. 339, 108133 (2022). https://doi.org/https://doi.org/10.1016/j.agee.2022.108133

18. Isik, M., Ari, I., Sarica, K.: Challenges in the CO2 emissions of the Turkish power sector: Evidence from a two-level decomposition approach. Util. Policy. 70, 101227 (2021). https://doi.org/https://doi.org/10.1016/j.jup.2021.101227
19. Coruh, E., Bilgic, A., Cengiz, V., Urak, F.: Uncovering the determinants of bottom-up CO2 emissions among households in Türkiye: Analysis and policy recommendations. J. Clean. Prod. 469, 143197 (2024). https://doi.org/https://doi.org/10.1016/j.jclepro.2024.143197
20. Onenli, O., Ercan, H., Acikgoz, S., Kat, B.: Emission reduction pathways for satisfying Türkiye's future electricity demand: A cross-country dynamic panel analysis with linear programming scenarios. Clean. Energy Syst. 6, 100085 (2023). https://doi.org/https://doi.org/10.1016/j.cles.2023.100085
21. Karki, E., Sharma, A., Timsina, P., Chaudhary, A., Sharma, R., Brown, B.: Strategies to overcome stagnation in agricultural adoption despite awareness and interest: a case study of conservation agriculture in South Asia. Renew. Agric. Food Syst. 39, e14 (2024). https://doi.org/DOI: 10.1017/S1742170524000073
22. Alatawneh, A., Ghunaim, D.: Towards vehicle electrification: A mathematical prediction of battery electric vehicle ownership growth, the case of Turkey. Green Energy Intell. Transp. 100166 (2024). https://doi.org/https://doi.org/10.1016/j.geits.2024.100166
23. Tayyar, A.E., Gasim, N., Biçen, Ö.F., Mukhtarov, S.: An analysis of climate impacts on agriculture production: Evidence from Türkiye by BMA and A-ARDL approaches. J. Environ. Manage. 389, 126111 (2025). https://doi.org/https://doi.org/10.1016/j.jenvman.2025.126111
24. Temizceri, F.T., Kara, S.S.: Towards sustainable logistics in Turkey: A bi-objective approach to green intermodal freight transportation enhanced by machine learning. Res. Transp. Bus. Manag. 55, 101145 (2024). https://doi.org/https://doi.org/10.1016/j.rtbm.2024.101145
25. Adebayo, T.S., Agyekum, E.B., Kamel, S., Zawbaa, H.M., Altuntaş, M.: Drivers of environmental degradation in Turkey: Designing an SDG framework through advanced quantile approaches. Energy Reports. 8, 2008–2021 (2022). https://doi.org/https://doi.org/10.1016/j.egyr.2022.01.020
26. Guven, D., Kayalica, M.O.: Analysing the determinants of the Turkish household electricity consumption using gradient boosting regression tree. Energy Sustain. Dev. 77, 101312 (2023). https://doi.org/https://doi.org/10.1016/j.esd.2023.101312
27. Türker, Y.Ö., Aydin, A.: How ready is the Turkish Legislation for the green deal? Energy Clim. Chang. 3, 100084 (2022). https://doi.org/https://doi.org/10.1016/j.egycc.2022.100084
28. Bakay, M.S., Ağbulut, Ü.: Electricity production based forecasting of greenhouse gas emissions in Turkey with deep learning, support vector machine and artificial neural network algorithms. J. Clean. Prod. 285, 125324 (2021). https://doi.org/https://doi.org/10.1016/j.jclepro.2020.125324
29. As, M., Bilir, T.: Enhancing energy efficiency and cost-effectiveness while reducing CO2 emissions in a hospital building. J. Build. Eng. 78, 107792 (2023). https://doi.org/https://doi.org/10.1016/j.jobe.2023.107792
30. Tao, W., Zhao, L., Wang, G., Liang, R.: Review of the internet of things communication technologies in smart agriculture and challenges. Comput. Electron. Agric. 189, 106352 (2021). https://doi.org/https://doi.org/10.1016/j.compag.2021.106352
31. Tuğaç, Ç.: Climate change adaptation: The missing component in the local climate change action plans of Turkish metropolitan municipalities. Urban Clim. 61, 102396

(2025). https://doi.org/https://doi.org/10.1016/j.uclim.2025.102396
32. Gungor, G., Sari, R.: Meta-analysis of the Turkish energy and climate pathways to achieve the net-zero emission target. Energy. 305, 132337 (2024). https://doi.org/https://doi.org/10.1016/j.energy.2024.132337
33. Yazar, M., Daloglu Cetinkaya, I., Baykal Fide, E., Haarstad, H.: Diffusion of global climate policy: National depoliticization, local repoliticization in Turkey. Glob. Environ. Chang. 81, 102699 (2023). https://doi.org/https://doi.org/10.1016/j.gloenvcha.2023.102699
34. Acar, S., Kat, B., Rogner, M., Saygin, D., Taranto, Y., Yeldan, A.E.: Transforming Türkiye's power system: An assessment of economic, social, and external impacts of an energy transition by 2030. Clean. Energy Syst. 4, 100064 (2023). https://doi.org/https://doi.org/10.1016/j.cles.2023.100064
35. Gökçek, M., Paltrinieri, N., Liu, Y., Badia, E., Dokuz, A.Ş., Erdoğmuş, A., Urhan, B.B., Yoldaş, Ö.: Optimum sizing of hybrid renewable power systems for on-site hydrogen refuelling stations: Case studies from Türkiye and Spain. Int. J. Hydrogen Energy. 59, 715–729 (2024). https://doi.org/https://doi.org/10.1016/j.ijhydene.2024.02.068
36. Nayir, T., Uskun, E., Ilter, H., Ozkan, S., Kiraz, E.D.E.: Developing a climate change health literacy scale: A methodological study in Turkish adults. J. Clim. Chang. Heal. 23, 100451 (2025). https://doi.org/https://doi.org/10.1016/j.joclim.2025.100451
37. Raihan, A., Hasan, M.A., Voumik, L.C., Pattak, D.C., Akter, S., Ridwan, M.: Sustainability in Vietnam: Examining economic growth, energy, innovation, agriculture, and forests' impact on CO2 emissions. World Dev. Sustain. 4, 100164 (2024). https://doi.org/https://doi.org/10.1016/j.wds.2024.100164
38. Zakaria, A., Azumah, S.B., Appiah-Twumasi, M., Dagunga, G.: Adoption of climate-smart agricultural practices among farm households in Ghana: The role of farmer participation in training programmes. Technol. Soc. 63, 101338 (2020). https://doi.org/https://doi.org/10.1016/j.techsoc.2020.101338
39. Boz, Y., Cay, T.: How smart and sustainable are the cities in Turkiye? - National policies and the enthusiasm level of the local governments. Heliyon. 10, e26002 (2024). https://doi.org/https://doi.org/10.1016/j.heliyon.2024.e26002
40. Bhatnagar, S., Chaudhary, R., Sharma, S., Janjhua, Y., Thakur, P., Sharma, P., Keprate, A.: Exploring the dynamics of climate-smart agricultural practices for sustainable resilience in a changing climate. Environ. Sustain. Indic. 24, 100535 (2024). https://doi.org/https://doi.org/10.1016/j.indic.2024.100535
41. Karakurt, I., Aydin, G.: Development of regression models to forecast the CO2 emissions from fossil fuels in the BRICS and MINT countries. Energy. 263, 125650 (2023). https://doi.org/https://doi.org/10.1016/j.energy.2022.125650
42. Cekinir, S., Ozgener, O., Ozgener, L.: Türkiye's energy projection for 2050. Renew. Energy Focus. 43, 93–116 (2022). https://doi.org/https://doi.org/10.1016/j.ref.2022.09.003
43. Eslami, S., Noorollahi, Y., Marzband, M., Anvari-Moghaddam, A.: District heating planning with focus on solar energy and heat pump using GIS and the supervised learning method: Case study of Gaziantep, Turkey. Energy Convers. Manag. 269, 116131 (2022). https://doi.org/https://doi.org/10.1016/j.enconman.2022.116131
44. Destek, M.A.: Deindustrialization, reindustrialization and environmental degradation: Evidence from ecological footprint of Turkey. J. Clean. Prod. 296, 126612 (2021). https://doi.org/https://doi.org/10.1016/j.jclepro.2021.126612
45. Yilanci, V., Kilci, E.N.: A study on the macroeconomic and financial determinants of telecommunication infrastructure: Evidence from Turkiye. Technol. Soc. 77, 102573

(2024). https://doi.org/https://doi.org/10.1016/j.techsoc.2024.102573
46. Khan, R.: Beta decoupling relationship between CO2 emissions by GDP, energy consumption, electricity production, value-added industries, and population in China. PLoS One. 16, 1–22 (2021). https://doi.org/10.1371/journal.pone.0249444
47. Khan, R.: The impact of a new techno-nationalism era on eco-economic decoupling. Resour. Policy. 82, 103452 (2023). https://doi.org/https://doi.org/10.1016/j.resourpol.2023.103452
48. Khan, R.: Catch-up growth with alpha and beta decoupling and their relationships between CO2 emissions by GDP, population, energy production, and consumption. Heliyon. e31470 (2024). https://doi.org/https://doi.org/10.1016/j.heliyon.2024.e31470
49. Khan, R.: Comparing the effects of agricultural intensification on CO2 emissions and energy consumption in developing and developed countries. Front. Environ. Sci. https://doi.org/10.3389/fenvs.2022.1065634
50. Wang, C., Abbasi, K.R., Irfan, M., Ben-Salha, O., Bandyopadhyay, A.: Navigating sustainability in the US: A comprehensive analysis of green energy, eco-innovation, and economic policy uncertainty on sectoral CO2 emissions. Energy Reports. 11, 5286–5299 (2024). https://doi.org/https://doi.org/10.1016/j.egyr.2024.05.014
51. Khan, R.: The impact of agricultural intensification on carbon dioxide emissions and energy consumption: a comparative study of developing and developed nations.
52. Erdoğan, A., Geçici, E., Güler, M.G.: Design of a future hydrogen supply chain: A multi-objective model for Turkey. Int. J. Hydrogen Energy. 48, 11775–11789 (2023). https://doi.org/https://doi.org/10.1016/j.ijhydene.2022.12.071
53. Khan, S.Z.R.: Performance evaluation of e-commerce firms in China: Using three-stage data envelopment analysis and the Malmquist productivity index. PLoS One. 11, (2021). https://doi.org/10.1371/journal.pone.0255851
54. Rabnawaz Khan, Y.K.: Effects of Energy Consumption on GDP: New Evidence of 24 Countries on Their Natural Resources and Production of Electricity. Ekonomika. 99, 26–49 (2020). https://doi.org/https://doi.org/10.15388/Ekon.2020.1.2
55. Gürel, B., Kurtuluş, K., Yurdakul, S., Karaca Dolgun, G., Akman, R., Önür, M.E., Varol, M., Keçebaş, A., Gürbüz, H.: Combustion of chicken manure and Turkish lignite mixtures in a circulating fluidized bed. Renew. Sustain. Energy Rev. 189, 113960 (2024). https://doi.org/https://doi.org/10.1016/j.rser.2023.113960
56. Kınay, U., Laukkarinen, A., Vinha, J.: Renovation wave of the residential building stock targets for the carbon-neutral: Evaluation by Finland and Türkiye case studies for energy demand. Energy Sustain. Dev. 75, 1–24 (2023). https://doi.org/https://doi.org/10.1016/j.esd.2023.04.014
57. Zambrano-Monserrate, M.A.: Clean energy production index and CO2 emissions in OECD countries. Sci. Total Environ. 907, 167852 (2024). https://doi.org/https://doi.org/10.1016/j.scitotenv.2023.167852
58. Atmaca, A., Atmaca, N.: Carbon footprint assessment of residential buildings, a review and a case study in Turkey. J. Clean. Prod. 340, 130691 (2022). https://doi.org/https://doi.org/10.1016/j.jclepro.2022.130691
59. Jonek-Kowalska, I.: Towards the reduction of CO2 emissions. Paths of pro-ecological transformation of energy mixes in European countries with an above-average share of coal in energy consumption. Resour. Policy. 77, 102701 (2022). https://doi.org/https://doi.org/10.1016/j.resourpol.2022.102701
60. Najmuddin, Omaid, Zhihui Li, Rabnawaz Khan, and W.Z.: Valuation of Land-Use/Land-Cover-Based Ecosystem Services in Afghanistan—An Assessment of the Past and Future. Land. 11, (2022)
61. Jebabli, I., Lahiani, A., Mefteh-Wali, S.: Quantile connectedness between CO2

emissions and economic growth in G7 countries. Resour. Policy. 81, 103348 (2023). https://doi.org/https://doi.org/10.1016/j.resourpol.2023.103348
62. Yin, C., Wang, C., Wang, Q., Ge, Y.: Effects of regional freight structure and energy intensity on CO2 emission of transport—a case study in Yangtze River Delta. Int. J. Sustain. Transp. 18, 379–392 (2024). https://doi.org/https://doi.org/10.1080/15568318.2023.2299918
63. Acaroğlu, H., Güllü, M.: Climate change caused by renewable and non-renewable energy consumption and economic growth: A time series ARDL analysis for Turkey. Renew. Energy. 193, 434–447 (2022). https://doi.org/https://doi.org/10.1016/j.renene.2022.04.138
64. Jahanger, A., Hossain, M.R., Awan, A.: Exploring the critical nexus among energy mineral, globalization, and CO2 emissions in NAFTA: What's the forum's response amid asymmetries? Resour. Policy. 90, 104825 (2024). https://doi.org/https://doi.org/10.1016/j.resourpol.2024.104825
65. Schilling, M.A., Esmundo, M.: Technology S-curves in renewable energy alternatives: Analysis and implications for industry and government. Energy Policy. 37, 1767–1781 (2009). https://doi.org/https://doi.org/10.1016/j.enpol.2009.01.004
66. Shahbaz, M., Patel, N., Du, A.M., Ahmad, S.: From black to green: Quantifying the impact of economic growth, resource management, and green technologies on CO2 emissions. J. Environ. Manage. 360, 121091 (2024). https://doi.org/https://doi.org/10.1016/j.jenvman.2024.121091
67. Sufyanullah, K., Ahmad, K.A., Sufyan Ali, M.A.: Does emission of carbon dioxide is impacted by urbanization? An empirical study of urbanization, energy consumption, economic growth and carbon emissions - Using ARDL bound testing approach. Energy Policy. 164, 112908 (2022). https://doi.org/https://doi.org/10.1016/j.enpol.2022.112908
68. ExxonMobil: Advancing climate solutions. (2022)
69. Lioudis, N.: What is the relationship between oil prices and inflation?, http://www.investopedia.com/ask/answers/06/oilpricesinflation.asp
70. Zhang, S., Zhang, X., Du, J., Wang, W., Pi, X.: Multi-target meridians classification based on the topological structure of anti-cancer phytochemicals using deep learning. J. Ethnopharmacol. 319, 117244 (2024). https://doi.org/https://doi.org/10.1016/j.jep.2023.117244
71. Ridoutt, B., Lehnert, S.A., Denman, S., Charmley, E., Kinley, R., Dominik, S.: Potential GHG emission benefits of Asparagopsis taxiformis feed supplement in Australian beef cattle feedlots. J. Clean. Prod. 337, 130499 (2022). https://doi.org/https://doi.org/10.1016/j.jclepro.2022.130499
72. Sheraz, M., Deyi, X., Sinha, A., Mumtaz, M.Z., Fatima, N.: The dynamic nexus among financial development, renewable energy and carbon emissions: Moderating roles of globalization and institutional quality across BRI countries. J. Clean. Prod. 343, 130995 (2022). https://doi.org/https://doi.org/10.1016/j.jclepro.2022.130995
73. Li, D., Guan, X., Tang, T., Zhao, L., Tong, W., Wang, Z.: The clean energy development path and sustainable development of the ecological environment driven by big data for mining projects. J. Environ. Manage. 348, 119426 (2023). https://doi.org/https://doi.org/10.1016/j.jenvman.2023.119426
74. Rehfeldt, M., Fleiter, T., Herbst, A., Eidelloth, S.: Fuel switching as an option for medium-term emission reduction - A model-based analysis of reactions to price signals and regulatory action in German industry. Energy Policy. 147, 111889 (2020). https://doi.org/https://doi.org/10.1016/j.enpol.2020.111889
75. Deng, X., Du, L.: Estimating the environmental efficiency, productivity, and shadow

price of carbon dioxide emissions for the Belt and Road Initiative countries. J. Clean. Prod. 277, 123808 (2020). https://doi.org/https://doi.org/10.1016/j.jclepro.2020.123808
76. Shukla, N., Shah, K., Rathore, D., Soni, K., Shah, J., Vora, H., Dave, H.: Androgen receptor: Structure, signaling, function and potential drug discovery biomarker in different breast cancer subtypes. Life Sci. 348, 122697 (2024). https://doi.org/https://doi.org/10.1016/j.lfs.2024.122697
77. Van Poecke, A., Tabari, H., Hellinckx, P.: Unveiling the backbone of the renewable energy forecasting process: Exploring direct and indirect methods and their applications. Energy Reports. 11, 544–557 (2024). https://doi.org/https://doi.org/10.1016/j.egyr.2023.12.031
78. Yang, X., Ramos-Meza, C.S., Shabbir, M.S., Ali, S.A., Jain, V.: The impact of renewable energy consumption, trade openness, CO2 emissions, income inequality, on economic growth. Energy Strateg. Rev. 44, 101003 (2022). https://doi.org/https://doi.org/10.1016/j.esr.2022.101003
79. Perone, G.: The relationship between renewable energy production and CO2 emissions in 27 OECD countries: A panel cointegration and Granger non-causality approach. J. Clean. Prod. 434, 139655 (2024). https://doi.org/https://doi.org/10.1016/j.jclepro.2023.139655
80. Yu, Y., Radulescu, M., Ifelunini, A.I., Ogwu, S.O., Onwe, J.C., Jahanger, A.: Achieving Carbon Neutrality Pledge through Clean Energy Transition: Linking the Role of Green Innovation and Environmental Policy in E7 Countries. Energies. 15, (2022). https://doi.org/10.3390/en15176456
81. Mendonça, A.K. de S., de Andrade Conradi Barni, G., Moro, M.F., Bornia, A.C., Kupek, E., Fernandes, L.: Hierarchical modeling of the 50 largest economies to verify the impact of GDP, population and renewable energy generation in CO2 emissions. Sustain. Prod. Consum. 22, 58–67 (2020). https://doi.org/https://doi.org/10.1016/j.spc.2020.02.001
82. Ozdemir, A.C.: Decomposition and decoupling analysis of carbon dioxide emissions in electricity generation by primary fossil fuels in Turkey. Energy. 273, 127264 (2023). https://doi.org/https://doi.org/10.1016/j.energy.2023.127264
83. Adebayo, T.S., Acheampong, A.O.: Modelling the globalization-CO2 emission nexus in Australia: evidence from quantile-on-quantile approach. Environ. Sci. Pollut. Res. 29, 9867–9882 (2022). https://doi.org/10.1007/s11356-021-16368-y
84. Grodzicki, T., Jankiewicz, M.: The impact of renewable energy and urbanization on CO2 emissions in Europe – Spatio-temporal approach. Environ. Dev. 44, 100755 (2022). https://doi.org/https://doi.org/10.1016/j.envdev.2022.100755
85. Kwilinski, A., Lyulyov, O., Pimonenko, T.: Reducing transport sector CO2 emissions patterns: environmental technologies and renewable energy. J. Open Innov. Technol. Mark. Complex. 100217 (2024). https://doi.org/https://doi.org/10.1016/j.joitmc.2024.100217
86. Peiseler, L., Cabrera Serrenho, A.: How can current German and EU policies be improved to enhance the reduction of CO2 emissions of road transport? Revising policies on electric vehicles informed by stakeholder and technical assessments. Energy Policy. 168, 113124 (2022). https://doi.org/https://doi.org/10.1016/j.enpol.2022.113124
87. Kłosowski, G., Hoła, A., Rymarczyk, T., Mazurek, M., Niderla, K., Rzemieniak, M.: Use of the double-stage LSTM network in electrical tomography for 3D wall moisture imaging. Measurement. 213, 112741 (2023). https://doi.org/https://doi.org/10.1016/j.measurement.2023.112741

88. Llanos, C., Kristjanpoller, W., Michell, K., Minutolo, M.C.: Causal treatment effects in time series: CO2 emissions and energy consumption effect on GDP. Energy. 249, 123625 (2022). https://doi.org/https://doi.org/10.1016/j.energy.2022.123625
89. Meygoonpoury, E., Ghadim, M.K., Ziabakhsh-Ganji, Z.: Internationalization of renewable energy base businesses with a combined approach to networking and collaborative competition. Results Eng. 21, 101726 (2024). https://doi.org/https://doi.org/10.1016/j.rineng.2023.101726
90. Cevher, C., Feridun, G., Ebegil, M., Özkan, U., Coskun-Cevher, S.: Analysis of the effects of climate change on rangeland livestock: The example of the Central Anatolia Region in Türkiye. Small Rumin. Res. 245, 107464 (2025). https://doi.org/https://doi.org/10.1016/j.smallrumres.2025.107464
91. Ntawuruhunga, D., Ngowi, E.E., Mangi, H.O., Salanga, R.J., Shikuku, K.M.: Geospatial characterization of climate-smart agroforestry in two contrasting physiographic zones of Rwanda. Trees, For. People. 14, 100446 (2023). https://doi.org/https://doi.org/10.1016/j.tfp.2023.100446
92. Zaigham Abbas Naqvi, S.M., Hussain, S., Awais, M., Tahir, M.N., Saleem, S.R., Al-Yarimi, F.A.M., Ashurov, M., Saidani, O., Khan, M.I., Wu, J., Wei, Z., Hu, J.: Climate-resilient water management: Leveraging IoT and AI for sustainable agriculture. Egypt. Informatics J. 30, 100691 (2025). https://doi.org/https://doi.org/10.1016/j.eij.2025.100691
93. Avila, F.R. de, Barbosa, J.L.V.: Smart environments in digital agriculture: a systematic review and taxonomy. Comput. Electron. Agric. 236, 110393 (2025). https://doi.org/https://doi.org/10.1016/j.compag.2025.110393
94. Mdoe, C.N., Mahonge, C.P., Ngowi, E.E.: Mapping the trends, knowledge production, and practices of climate-smart aquaculture scholarship: Empirical insights from bibliometric analysis. Aquaculture. 598, 741939 (2025). https://doi.org/https://doi.org/10.1016/j.aquaculture.2024.741939
95. Kutlu Gündoğdu, F.: Analyzing critical barriers of smart energy city in Turkey based on two-dimensional uncertainty by hesitant z-fuzzy linguistic terms. Eng. Appl. Artif. Intell. 113, 104935 (2022). https://doi.org/https://doi.org/10.1016/j.engappai.2022.104935
96. Cui, Y., Khan, S.U., Deng, Y., Zhao, M.: Spatiotemporal heterogeneity, convergence and its impact factors: Perspective of carbon emission intensity and carbon emission per capita considering carbon sink effect. Environ. Impact Assess. Rev. 92, 106699 (2022). https://doi.org/https://doi.org/10.1016/j.eiar.2021.106699
97. Arora, T., Reddy, C.S., Sharma, R., Kilaparthi, S.D., Gupta, L.: Greenhouse gas emissions of Delhi, India: A trend analysis of sources and sinks for 2017–2021. Urban Clim. 51, 101634 (2023). https://doi.org/https://doi.org/10.1016/j.uclim.2023.101634
98. Khan, R., Zhuang, W., Najumddin, O., Butt, R.S., Ahmad, I., Al-Faryan, M.A.S.: The impact of agricultural intensification on carbon dioxide emissions and energy consumption: A comparative study of developing and developed nations. Front. Environ. Sci. 10, (2022). https://doi.org/10.3389/fenvs.2022.1036300
99. Shaikh, T.A., Rasool, T., Mir, W.A.: Fields of the future: Digital transformation in smart agriculture with large language models and generative AI. Comput. Stand. Interfaces. 94, 104005 (2025). https://doi.org/https://doi.org/10.1016/j.csi.2025.104005
100. Ahsan, T.M.A., Hassan, A.A., Thakur, A.K., Ahamed, M.S.: Transitioning from conventional systems to ground source heat pump for climate-smart greenhouse conditioning. Case Stud. Therm. Eng. 72, 106314 (2025). https://doi.org/https://doi.org/10.1016/j.csite.2025.106314
101. Morchid, A., Alblushi, I.G., Khalid, H.M., Alami, R. El, Said, Z., Qjidaa, H., Cuce, E.,

Muyeen, S.M., Jamil, M.O.: Fire detection and anti-fire system to enhance food security: A concept of smart agriculture systems-based IoT and embedded systems with machine-to-machine protocol. Sci. African. 27, e02559 (2025). https://doi.org/https://doi.org/10.1016/j.sciaf.2025.e02559
102. Ridzuan, N.H.A.M., Marwan, N.F., Khalid, N., Ali, M.H., Tseng, M.-L.: Effects of agriculture, renewable energy, and economic growth on carbon dioxide emissions: Evidence of the environmental Kuznets curve. Resour. Conserv. Recycl. 160, 104879 (2020). https://doi.org/https://doi.org/10.1016/j.resconrec.2020.104879
103. Christidis, P., Vetters, N., Ulpiani, G., Guardiola, M.O., Soler, J.R.L., Radics, M., Gonzalo, M.V.: Urban transport in the EU Mission on Climate Neutral and Smart Cities: preliminary data from applicants' expressions of interest. Transp. Res. Procedia. 72, 580–587 (2023). https://doi.org/https://doi.org/10.1016/j.trpro.2023.11.442
104. Chang, T., Hsu, C.-M., Chen, S.-T., Wang, M.-C., Wu, C.-F.: Revisiting economic growth and CO2 emissions nexus in Taiwan using a mixed-frequency VAR model. Econ. Anal. Policy. 79, 319–342 (2023). https://doi.org/https://doi.org/10.1016/j.eap.2023.05.022
105. Islam, M.J., Datta, R., Iqbal, A.: Actual rating calculation of the zoom cloud meetings app using user reviews on google play store with sentiment annotation of BERT and hybridization of RNN and LSTM. Expert Syst. Appl. 223, 119919 (2023). https://doi.org/https://doi.org/10.1016/j.eswa.2023.119919
106. Ben Jebli, M., Farhani, S., Guesmi, K.: Renewable energy, CO2 emissions and value added: Empirical evidence from countries with different income levels. Struct. Chang. Econ. Dyn. 53, 402–410 (2020). https://doi.org/https://doi.org/10.1016/j.strueco.2019.12.009

Authors' Response to Reviewers' Comments

Subject: PLOS ONE Decision: Revision required [PONE-D-25-27665]

Dear Dr. Khan,

PONE-D-25-27665

Title: **Exploring Climate Smart Agriculture in Turkey: Enhancing Food Security and Sustainable Practices for the Reduction of CO2 Emissions**

Dear editor and reviewers,

Thank you very much for your letter and reviewers' valuable suggestions on our manuscript entitled "**Exploring Climate Smart Agriculture in Turkey: Enhancing Food Security and Sustainable Practices for the Reduction of CO2 Emissions"** No: [PONE-D-25-27665]. Those comments are precious and helpful for improving our paper and providing the essential guiding significance to our research. We have carefully checked and improved the manuscript according to the editor's and reviewers' comments.

REVIEWER #1:

Reviewer #1: This study explores the role of Climate Smart Agriculture (CSA) in Turkey for food security and CO_2 emission reduction, which is a topic of practical significance. However, the manuscript has significant methodological flaws, conceptual confusion, and issues with data interpretation, severely affecting the reliability of the conclusions.

1. Conceptual and Terminological Confusion
- Misuse of Key Terms: The manuscript consistently fails to clearly define the core dimensions of CSA (mitigation, adaptation, increased productivity).
- Misapplication of Decoupling Concept:
- Decoupling should measure the decoupling of economic growth from carbon emissions (e.g., using Tapio's decoupling index), but the manuscript forcibly links it to financial indicators such as "portfolio return" (Abstract, Sec 3.1), lacking theoretical basis.
- For example: "The decoupling process is responsible for estimating the percentage change in portfolio returns" (Abstract) — there is a missing logical connection between environmental science and financial models.

Reply: I appreciate your assistance in supplying the specified text from the Canvas. We comprehend that you would like a response to the statement concerning the conceptual and terminological confusion in the manuscript.

The following is a response that addresses the concerns that were raised:

" We appreciate the comprehensive feedback you provided regarding the conceptual and terminological clarity of the manuscript." We acknowledge the arguments concerning:

It is imperative that the fundamental dimensions of Climate-Smart Agriculture (CSA), which are specifically mitigation, adaptation, and increased productivity, be defined in a more explicit and precise manner. We concur with this proposition. In order to guarantee consistent application throughout the text and incorporate precise definitions, we will revise the manuscript (Heading 1, 1st paragraph).

Regarding the decoupling concept, we comprehend your apprehension regarding the coerced connection between 'decoupling' and financial indicators, such as 'portfolio return.' In this study, we first estimated the decoupling and then portfolio return, focusing on its relationship to carbon emissions or other relevant environmental metrics, in accordance with established environmental science principles. We acknowledge that the standard definition of decoupling is related to economic growth and carbon emissions by decoupling and portfolio return. In conclusion, the abstract section has been revised and updated.

2. Methodological Deficiencies
- The use of "portfolio weighted risk return" (Sec 3.3) to analyze agricultural emissions is not justified. The causal relationship between financial indicators (such as beta values, risk premiums) and CO_2 emissions is not argued.
- Ambiguous grouping criteria: Turkey is divided into Groups A-D (Sec 4.1), but the criteria for grouping (geographical? Economic level?) are not explained.
- Data Handling Issues:
- Data sources are only mentioned as "World Bank" (Sec 3.2), without providing specific datasets or access links, violating PLOS data policy (Page 4-5).
- Anomalies in Appendix A tables (e.g., in 1996, the "Agriculture total (N)" column shows 100/52297; in 2020, urea application value 12327 is much higher than other years) are not explained.

Reply: Thank you very much for your valuable comments and suggestions. According to your valued comments, we have revised it carefully as follows.

There has been an update and revision made to heading 3.3 in accordance with the casual relationship that exists between the financial indicators in the first paragraph.

Your insightful commentary regarding the ambiguity of the classification criteria (Groups A-D) in this manuscript is greatly appreciated. You are correct that the document does not expressly define the criteria for these groups (A, B, C, D) nor does it specify that they are divided by specific years (e.g., 1992-2023). The decoupling operation is divided into four distinct sections (A–D) for the purpose of analysis in the first paragraph of heading 3.2. It has been updated and revised. The territory of Turkey is not defined, as we only distributed the number of years into four groups, despite the fact that all of the subsets cover a complete five years. The analysis has interpreted these groupings based on general possibilities, including varying farm types, regions, management strategies, and socio-economic contexts.

We are grateful for your feedback on the data handling and the mention of "World Bank" as a data source without specific links, which you identified as a potential violation of PLOS data policy. We are grateful for your highlighting of this critical point in heading 3.2. We have updated and revised our analysis to reflect that it did indeed use datasets from both the **World Bank (WB)** and **Our Data in the World**. All of the subsets analyzed were analyzed over a full five-year period, and the analysis was conducted using panel data. Detailed information about the precise datasets used, including their specific names and direct access links, is comprehensively provided in **Appendix B** of the manuscript. We are committed to ensuring that this appendix is updated to meet the highest standards of data transparency and reproducibility.

We are grateful for your identification of these particular data management concerns. Revised and updated heading 3.2 (last lines of the first paragraph). To assure consistency in data representation and correct this entry, we have reviewed the raw data source for 1996. This is a noteworthy observation, as the urea application value of 12327 in 2020 was significantly higher than in other years. We have exhaustively examined that particular data point.

3. Disconnection Between Results and Discussion
- The claim that "rice cultivation and soil management have the highest risk" (Abstract) contradicts Figure 1, which shows Group C's rice attitude value as 0.239 (low risk) and Group D as 0.442 (higher), with no resolution of this contradiction.
- Policy recommendations (e.g., "Decoupling direct payments can enhance food security," Sec 6) are not derived from empirical results but merely repeat literature views (e.g., [20]).
- Insufficient Explanation of Key Figures:
- The meanings of "!" and "×" symbols in Figures 1-3 (Sec 4.1) are unclear; Figures 4-6 focus on crop yield trends rather than CO_2 emission reduction effects, deviating from the research objective.

Reply: Thank you very much for your valuable comments and suggestions. According to your valued comments, we have revised it carefully as follows.

The heading 4.1 (first and second paragraph) of figure 1 has been entirely updated and revised to include a comprehensive description of the rise and soil, featuring a variety of marks. Additionally, each group has conducted an individual analysis and interpretation of the estimated results.

Revised and updated the final paragraph of heading 4.2 to improve food security and decouple direct payment.

The first paragraph of the fourth heading has been revised and updated with a full symbol that has a distinct meaning (the meanings of the "!" and "×" symbols) explanation. This symbol is subsequently used in the interpretation of the results and the overall interpretation of the headings 4.2 and 4.3.

4. Literature Citation and Academic Rigor
- Excessive Self-Citation: References by the corresponding author, Nawaz Khan, account for 30% of the references (e.g., [31-33,35,37-38,45,77]), many of which are unrelated to Turkish agriculture, suggesting citation bias.
- Ignoring Key Literature in the Field: The FAO CSA framework and Turkish local studies (e.g., the Turkish Ministry of Agriculture's National Climate Smart Agriculture Strategy) are not cited.

Reply: Thank you very much for your valuable comments and suggestions. According to your valued comments, we have revised it carefully as follows.

Thank you for your feedback regarding the self-citations by the corresponding author, Nawaz Khan, and for clarifying the rationale behind their inclusion. We also note that and have already updated, revised, and modified the references. We appreciate this proactive step to ensure the reference list is both comprehensive in reflecting foundational work on decoupling and appropriately refined for the current manuscript. We have ensure this context is reflected in our understanding of the manuscript's referencing.

Thank you for highlighting the omission of key literature, specifically the FAO Climate-Smart Agriculture (CLS-A) framework and relevant Turkish local studies, such as the Turkish Ministry of Agriculture's National Climate Smart Agriculture Strategy.

Updated and modified heading 2.1 and also we acknowledge the importance of these foundational documents and agree that their inclusion is crucial for contextualizing our research within the broader field and local policy landscape. We have revised the manuscript to incorporate these essential citations, ensuring that our work is appropriately situated within the established academic and policy discourse on CLS-A.

REVIEWER #2:

Reviewer #2: 1. My main comments are mentioned on the manuscript PDF file.
2. Abstract: The abstract provides a concise overview of the study, highlighting the main objectives and significant findings. The abstract could benefit from a brief mention of the broader implications of these findings for different crop production.

Reply: The synopsis section has been updated and modified in accordance with the instructions provided to reflect the primary content of the study.

3. Introduction: Minimize repetitive statements, focus more on the novelty of the study, reducing excessive detail on general information.

Reply: The entire introduction portion (heading 1) has been revised in accordance with the provided instructions, with a particular emphasis on the first to fourth paragraphs. This revision emphasizes the primary content of the topic and further clarifies the novelty of the study. Additionally, the literature section has been revised and updated in headings 2.1 and 2.2.

4. Methodology: Elaborate the methodology to make it easy to understand. Make it more logical and robust.

Reply: The entire methodology has been revised in accordance with the instructions provided, with the first paragraph of heading 3.1 being entirely modified and updated to include the detailed steps of portfolio computations. Additionally, the decoupling and portfolio approach are clearly explained in the updated paragraph's first paragraph, as well as in heading 3.2 and heading 3.3.

5. Data Interpretation in Results: The results section presents extensive data but lacks in-depth interpretation and discussion of these findings within the section, missing an opportunity to provide insights into the implications of these results.

Reply: The results section of heading 4 has been completely updated and modified, as well as the initial paragraph of heading 4. The interpretation and discussion are explicitly explained in the first and second paragraphs of heading 4.1, as well as the final paragraph of heading 4.2, which has been updated and revised.

6. Conclusion: The conclusion succinctly summarizes the findings. But it could be enhanced by providing specific recommendations for future research.

Reply: Additionally, specific recommendations for future study have been included in the first and second paragraphs of Heading 6, which have been edited and amended. The third paragraph has also been modified in terms of policy recommendations and ongoing research.

In summary, while the manuscript presents a valuable study with significant implications for the Climate Smart Agriculture in Turkey, addressing these critical flaws would strengthen the manuscript's contribution to the field.

Reply: Thank you for your comprehensive review and valuable feedback on our manuscript. We truly appreciate your insightful comments, particularly your recognition of the study's potential contribution to Climate-Smart Agriculture in Turkey. We fully understand and agree that addressing the critical flaws you have identified is essential to strengthen the manuscript's overall impact and contribution to the field. Therefoe, the entire mansucript have been revised and updated accordingly. We are committed to thoroughly revising the manuscript based on your detailed suggestions to ensure it meets the highest standards of academic rigor and clarity.

REVIEWER #3:

Reviewer #3: This manuscript addresses an important topic at the intersection of climate change, agriculture, and sustainability in Turkey. The study argues that adopting sustainable CSA practices can support Turkey’s transition to a low-carbon economy while ensuring long-term agricultural productivity and resilience to climate change. Please check for suggestions and recommendations in the attachment.

Dear Editor,

I am writing to provide my review of the manuscript titled “Exploring Climate Smart Agriculture in Turkey: Enhancing Food Security and Sustainable Practices for the Reduction of CO_2 Emissions”. The manuscript explores the role of Climate Smart Agriculture (CSA) in reducing CO_2 emissions and enhancing food security in Turkey. It applies a "decoupling" methodology, typically used in economics, to analyze how agricultural practices like rice cultivation, soil management, and manure application influence environmental outcomes. The study argues that adopting sustainable CSA practices can support Turkey’s transition to a low-carbon economy while ensuring long-term agricultural productivity and resilience to climate change.

While the manuscript introduces a technically innovative framework with promising results, I believe that several revisions could improve its clarity, interpretability, and impact. Below, I offer my detailed comments and recommendations:

Abstract:

- The abstract lacks grammatical coherence and reads as a fragmented set of ideas.
- Terms like "portfolio returns" are used without context or clarity.
- It is unclear how the decoupling method connects concretely with food security outcomes.
- Use a structured abstract (Background, Methods, Results, Conclusion) if permitted by the journal.
- Clarify the methodology in one sentence, avoiding jargon unless defined.
- Quantitative results (e.g., "1% variation in rice cultivation...") should be explained in lay terms.

Thank you for your thorough review and valuable feedback on our abstract. We appreciate your insightful comments, which will significantly help us improve the clarity and impact of our submission.

We have carefully considered each point raised and will revise the abstract as follows:

- **Grammatical Coherence and Fragmentation:** We acknowledge the abstract's current lack of flow and fragmented ideas. We have meticulously revise the abstract to ensure grammatical coherence, improve sentence structure, and create a smoother, more logical progression of ideas.
- **Undefined Terms (e.g., "portfolio returns"):** We have updated and rvised and we recognize the need for better contextualization. We will clarify all specific terms like "portfolio returns" by providing brief definitions or explanations within the abstract, ensuring they are understandable to a broader audience without prior context.
- **Connection of Decoupling Method to Food Security:** We agree that this connection needs to be more explicit. We have modified that issues more clearly in section 1 and 5 and clearly articulate how our decoupling method directly relates to and informs food security outcomes, explaining the underlying mechanism or link. Especially in methoadlolgy portion have been revised and updated.
- **Structured Abstract:** We have revised and updated the whole abstract and double check

journal's guidelines regarding abstract structure. If permitted, we have revise the abstract to follow a **structured format (Background, Methods, Results, Conclusion)** to enhance clarity and readability.

- **Methodology Clarity:** We have refined the methodology description to a single, concise sentence that explains our approach in straightforward terms, avoiding jargon where possible or ensuring any necessary technical terms are immediately defined.
- **Explanation of Quantitative Results:** Modified and updated. We have ensured that all quantitative results, such as "1% variation in rice cultivation," are explained in **lay terms**, focusing on their practical implications and significance rather than just presenting raw numbers.

We are confident that these revisions will significantly strengthen our abstract and address all your concerns.

Introduction:

- Some content is repeated or redundant (e.g., definition of CLS-A).
  Updated and revised the whole manuscript with repeated or redundant sentences. I have modified the repeated phrases, definitions, or explanations, especially the example "CLS-A" and modified and revised the additionally paragrpah in heading 1.
- The narrative is somewhat disjointed; transitions between ideas (e.g., agroforestry, decoupling, direct payments) need smoothing.
  I have updated and revised mansucript on smoothing the connections between different ideas like climate impacts, the need for CSA, decoupling, and direct payments, ensuring a logical progression. Updated and revised heading 1 (first and second paragrpah and forth paragraph). Regarding decoupling, updated and revised heading 2 (heading 2.1, and 2.2) , 3 (3.1, 3.2 and 3.3) and 4 (4.1, 4.2 and so on)
- Some references are cited incorrectly or not at all when needed.
  Updated and revised and entirely double checked the mansucript and put the right reference on right place and proper citied according the context of mansucript.
- Begin with a clear definition of Climate-Smart Agriculture (CSA), followed by its relevance to Turkey.
  Updated and revised in heading 1 and include second paragprah with clear content of CSA, which is in the study CLS-A.
- Present a logical flow from climate impacts → need for CSA → decoupling as a proposed method.
- Updated and revised
- Condense and eliminate repetitions.
  The whole manuscitp have been revised and updated and condense the eliminate repitation of sentences.

Methodology:

- The methodology mixes economic portfolio modeling with agricultural and environmental data, but the rationale is underdeveloped.
  Thank you for your valuable feedback regarding the underdeveloped rationale for the methodology. I agree that a clearer explanation of *why* decoupling techniques were employed with portfolio return is crucial.

  Updated and revised the heading 3.1 (first two paragraph) heading 3.2 (first paragrpah) heading 3.3 (first paragrpah) due to clear the concept of portfolio return. The core of our approach lies in decoupling the drivers of portfolio return in the context of agricultural and environmental factors. Specifically, isolate the impact of Environmental/Agricultural Shocks, attribute Performance to Specific Factors and Enhance Risk Management and Investment Strategy. Essentially, while economic portfolio modeling provides the framework for risk and return optimization, the decoupling techniques allow us to disentangle the intricate web of influences stemming from agricultural and environmental dynamics on those returns.

- The use of terms like "portfolio returns," "weighted risk," and "beta decoupling" seems adapted from finance, which may not be appropriate or needs clearer justification.
  The first paragraph of heading 3.1 has been updated and altered, and the reason has been provided using three separate points and arguments. When compared to a simple aggregated view, this offers a more comprehensive and actionable understanding, which in turn helps to inform investment decisions that are more resilient and sustainable.

- Data sources are described but not well contextualized.
  The heading 3.2 has been updated and amended, and it has been contextualized.

- Clarify why financial modeling (portfolio theory) is an appropriate method for evaluating CO_2 emissions and agriculture.
  In order to clarify the idea of portfolio return, the headings 3.1 (the first two paragraphs), 3.2 (the first paragraph), and 3.3 (the first paragraph) have been updated and changed. Specially first paragraph of heading 3.1, the three main point which shows the separating the impact of agricultural and environmental variables on portfolio return is central to our strategy with financial modeling.

- Provide equations and define all technical terms.

The technical term "portfolio return" has been incorporated into the new header 3.4. We appreciate your request for additional information regarding the technical terms and equations employed in the analysis. This section delineates the methodologies employed to determine the "Average Return" and "Total Risk" for each agricultural management strategy across various groups and under variable aeration conditions.

- Use a separate subsection for "Data Description" and ensure variables are explicitly listed.

I appreciate your useful review. In accordance with your favorable instructions, we have revised heading 3.2 and distributed the data description, which includes all individual predictor variables.

Results:

- Figures are referenced (e.g., Fig. 1, 2, 3); ensure they are clear and meaningful.
  I have updated and corrected this, and I am extremely grateful for your wonderful review.
- The narrative is hard to follow due to inconsistent use of terminology and a lack of interpretation.
  The section 4 (heading 4) has been updated and revised in accordance with the instructions provided, as well as the methodology of section 3. Additionally, the first and second paragraphs of heading 4.1 have been revised. The final paragraph of heading 4.2 has also been revised and altered.
- Statistical significance is not mentioned.
  The individual change in percentage was calculated in the same manner as the portfolio return during the decoupling process. Throughout this process, we utilized statistical analysis to determine the difference in change between the various groups (heading 4). Direct regression is not implemented in accordance with the decoupling methodology (section 3), as decoupling is computed according to prior research, and portfolio returns are subsequently computed on that basis.
- Describe trends clearly: What does a 1% change mean in practice?
  The section 3 has been altered in accordance with the 1% modification, particularly heading 3.1 (third paragraph), as well as heading 4's first two paragraphs.
- Present tables/figures with meaningful comparisons (e.g., between groups or time periods).
  A thorough review and revision have been performed on each and all tables and figures.
- Use subheadings like "High-Risk Zones," "Low-Risk Zones," etc., more strategically.
  Heading 4.1 and 4.2 have been updated and revised in first two and last paragraphs

Discussion:

- Points about food security, technology, and yield improvement are scattered.
  Updated and revised heading 5.
- Summarize key findings.
- Updated and revised heading 5 especially in 1 and 2nd paragraphs with key finding.
- Compare your results with other studies (discuss agreement or deviation).
  According to above results the entire portion of discussion have been updated and revised.
- Explicitly connect the findings to policy recommendations for Turkey.
  Updated and revised heading 5, third paragraph

Conclusion:

- The conclusion repeats earlier points without adding synthesis.
  Updated and revised heading 6 and modified according to results.
- Multiple objectives are listed without prioritization.
  According to given objectives of the study the conclusion portion have been revised and updated.
- Reframe into a concise summary of key insights, followed by clear and feasible recommendations.
  Updated and revised second and third paragraph of heading 6.
- Avoid new information not discussed in earlier sections.
  We appreciate your careful review and comment. Our intention in the conclusion section was to provide a synthesis of the key findings discussed in the results and discussion sections, offering a broader perspective on their implications. While no new empirical data has been introduced, we have elaborated on the *implications* of our previously presented results. We have now rephrased these sentences to more explicitly link back to the findings discussed earlier, ensuring they are clearly presented as interpretations derived directly from the preceding sections rather than new information in heading 6.
- Suggest future research directions more explicitly.
  We value your assistance in clarifying the trajectory of our future research. Our initial objective was to offer a comprehensive overview; however, we recognize the advantages of greater specificity. The Conclusion has been updated to incorporate more specific and actionable recommendations for future research, such as the third and fourth paragraphs of heading 6. We are of the opinion that these modifications improve the practical utility of our research for other researchers.

References:

- Ensure all references are recent, peer-reviewed, and appropriately cited (e.g., ref. number 7).
  We are grateful for your comprehensive evaluation of our references. We have conducted a thorough update, replacing older sources with more recent, peer-reviewed publications as needed. Additionally, we have meticulously reviewed all citations for accuracy and proper formatting, with a particular emphasis on the entire reference's numbers.
- Eliminate non-scholarly sources unless critically justified.
  I appreciate your input. We have conducted a comprehensive review of our reference list to identify any non-scholarly sources, and the entire manuscript has been updated and revised from section 1 to 6.
- Ensure that all references cited in the text appear in the reference list and vice versa.
  I appreciate your attention to this matter. We have conducted a thorough cross-reference of all in-text citations with our reference list. We have verified that the reference list contains every source cited in the manuscript, and conversely, each entry in the reference list is cited at least once in the text.
- To enhance the currency and relevance of the review, prioritize sources from 2017 to 2025 where applicable.

I appreciate your insightful recommendation. We concur that the currency and relevance of our review will be improved by incorporating more recent literature. We have comprehensively revised entire manuscript references, prioritizing sources published between 2017 and 2025 whenever applicable, while preserving foundational works that are essential to the subject.

- Remove outdated references from methodological sections, unless foundational.

  Your precise comment regarding the currency of references in our methodological sections is greatly appreciated. We have conducted a comprehensive update, replacing obsolete citations with more recent and pertinent sources as needed. We have meticulously verified that any older references that have been retained are indeed foundational to the methodologies discussed, providing essential historical context or critical backdrop.

REVIEWER #4:

Reviewer #4: Manuscript ID: PONE-D-25-27665
Title: Exploring Climate Smart Agriculture in Turkey: Enhancing Food Security and Sustainable Practices for the Reduction of CO_2 Emissions
The subject is relevant but the presentation of the manuscript is very weak, for instance, methodology has been written weakly without enough explanations, the tables and figures have been prepared with insufficient attention, etc. Therefore, I cannot unfortunately recommend this manuscript for publication, while can encourage the authors for applying improvements.

We are grateful for your comprehensive evaluation, sir your constructive feedback and comprehensive evaluation of our manuscript are greatly appreciated. Our gratitude for your recognition of the subject's importance is greatly appreciated. Our sincerest apologies for the inadequate presentation of our manuscript, particularly in the methodology section, and for the insufficient attention given to the tables and figures. We acknowledge that the clarity and intelligibility of our work were significantly affected by these deficiencies.

We have since implemented a thorough revision of the entire manuscript, spanning from Section 1 to Section 6. This revision has been comprehensive, with a particular emphasis on addressing the deficiencies you identified:

- Methodology: The methodology section has been significantly revised and expanded to provide a more detailed and explicit explanation of our experimental design, procedures, and analytical approaches in headings 3.1, 3.2, 3.3, and 3.4. We are confident that this improved lucidity will enable readers to comprehend and replicate our work to the fullest.
- Tables and Figures: We have meticulously reviewed and regenerated all tables and figures in accordance with the methodology, ensuring that they are presented in a professional manner and are clear. Furthermore, Figures 1 and 3 are essentially tables; however, they were converted to figures due to the use of different symbols. Due to the confusing nature of the portfolio return concept when represented by various symbols in a table, we have opted to employ a figure in place of a table. Additionally, they are now equipped with clear labels, exhaustive legends, and are intended to effectively communicate the data without any ambiguity.

- General Presentation: In addition to these specific points, we have conducted a comprehensive review of the entire manuscript to ensure compliance with academic standards, including language and formatting, coherence, and clarity (Sections 1 to 6 have been updated and revised). We are confident that the quality and presentation of the manuscript have been substantially enhanced by these extensive revisions, which have addressed all of the concerns you addressed. We trust that the manuscript has been improved to a level that is more suitable for publication.

Finally, thank you so much for your helpful advice and invaluable ostentation. I'm speechless in the face of your vast knowledge and impressive list of publications, but I can attest to the fact that this manuscript benefits greatly from your fertile ostentation.

Once again, I greatly appreciate your insightful feedback and recommendations.
