# Supplementary material for: Exploring climate smart agriculture in Turkey: Enhancing food security and sustainable practices for the reduction of CO₂ emissions
Source: PLoS One. 2026 Mar 31;21(3):e0344924. doi: 10.1371/journal.pone.0344924 (PMC13037976; doi:10.1371/journal.pone.0344924)
Supplement: Attachment — Submitted filename: PONE-D-25-27665.docx [file pone.0344924.s005.docx]

Dear Editor,

I am writing to provide my review of the manuscript titled “Exploring Climate Smart Agriculture in Turkey: Enhancing Food Security and Sustainable Practices for the Reduction of CO₂ Emissions”. The manuscript explores the role of Climate Smart Agriculture (CSA) in reducing CO₂ emissions and enhancing food security in Turkey. It applies a "decoupling" methodology, typically used in economics, to analyze how agricultural practices like rice cultivation, soil management, and manure application influence environmental outcomes. The study argues that adopting sustainable CSA practices can support Turkey’s transition to a low-carbon economy while ensuring long-term agricultural productivity and resilience to climate change.

While the manuscript introduces a technically innovative framework with promising results, I believe that several revisions could improve its clarity, interpretability, and impact. Below, I offer my detailed comments and recommendations:

1. **Abstract:**

- The abstract lacks grammatical coherence and reads as a fragmented set of ideas.
- Terms like "portfolio returns" are used without context or clarity.
- It is unclear how the decoupling method connects concretely with food security outcomes.
- Use a structured abstract (Background, Methods, Results, Conclusion) if permitted by the journal.
- Clarify the methodology in one sentence, avoiding jargon unless defined.
- Quantitative results (e.g., "1% variation in rice cultivation...") should be explained in lay terms.

2. **Introduction:**

- Some content is repeated or redundant (e.g., definition of CLS-A).
- The narrative is somewhat disjointed; transitions between ideas (e.g., agroforestry, decoupling, direct payments) need smoothing.
- Some references are cited incorrectly or not at all when needed.
- Begin with a clear definition of Climate-Smart Agriculture (CSA), followed by its relevance to Turkey.
- Present a logical flow from climate impacts → need for CSA → decoupling as a proposed method.
- Condense and eliminate repetitions.

3. **Methodology:**

- The methodology mixes economic portfolio modeling with agricultural and environmental data, but the rationale is underdeveloped.
- The use of terms like "portfolio returns," "weighted risk," and "beta decoupling" seems adapted from finance, which may not be appropriate or needs clearer justification.
- Data sources are described but not well contextualized.
- Clarify why financial modeling (portfolio theory) is an appropriate method for evaluating CO₂ emissions and agriculture.
- Provide equations and define all technical terms.
- Use a separate subsection for "Data Description" and ensure variables are explicitly listed.

4. **Results:**

- Figures are referenced (e.g., Fig. 1, 2, 3); ensure they are clear and meaningful.
- The narrative is hard to follow due to inconsistent use of terminology and a lack of interpretation.
- Statistical significance is not mentioned.
- Describe trends clearly: What does a 1% change mean in practice?
- Present tables/figures with meaningful comparisons (e.g., between groups or time periods).
- Use subheadings like "High-Risk Zones," "Low-Risk Zones," etc., more strategically.

5. **Discussion:**

- Points about food security, technology, and yield improvement are scattered.
- Summarize key findings.
- Compare your results with other studies (discuss agreement or deviation).
- Explicitly connect the findings to policy recommendations for Turkey.

6. **Conclusion:**

- The conclusion repeats earlier points without adding synthesis.
- Multiple objectives are listed without prioritization.
- Reframe into a concise summary of key insights, followed by clear and feasible recommendations.
- Avoid new information not discussed in earlier sections.
- Suggest future research directions more explicitly.

7. **References:**

- Ensure all references are recent, peer-reviewed, and appropriately cited (e.g., ref. number 7).
- Eliminate non-scholarly sources unless critically justified.
- Ensure that all references cited in the text appear in the reference list and vice versa.
- To enhance the currency and relevance of the review, prioritize sources from 2017 to 2025 where applicable.
- Remove outdated references from methodological sections, unless foundational.
