# Supplementary material for: Exploring climate smart agriculture in Turkey: Enhancing food security and sustainable practices for the reduction of CO₂ emissions
Source: PLoS One. 2026 Mar 31;21(3):e0344924. doi: 10.1371/journal.pone.0344924 (PMC13037976; doi:10.1371/journal.pone.0344924)
Supplement: Attachment — Submitted filename: Response to Reviewers 2025-07-31.docx [file pone.0344924.s006.docx]

**Authors' Response to Reviewers' Comments**

| **Subject:** | PLOS ONE Decision: Revision required [PONE-D-25-27665] |
| --- | --- |

Dear Dr. Khan,

PONE-D-25-27665

Title: **Exploring Climate Smart Agriculture in Turkey: Enhancing Food Security and Sustainable Practices for the Reduction of CO2 Emissions**

Dear editor and reviewers,

Thank you very much for your letter and reviewers’ valuable suggestions on our manuscript entitled “**Exploring Climate Smart Agriculture in Turkey: Enhancing Food Security and Sustainable Practices for the Reduction of CO2 Emissions”** No: [PONE-D-25-27665]. Those comments are precious and helpful for improving our paper and providing the essential guiding significance to our research. We have carefully checked and improved the manuscript according to the editor’s and reviewers’ comments.

**REVIEWER #1:**

**Reviewer #1: This study explores the role of Climate Smart Agriculture (CSA) in Turkey for food security and CO₂ emission reduction, which is a topic of practical significance. However, the manuscript has significant methodological flaws, conceptual confusion, and issues with data interpretation, severely affecting the reliability of the conclusions.**

1. Conceptual and Terminological Confusion
- Misuse of Key Terms: The manuscript consistently fails to clearly define the core dimensions of CSA (mitigation, adaptation, increased productivity).
- Misapplication of Decoupling Concept:
- Decoupling should measure the decoupling of economic growth from carbon emissions (e.g., using Tapio's decoupling index), but the manuscript forcibly links it to financial indicators such as "portfolio return" (Abstract, Sec 3.1), lacking theoretical basis.
- For example: "The decoupling process is responsible for estimating the percentage change in portfolio returns" (Abstract) — there is a missing logical connection between environmental science and financial models.

**Reply:** I appreciate your assistance in supplying the specified text from the Canvas. We comprehend that you would like a response to the statement concerning the conceptual and terminological confusion in the manuscript.

The following is a response that addresses the concerns that were raised:

" We appreciate the comprehensive feedback you provided regarding the conceptual and terminological clarity of the manuscript." We acknowledge the arguments concerning:

It is imperative that the fundamental dimensions of Climate-Smart Agriculture (CSA), which are specifically mitigation, adaptation, and increased productivity, be defined in a more explicit and precise manner. We concur with this proposition. In order to guarantee consistent application throughout the text and incorporate precise definitions, we will revise the manuscript (Heading 1, 1st paragraph).

Regarding the decoupling concept, we comprehend your apprehension regarding the coerced connection between 'decoupling' and financial indicators, such as 'portfolio return.' In this study, we first estimated the decoupling and then portfolio return, focusing on its relationship to carbon emissions or other relevant environmental metrics, in accordance with established environmental science principles. We acknowledge that the standard definition of decoupling is related to economic growth and carbon emissions by decoupling and portfolio return. In conclusion, the abstract section has been revised and updated.

2. Methodological Deficiencies
- The use of "portfolio weighted risk return" (Sec 3.3) to analyze agricultural emissions is not justified. The causal relationship between financial indicators (such as beta values, risk premiums) and CO₂ emissions is not argued.
- Ambiguous grouping criteria: Turkey is divided into Groups A-D (Sec 4.1), but the criteria for grouping (geographical? Economic level?) are not explained.
- Data Handling Issues:
- Data sources are only mentioned as "World Bank" (Sec 3.2), without providing specific datasets or access links, violating PLOS data policy (Page 4-5).
- Anomalies in Appendix A tables (e.g., in 1996, the "Agriculture total (N)" column shows 100/52297; in 2020, urea application value 12327 is much higher than other years) are not explained.

**Reply:** Thank you very much for your valuable comments and suggestions. According to your valued comments, we have revised it carefully as follows.

There has been an update and revision made to heading 3.3 in accordance with the casual relationship that exists between the financial indicators in the first paragraph.

Your insightful commentary regarding the ambiguity of the classification criteria (Groups A-D) in this manuscript is greatly appreciated. You are correct that the document does not expressly define the criteria for these groups (A, B, C, D) nor does it specify that they are divided by specific years (e.g., 1992-2023). The decoupling operation is divided into four distinct sections (A–D) for the purpose of analysis in the first paragraph of heading 3.2. It has been updated and revised. The territory of Turkey is not defined, as we only distributed the number of years into four groups, despite the fact that all of the subsets cover a complete five years. The analysis has interpreted these groupings based on general possibilities, including varying farm types, regions, management strategies, and socio-economic contexts.

We are grateful for your feedback on the data handling and the mention of "World Bank" as a data source without specific links, which you identified as a potential violation of PLOS data policy. We are grateful for your highlighting of this critical point in heading 3.2. We have updated and revised our analysis to reflect that it did indeed use datasets from both the **World Bank (WB)** and **Our Data in the World**. All of the subsets analyzed were analyzed over a full five-year period, and the analysis was conducted using panel data. Detailed information about the precise datasets used, including their specific names and direct access links, is comprehensively provided in **Appendix B** of the manuscript. We are committed to ensuring that this appendix is updated to meet the highest standards of data transparency and reproducibility.

We are grateful for your identification of these particular data management concerns. Revised and updated heading 3.2 (last lines of the first paragraph). To assure consistency in data representation and correct this entry, we have reviewed the raw data source for 1996. This is a noteworthy observation, as the urea application value of 12327 in 2020 was significantly higher than in other years. We have exhaustively examined that particular data point.

3. Disconnection Between Results and Discussion
- The claim that "rice cultivation and soil management have the highest risk" (Abstract) contradicts Figure 1, which shows Group C's rice attitude value as 0.239 (low risk) and Group D as 0.442 (higher), with no resolution of this contradiction.
- Policy recommendations (e.g., "Decoupling direct payments can enhance food security," Sec 6) are not derived from empirical results but merely repeat literature views (e.g., [20]).
- Insufficient Explanation of Key Figures:
- The meanings of "!" and "×" symbols in Figures 1-3 (Sec 4.1) are unclear; Figures 4-6 focus on crop yield trends rather than CO₂ emission reduction effects, deviating from the research objective.

**Reply:** Thank you very much for your valuable comments and suggestions. According to your valued comments, we have revised it carefully as follows.

The heading 4.1 (first and second paragraph) of figure 1 has been entirely updated and revised to include a comprehensive description of the rise and soil, featuring a variety of marks. Additionally, each group has conducted an individual analysis and interpretation of the estimated results.

Revised and updated the final paragraph of heading 4.2 to improve food security and decouple direct payment.

The first paragraph of the fourth heading has been revised and updated with a full symbol that has a distinct meaning (the meanings of the "!" and "×" symbols) explanation. This symbol is subsequently used in the interpretation of the results and the overall interpretation of the headings 4.2 and 4.3.

4. Literature Citation and Academic Rigor
- Excessive Self-Citation: References by the corresponding author, Nawaz Khan, account for 30% of the references (e.g., [31-33,35,37-38,45,77]), many of which are unrelated to Turkish agriculture, suggesting citation bias.
- Ignoring Key Literature in the Field: The FAO CSA framework and Turkish local studies (e.g., the Turkish Ministry of Agriculture's National Climate Smart Agriculture Strategy) are not cited.

**Reply:** Thank you very much for your valuable comments and suggestions. According to your valued comments, we have revised it carefully as follows.

Thank you for your feedback regarding the self-citations by the corresponding author, Nawaz Khan, and for clarifying the rationale behind their inclusion. We also note that and have already updated, revised, and modified the references. We appreciate this proactive step to ensure the reference list is both comprehensive in reflecting foundational work on decoupling and appropriately refined for the current manuscript. We have ensure this context is reflected in our understanding of the manuscript's referencing.

Thank you for highlighting the omission of key literature, specifically the FAO Climate-Smart Agriculture (CLS-A) framework and relevant Turkish local studies, such as the Turkish Ministry of Agriculture's National Climate Smart Agriculture Strategy.

Updated and modified heading 2.1 and also we acknowledge the importance of these foundational documents and agree that their inclusion is crucial for contextualizing our research within the broader field and local policy landscape. We have revised the manuscript to incorporate these essential citations, ensuring that our work is appropriately situated within the established academic and policy discourse on CLS-A.

**REVIEWER #2:**

Reviewer #2: 1. My main comments are mentioned on the manuscript PDF file.
2. Abstract: The abstract provides a concise overview of the study, highlighting the main objectives and significant findings. The abstract could benefit from a brief mention of the broader implications of these findings for different crop production.

**Reply:** The synopsis section has been updated and modified in accordance with the instructions provided to reflect the primary content of the study.

3. Introduction: Minimize repetitive statements, focus more on the novelty of the study, reducing excessive detail on general information.

**Reply:** The entire introduction portion (heading 1) has been revised in accordance with the provided instructions, with a particular emphasis on the first to fourth paragraphs. This revision emphasizes the primary content of the topic and further clarifies the novelty of the study. Additionally, the literature section has been revised and updated in headings 2.1 and 2.2.

4. Methodology: Elaborate the methodology to make it easy to understand. Make it more logical and robust.

**Reply:** The entire methodology has been revised in accordance with the instructions provided, with the first paragraph of heading 3.1 being entirely modified and updated to include the detailed steps of portfolio computations. Additionally, the decoupling and portfolio approach are clearly explained in the updated paragraph's first paragraph, as well as in heading 3.2 and heading 3.3.

5. Data Interpretation in Results: The results section presents extensive data but lacks in-depth interpretation and discussion of these findings within the section, missing an opportunity to provide insights into the implications of these results.

**Reply:** The results section of heading 4 has been completely updated and modified, as well as the initial paragraph of heading 4. The interpretation and discussion are explicitly explained in the first and second paragraphs of heading 4.1, as well as the final paragraph of heading 4.2, which has been updated and revised.

6. Conclusion: The conclusion succinctly summarizes the findings. But it could be enhanced by providing specific recommendations for future research.

**Reply:** Additionally, specific recommendations for future study have been included in the first and second paragraphs of Heading 6, which have been edited and amended. The third paragraph has also been modified in terms of policy recommendations and ongoing research.

In summary, while the manuscript presents a valuable study with significant implications for the Climate Smart Agriculture in Turkey, addressing these critical flaws would strengthen the manuscript's contribution to the field.

**Reply:** Thank you for your comprehensive review and valuable feedback on our manuscript. We truly appreciate your insightful comments, particularly your recognition of the study's potential contribution to Climate-Smart Agriculture in Turkey. We fully understand and agree that addressing the critical flaws you have identified is essential to strengthen the manuscript's overall impact and contribution to the field. Therefoe, the entire mansucript have been revised and updated accordinally. We are committed to thoroughly revising the manuscript based on your detailed suggestions to ensure it meets the highest standards of academic rigor and clarity.

**REVIEWER #3:**

Reviewer #3: This manuscript addresses an important topic at the intersection of climate change, agriculture, and sustainability in Turkey. The study argues that adopting sustainable CSA practices can support Turkey’s transition to a low-carbon economy while ensuring long-term agricultural productivity and resilience to climate change. Please check for suggestions and recommendations in the attachment.

Dear Editor,

I am writing to provide my review of the manuscript titled “Exploring Climate Smart Agriculture in Turkey: Enhancing Food Security and Sustainable Practices for the Reduction of CO₂ Emissions”. The manuscript explores the role of Climate Smart Agriculture (CSA) in reducing CO₂ emissions and enhancing food security in Turkey. It applies a "decoupling" methodology, typically used in economics, to analyze how agricultural practices like rice cultivation, soil management, and manure application influence environmental outcomes. The study argues that adopting sustainable CSA practices can support Turkey’s transition to a low-carbon economy while ensuring long-term agricultural productivity and resilience to climate change.

While the manuscript introduces a technically innovative framework with promising results, I believe that several revisions could improve its clarity, interpretability, and impact. Below, I offer my detailed comments and recommendations:

Abstract:

- The abstract lacks grammatical coherence and reads as a fragmented set of ideas.
- Terms like "portfolio returns" are used without context or clarity.
- It is unclear how the decoupling method connects concretely with food security outcomes.
- Use a structured abstract (Background, Methods, Results, Conclusion) if permitted by the journal.
- Clarify the methodology in one sentence, avoiding jargon unless defined.
- Quantitative results (e.g., "1% variation in rice cultivation...") should be explained in lay terms.

Thank you for your thorough review and valuable feedback on our abstract. We appreciate your insightful comments, which will significantly help us improve the clarity and impact of our submission.

We have carefully considered each point raised and will revise the abstract as follows:

- **Grammatical Coherence and Fragmentation:** We acknowledge the abstract's current lack of flow and fragmented ideas. We have meticulously revise the abstract to ensure grammatical coherence, improve sentence structure, and create a smoother, more logical progression of ideas.
- **Undefined Terms (e.g., "portfolio returns"):** We have updated and rvised and we recognize the need for better contextualization. We will clarify all specific terms like "portfolio returns" by providing brief definitions or explanations within the abstract, ensuring they are understandable to a broader audience without prior context.
- **Connection of Decoupling Method to Food Security:** We agree that this connection needs to be more explicit. We have modified that issues more clearly in section 1 and 5 and clearly articulate how our decoupling method directly relates to and informs food security outcomes, explaining the underlying mechanism or link. Especially in methoadlolgy portion have been revised and updated.
- **Structured Abstract:** We have revised and updated the whole abstract and double check

journal's guidelines regarding abstract structure. If permitted, we have revise the abstract to follow a **structured format (Background, Methods, Results, Conclusion)** to enhance clarity and readability.

- **Methodology Clarity:** We have refined the methodology description to a single, concise sentence that explains our approach in straightforward terms, avoiding jargon where possible or ensuring any necessary technical terms are immediately defined.
- **Explanation of Quantitative Results:** Modified and updated. We have ensured that all quantitative results, such as "1% variation in rice cultivation," are explained in **lay terms**, focusing on their practical implications and significance rather than just presenting raw numbers.

We are confident that these revisions will significantly strengthen our abstract and address all your concerns.

Introduction:

- Some content is repeated or redundant (e.g., definition of CLS-A).

Updated and revised the whole manuscript with repeated or redundant sentences. I have modified the repeated phrases, definitions, or explanations, especially the example "CLS-A" and modified and revised the additionally paragrpah in heading 1.

- The narrative is somewhat disjointed; transitions between ideas (e.g., agroforestry, decoupling, direct payments) need smoothing.

I have updated and revised mansucript on smoothing the connections between different ideas like climate impacts, the need for CSA, decoupling, and direct payments, ensuring a logical progression. Updated and revised heading 1 (first and second paragrpah and forth paragraph). Regarding decoupling, updated and revised heading 2 (heading 2.1, and 2.2) , 3 (3.1, 3.2 and 3.3) and 4 (4.1, 4.2 and so on)

- Some references are cited incorrectly or not at all when needed.

Updated and revised and entirely double checked the mansucript and put the right reference on right place and proper citied according the context of mansucript.

- Begin with a clear definition of Climate-Smart Agriculture (CSA), followed by its relevance to Turkey.

Updated and revised in heading 1 and include second paragprah with clear content of CSA, which is in the study CLS-A.

- Present a logical flow from climate impacts → need for CSA → decoupling as a proposed method.
- Updated and revised
- Condense and eliminate repetitions.

The whole manuscitp have been revised and updated and condense the eliminate repitation of sentences.

Methodology:

- The methodology mixes economic portfolio modeling with agricultural and environmental data, but the rationale is underdeveloped.

Thank you for your valuable feedback regarding the underdeveloped rationale for the methodology. I agree that a clearer explanation of *why* decoupling techniques were employed with portfolio return is crucial.

Updated and revised the heading 3.1 (first two paragraph) heading 3.2 ( first paragrpah) heading 3.3 ( first paragrpah ) due to clear the concept of portfolio return. The core of our approach lies in decoupling the drivers of portfolio return in the context of agricultural and environmental factors. Specifically, isolate the impact of Environmental/Agricultural Shocks, attribute Performance to Specific Factors and Enhance Risk Management and Investment Strategy. Essentially, while economic portfolio modeling provides the framework for risk and return optimization, the decoupling techniques allow us to disentangle the intricate web of influences stemming from agricultural and environmental dynamics on those returns.

- The use of terms like "portfolio returns," "weighted risk," and "beta decoupling" seems adapted from finance, which may not be appropriate or needs clearer justification.

The first paragraph of heading 3.1 has been updated and altered, and the reason has been provided using three separate points and arguments. When compared to a simple aggregated view, this offers a more comprehensive and actionable understanding, which in turn helps to inform investment decisions that are more resilient and sustainable.

- Data sources are described but not well contextualized.

The heading 3.2 has been updated and amended, and it has been contextualized.

- Clarify why financial modeling (portfolio theory) is an appropriate method for evaluating CO₂ emissions and agriculture.

In order to clarify the idea of portfolio return, the headings 3.1 (the first two paragraphs), 3.2 (the first paragraph), and 3.3 (the first paragraph) have been updated and changed. Specially first paragraph of heading 3.1, the three main point which shows the separating the impact of agricultural and environmental variables on portfolio return is central to our strategy with financial modeling.

- Provide equations and define all technical terms.

The technical term "portfolio return" has been incorporated into the new header 3.4. We appreciate your request for additional information regarding the technical terms and equations employed in the analysis. This section delineates the methodologies employed to determine the "Average Return" and "Total Risk" for each agricultural management strategy across various groups and under variable aeration conditions.

- Use a separate subsection for "Data Description" and ensure variables are explicitly listed.

I appreciate your useful review. In accordance with your favorable instructions, we have revised heading 3.2 and distributed the data description, which includes all individual predictor variables.

Results:

- Figures are referenced (e.g., Fig. 1, 2, 3); ensure they are clear and meaningful.

I have updated and corrected this, and I am extremely grateful for your wonderful review.

- The narrative is hard to follow due to inconsistent use of terminology and a lack of interpretation.

The section 4 (heading 4) has been updated and revised in accordance with the instructions provided, as well as the methodology of section 3. Additionally, the first and second paragraphs of heading 4.1 have been revised. The final paragraph of heading 4.2 has also been revised and altered.

- Statistical significance is not mentioned.

The individual change in percentage was calculated in the same manner as the portfolio return during the decoupling process. Throughout this process, we utilized statistical analysis to determine the difference in change between the various groups (heading 4). Direct regression is not implemented in accordance with the decoupling methodology (section 3), as decoupling is computed according to prior research, and portfolio returns are subsequently computed on that basis.

- Describe trends clearly: What does a 1% change mean in practice?

The section 3 has been altered in accordance with the 1% modification, particularly heading 3.1 (third paragraph), as well as heading 4's first two paragraphs.

- Present tables/figures with meaningful comparisons (e.g., between groups or time periods).

A thorough review and revision have been performed on each and all tables and figures.

- Use subheadings like "High-Risk Zones," "Low-Risk Zones," etc., more strategically.

Heading 4.1 and 4.2 have been updated and revised in first two and last paragraphs

Discussion:

- Points about food security, technology, and yield improvement are scattered.

Updated and revised heading 5.

- Summarize key findings.
- Updated and revised heading 5 especially in 1 and 2^nd^ paragraphs with key finding.
- Compare your results with other studies (discuss agreement or deviation).

According to above results the entire portion of discussion have been updated and revised.

- Explicitly connect the findings to policy recommendations for Turkey.

Updated and revised heading 5, third paragraph

Conclusion:

- The conclusion repeats earlier points without adding synthesis.

Updated and revised heading 6 and modified according to results.

- Multiple objectives are listed without prioritization.

According to given objectives of the study the conclusion portion have been revised and updated.

- Reframe into a concise summary of key insights, followed by clear and feasible recommendations.

Updated and revised second and third paragraph of heading 6.

- Avoid new information not discussed in earlier sections.

We appreciate your careful review and comment. Our intention in the conclusion section was to provide a synthesis of the key findings discussed in the results and discussion sections, offering a broader perspective on their implications. While no new empirical data has been introduced, we have elaborated on the *implications* of our previously presented results. We have now rephrased these sentences to more explicitly link back to the findings discussed earlier, ensuring they are clearly presented as interpretations derived directly from the preceding sections rather than new information in heading 6.

- Suggest future research directions more explicitly.

We value your assistance in clarifying the trajectory of our future research. Our initial objective was to offer a comprehensive overview; however, we recognize the advantages of greater specificity. The Conclusion has been updated to incorporate more specific and actionable recommendations for future research, such as the third and fourth paragraphs of heading 6. We are of the opinion that these modifications improve the practical utility of our research for other researchers.

References:

- Ensure all references are recent, peer-reviewed, and appropriately cited (e.g., ref. number 7).

We are grateful for your comprehensive evaluation of our references. We have conducted a thorough update, replacing older sources with more recent, peer-reviewed publications as needed. Additionally, we have meticulously reviewed all citations for accuracy and proper formatting, with a particular emphasis on the entire reference’s numbers.

- Eliminate non-scholarly sources unless critically justified.

I appreciate your input. We have conducted a comprehensive review of our reference list to identify any non-scholarly sources, and the entire manuscript has been updated and revised from section 1 to 6.

- Ensure that all references cited in the text appear in the reference list and vice versa.

I appreciate your attention to this matter. We have conducted a thorough cross-reference of all in-text citations with our reference list. We have verified that the reference list contains every source cited in the manuscript, and conversely, each entry in the reference list is cited at least once in the text.

- To enhance the currency and relevance of the review, prioritize sources from 2017 to 2025 where applicable.

I appreciate your insightful recommendation. We concur that the currency and relevance of our review will be improved by incorporating more recent literature. We have comprehensively revised entire manuscript references, prioritizing sources published between 2017 and 2025 whenever applicable, while preserving foundational works that are essential to the subject.

- Remove outdated references from methodological sections, unless foundational.

Your precise comment regarding the currency of references in our methodological sections is greatly appreciated. We have conducted a comprehensive update, replacing obsolete citations with more recent and pertinent sources as needed. We have meticulously verified that any older references that have been retained are indeed foundational to the methodologies discussed, providing essential historical context or critical backdrop.

**REVIEWER #4:**

Reviewer #4: Manuscript ID: PONE-D-25-27665
Title: Exploring Climate Smart Agriculture in Turkey: Enhancing Food Security and Sustainable Practices for the Reduction of CO2 Emissions
The subject is relevant but the presentation of the manuscript is very weak, for instance, methodology has been written weakly without enough explanations, the tables and figures have been prepared with insufficient attention, etc. Therefore, I cannot unfortunately recommend this manuscript for publication, while can encourage the authors for applying improvements.

We are grateful for your comprehensive evaluation, sir your constructive feedback and comprehensive evaluation of our manuscript are greatly appreciated. Our gratitude for your recognition of the subject's importance is greatly appreciated. Our sincerest apologies for the inadequate presentation of our manuscript, particularly in the methodology section, and for the insufficient attention given to the tables and figures. We acknowledge that the clarity and intelligibility of our work were significantly affected by these deficiencies.

We have since implemented a thorough revision of the entire manuscript, spanning from Section 1 to Section 6. This revision has been comprehensive, with a particular emphasis on addressing the deficiencies you identified:

- Methodology: The methodology section has been significantly revised and expanded to provide a more detailed and explicit explanation of our experimental design, procedures, and analytical approaches in headings 3.1, 3.2, 3.3, and 3.4. We are confident that this improved lucidity will enable readers to comprehend and replicate our work to the fullest.
- Tables and Figures: We have meticulously reviewed and regenerated all tables and figures in accordance with the methodology, ensuring that they are presented in a professional manner and are clear. Furthermore, Figures 1 and 3 are essentially tables; however, they were converted to figures due to the use of different symbols. Due to the confusing nature of the portfolio return concept when represented by various symbols in a table, we have opted to employ a figure in place of a table. Additionally, they are now equipped with clear labels, exhaustive legends, and are intended to effectively communicate the data without any ambiguity.
- General Presentation: In addition to these specific points, we have conducted a comprehensive review of the entire manuscript to ensure compliance with academic standards, including language and formatting, coherence, and clarity (Sections 1 to 6 have been updated and revised). We are confident that the quality and presentation of the manuscript have been substantially enhanced by these extensive revisions, which have addressed all of the concerns you addressed. We trust that the manuscript has been improved to a level that is more suitable for publication.

Finally, thank you so much for your helpful advice and invaluable ostentation. I'm speechless in the face of your vast knowledge and impressive list of publications, but I can attest to the fact that this manuscript benefits greatly from your fertile ostentation.

**Once again, I greatly appreciate your insightful feedback and recommendations.**
