# Supplementary material for: Exploring climate smart agriculture in Turkey: Enhancing food security and sustainable practices for the reduction of CO₂ emissions
Source: PLoS One. 2026 Mar 31;21(3):e0344924. doi: 10.1371/journal.pone.0344924 (PMC13037976; doi:10.1371/journal.pone.0344924)
Supplement: Attachment — Submitted filename: Com_ 27.08.docx [file pone.0344924.s007.docx]

 In the introduction, the amount of carbon emission from agriculture has not been mentioned. Is it only related with rice field or other crop production? What is the justification of introducing Climate smart agriculture and how it can be done? More over addition of reports on these points is required. Finally, the goal of the study or object is still unclear in abstract, introduction and in conclusion. Although there is extensive information on decoupling technique to estimate the portfolio returns and analyze their relationship with climate-smart agriculture.

Research Methodology:

1. It is not clear why urea application, manure management, and rice cultivation are separately discussed? Is it only for their origin? (in 3.3. Portfolio Returns)
2. “The second-biggest source of carbon dioxide emissions from Turkish CLS-A, after manure management, accounts for about 25% of the overall emissions” is not supported by the given reference.
3. Overall methodology is still confusing.

The result and discussion are very superficial and general. It needs to be more in-depth and better justify the work conducted.
