# Supplementary material for: Exploring climate smart agriculture in Turkey: Enhancing food security and sustainable practices for the reduction of CO₂ emissions
Source: PLoS One. 2026 Mar 31;21(3):e0344924. doi: 10.1371/journal.pone.0344924 (PMC13037976; doi:10.1371/journal.pone.0344924)
Supplement: Attachment — Submitted filename: PONE-D-25-27665_R1.docx [file pone.0344924.s009.docx]

Dear Editor,

I am writing to provide my review of the manuscript titled “Exploring Climate Smart Agriculture in Turkey: Enhancing Food Security and Sustainable Practices for the Reduction of CO₂ Emissions”. I’ve reviewed the revised version of your manuscript. Below is my evaluation, focusing on whether the revisions addressed the earlier feedback. I believe that several revisions could improve its clarity, interpretability, and impact.

**Overall Assessment**

The revised draft shows improvement in structure, clarity, and focus compared to the previous version. The abstract is more coherent, the introduction has a clearer flow, and the methodology is presented with more explanation. However, several areas still need refinement.

1. **Abstract:**

- Some sentences remain overly complex, and the quantitative findings are still not clearly explained for a general reader.
- Simplify language and ensure all numerical results are interpreted (e.g., explain what “1% variation in rice cultivation” means in terms of emissions or food security).

2. **Introduction:**

- The narrative could still benefit from smoother transitions between CSA concepts, decoupling methodology, and Turkey’s agricultural context. Some references remain weak or outdated.
- Strengthen the literature review by including more recent peer-reviewed studies on CSA in Turkey or similar economies.

3. **Methodology:**

- Equations and variables are still not fully defined, and terms borrowed from finance (e.g., “portfolio returns”) remain confusing in an agricultural context.
- Provide a clearer justification for why this economic framework is valid for CO₂-agriculture analysis, and ensure all variables are explicitly defined in text or tables.

4. **Results:**

- Interpretation is still sometimes vague, and statistical robustness is not well addressed. Figures and tables (if included) must directly support the discussion.

5. **Discussion:**

- The discussion section reads more like a continuation of results rather than a critical interpretation.
- Emphasize why the results matter in the context of food security and CSA.
- Compare findings explicitly with previous studies.
- Discuss both strengths and limitations.

6. **Conclusion:**

- Still somewhat repetitive, and recommendations remain broad.
- Present three focused take-home messages: (1) empirical finding, (2) policy implication, (3) future research direction.

7. **References:**

- Some non-scholarly or weak sources (e.g., general websites) are still included.
- Replace with peer-reviewed, recent journal articles wherever possible.
