# Supplementary material for: Exploring climate smart agriculture in Turkey: Enhancing food security and sustainable practices for the reduction of CO₂ emissions
Source: PLoS One. 2026 Mar 31;21(3):e0344924. doi: 10.1371/journal.pone.0344924 (PMC13037976; doi:10.1371/journal.pone.0344924)
Supplement: Attachment — Submitted filename: 2nd Response to Reviewers 2025-10-18 (C).docx [file pone.0344924.s010.docx]

**Authors' Response to Reviewers' Comments**

| **Subject:** | PLOS ONE Decision: Revision required [PONE-D-25-27665] |
| --- | --- |

Dear Dr. Khan,

PONE-D-25-27665

Title: **Exploring Climate Smart Agriculture in Turkey: Enhancing Food Security and Sustainable Practices for the Reduction of CO2 Emissions**

Dear editor and reviewers,

Thank you very much for your letter and reviewers’ valuable suggestions on our manuscript entitled “**Exploring Climate Smart Agriculture in Turkey: Enhancing Food Security and Sustainable Practices for the Reduction of CO2 Emissions”** No: [PONE-D-25-27665]. Those comments are precious and helpful for improving our paper and providing the essential guiding significance to our research. We have carefully checked and improved the manuscript according to the editor’s and reviewers’ comments.

**REVIEWER #1:**

**Review Comments to the Author**

**Reviewer #2: In the introduction, the amount of carbon emission from agriculture has not been mentioned. Is it only related with rice field or other crop production?**

**Reply:** I am grateful for your help in obtaining the specified text from the Canvas. We understand that you would like a response to the statement regarding the conceptual and terminological confusion in the manuscript.

The initial paragraph of heading 1 has been revised and updated in relation to the emission of CO2 from the agricultural sector.

**What is the justification of introducing Climate smart agriculture and how it can be done? Moreover, addition of reports on these points is required. Finally, the goal of the study or object is still unclear in abstract, introduction and conclusion. Although there is extensive information on decoupling technique to estimate the portfolio returns and analyze their relationship with climate-smart agriculture.**

**Reply:** I appreciate your assistance in supplying the specified text from the Canvas. We comprehend that you would like a response to the statement concerning the conceptual and terminological confusion in the manuscript.

Including the second and third paragraphs, as well as the first paragraph of the conclusion section, should be included in the updated and changed version of Heading 1.

**Research Methodology:
1. It is not clear why urea application, manure management, and rice cultivation are separately discussed? Is it only for their origin? (in 3.3. Portfolio Returns)**

**Reply:** I appreciate your assistance in supplying the specified text from the Canvas. We comprehend that you would like a response to the statement concerning the conceptual and terminological confusion in the manuscript.

The following is a response that addresses the concerns that were raised:

The heading 3.3 is simply a definition of the portfolio return procedure, and within this heading, in accordance with our methodology 3.1, we have categorised and clearly indicated three sections, such as multiple, single aeration, and continuously followed, where each section is further distributed into two portions: total risk and average return. We have discussed each variable, such as managed soil, field burning, urea application, enteric fermentation, manure management, and rice cultivation, and then we have evaluated the individual outcomes with reference to high danger zones and low risk zones. In last, updated and modified conclusion part heading 1.

**2. “The second-biggest source of carbon dioxide emissions from Turkish CLS-A, after manure management, accounts for about 25% of the overall emissions” is not supported by the given reference.**

**Reply:** I appreciate your assistance in supplying the specified text from the Canvas. We comprehend that you would like a response to the statement concerning the conceptual and terminological confusion in the manuscript.

The following is a response that addresses the concerns that were raised:

The third paragraph of heading 3.3 has been revised and updated with the inclusion of references.

**3. Overall methodology is still confusing.
The result and discussion are very superficial and general. It needs to be more in-depth and better justify the work conducted.**

**Reply:** I appreciate your assistance in supplying the specified text from the Canvas. We comprehend that you would like a response to the statement concerning the conceptual and terminological confusion in the manuscript.

The following is a response that addresses the concerns that were raised:

The methodology section has been revised and updated, particularly in heading 3, where an additional paragraph has been included. Additionally, heading 3.1 has been adjusted to offer additional explanations of three distinct procedures. Further explanation is provided under headings 3.3 (last paragraph have been modified) and 3.4, which provide further information regarding portfolio returns. Furthermore more, the last paragraphs of 4.1 and 4.2 have been updated and modified regarding energy-intensive agriculture and GHG emissions.

**Reviewer #3: The revised manuscript is moving in the right direction and addresses several earlier concerns, particularly in the abstract, introduction, and conclusion. However, further improvements are still needed in methodology clarity, discussion depth, and reference quality.**

**Reply:** I appreciate your assistance in supplying the specified text from the Canvas. We comprehend that you would like a response to the statement concerning the conceptual and terminological confusion in the manuscript.

The following is a response that addresses the concerns that were raised:

Modifications have been made to both the methodology and the discussion section. Some changes have been made to the general methodology section, specifically in heading 3, 3.1, and 3.2. There have been some updates and modifications made to the second paragraph of the discussion section. Individual indicators have been included, and the projected results have been examined further with recent citations.

**Dear Editor,**

**I am writing to provide my review of the manuscript titled “Exploring Climate Smart Agriculture in Turkey: Enhancing Food Security and Sustainable Practices for the Reduction of CO₂ Emissions”. I’ve reviewed the revised version of your manuscript. Below is my evaluation, focusing on whether the revisions addressed the earlier feedback. I believe that several revisions could improve its clarity, interpretability, and impact.**

**Overall Assessment: The revised draft shows improvement in structure, clarity, and focus compared to the previous version. The abstract is more coherent, the introduction has a clearer flow, and the methodology is presented with more explanation. However, several areas still need refinement.**

**Reply:** I appreciate your assistance in supplying the specified text from the Canvas. We comprehend that you would like a response to the statement concerning the conceptual and terminological confusion in the manuscript.

**Reply:** The following is a response that addresses the concerns that were raised:

**Abstract:**

- **Some sentences remain overly complex, and the quantitative findings are still not clearly explained for a general reader.**

**Reply:** Thank you for this essential feedback. I have been thoroughly revised the manuscript to address the clarity issues, focusing specifically on simplifying overly complex sentences and ensuring the quantitative findings are clearly and simply explained for accessibility to a broader audience.

- **Simplify language and ensure all numerical results are interpreted (e.g., explain what “1% variation in rice cultivation” means in terms of emissions or food security).**

**Reply:** Thank you for this excellent and actionable feedback. I have focused my revisions on two critical areas: simplifying the language throughout the text and providing clear interpretations for all numerical results. Specifically, I have ensured that findings like "1% variation in rice cultivation (modified and revised)” are immediately followed by a concrete explanation of what that change signifies for emissions, food security, or other relevant outcomes.

**Introduction:**

- **The narrative could still benefit from smoother transitions between CSA concepts, decoupling methodology, and Turkey’s agricultural context. Some references remain weak or outdated.**

**Reply:** I appreciate your identification of these critical areas for development. I acknowledge that a more comprehensive integration is required to connect the theoretical concepts with the practical application. All of the introduction sections (heading 1), particularly the third paragraph, the first, second, and fourth paragraphs, have been revised and updated. Additionally, the references throughout the entire manuscript were revised.

- **Strengthen the literature review by including more recent peer-reviewed studies on CSA in Turkey or similar economies.**
- **Reply:** Headings 2.1 and 2.2 have been revised and modified, and new and updated references have been added. In order to firmly establish this work inside the present academic landscape, it is vital to strengthen the literature review. This will ensure that the review is up-to-date and provides substantial evidence for our findings.

**Methodology:**

- **Equations and variables are still not fully defined, and terms borrowed from finance (e.g., “portfolio returns”) remain confusing in an agricultural context.**

**Reply:** The initial paragraph of heading 3.4 has been revised and updated to clarify our methodology and terminology. In order to resolve both concerns, we have implemented substantial modifications to Section 3. In which the weighted average return and weighted risk are separately eliminated.

- **Provide a clearer justification for why this economic framework is valid for CO₂-agriculture analysis, and ensure all variables are explicitly defined in text or tables.**

**Reply:** We have incorporated the second paragraph of heading 3.4 into this document. A comprehensive revision has been made to the second paragraph, and the justification has been modified to take into account both the theoretical justification and the definition of variables.

**Results:**

- **Interpretation is still sometimes vague, and statistical robustness is not well addressed. Figures and tables (if included) must directly support the discussion.**

**Reply:** The first, third and fourth paragraphs of (heading 5) of the conclusion (heading 6) section have been updated and changed throughout. In addition to this, the complete article (espcially, 4.1,4.2) has been revised to include individual figures and tables, as well as new references that have been cited.

**Discussion:**

- **The discussion section reads more like a continuation of results rather than a critical interpretation.**

**Reply:** First, third, and fourth paragraphs of (heading 5) have been updated and revised, and new references have been included. In addition we recognise that the preceding discussion section leaned too heavily on merely restating the findings, and we are grateful for the important comments that you provided. A substantial amount of revisions have been made to the discussion in order to transition it from a description.

- **Emphasize why the results matter in the context of food security and CSA.**

**Reply:** A complete revision has been made to heading 5, which is the final paragraph. Taking into consideration the findings of the climate-smart agriculture study conducted in Turkey, which included both high and low risk zones, the agricultural indicators were found to improve food security and sustainable practices for the purpose of reducing carbon dioxide emissions in terms of carbon stockpiles (CLS-A).

- **Compare findings explicitly with previous studies.**

**Reply: Updated and revised the whole discussion portion (Heading 5-first to fifth paragraph).** We have greatly improved the Discussion section to specifically highlight the larger context in response to this insightful criticism. Our study is particularly useful since it stratifies results across high-risk and low-risk zones, going beyond adoption rates to offer evidence-based recommendations on the effectiveness of CLS-A initiatives where they are most required. Our findings support the CLS-A goal by effectively proving that important agricultural indicators improve food security and resilience while also attaining quantifiable decreases in CO2 emissions. This risk-stratified strategy directly and concretely contributes to global climate resilience and food security policy by elucidating the best way to allocate resources and providing a clear path to accomplishing simultaneous adaptation and mitigation targets.

- **Discuss both strengths and limitations.**

**Reply:**

The basic strenght of this study is to computed the risk analysis across high and low risk zone, where the study lie in its unique computation technique and integrated analytical appraoch. This approach provide, empirical evidence in term of CLS-A and it practices the most effective agriculture inidcator under specific environmental consequences. In addition, it integrate and quantify the CLS-A by agriculture productivity and resiliences with counter measurable reduction of enviormental pressures-CO2 emissions. This study also provide roboust quantative validation of CLS-A concept in different groups. Howerver, the limiation of study is indicated that reliance on cross-sectional data, where this study robust casual relationaships between CLS-A outcomes and we cannot fully measure the track changes in CO2 emisison sequestration with soil management next multple years. In addition, there are no basis analysis of cover cropping or farming, it may overlape the net GHG footprint of the Turkey agriculture system.

**Conclusion:**

- **Still somewhat repetitive, and recommendations remain broad.**

**Reply:** Modified and updated the entire recommendation portion (Heading 6) first paragarph.

- **Present three focused take-home messages: (1) empirical finding, (2) policy implication, (3) future research direction.**

**Reply:** Both the second and fourth paragraphs have been altered and updated in accordance with the empirical findings and the future research that is presented in heading 6.

**References:**

- **Some non-scholarly or weak sources (e.g., general websites) are still included.**

**Reply:** All of the references have been revised and up to date, and the manuscript has been removed from the poor sources.

- **Replace with peer-reviewed, recent journal articles wherever possible.**

**Reply:** Review and update all references, paying particular attention to replacing peer-reviewed material.

Finally, thank you so much for your helpful advice and invaluable ostentation. I'm speechless in the face of your vast knowledge and impressive list of publications, but I can attest to the fact that this manuscript benefits greatly from your fertile ostentation.

**Once again, I greatly appreciate your insightful feedback and recommendations.**
