# Supplementary material for: Exploring climate smart agriculture in Turkey: Enhancing food security and sustainable practices for the reduction of CO₂ emissions
Source: PLoS One. 2026 Mar 31;21(3):e0344924. doi: 10.1371/journal.pone.0344924 (PMC13037976; doi:10.1371/journal.pone.0344924)
Supplement: Attachment — Submitted filename: PONE-D-25-27665_R2.docx [file pone.0344924.s011.docx]

Dear Editor,

I am writing to provide my review of the manuscript titled “Exploring Climate Smart Agriculture in Turkey: Enhancing Food Security and Sustainable Practices for the Reduction of CO₂ Emissions“. The authors have thoroughly and effectively revised the manuscript in response to previous review comments. The current version shows clear and substantial improvements in structure, clarity, methodological transparency, and scholarly rigor. The revised abstract, introduction, and discussion are now coherent and aligned with the stated objectives, while the methodology and results sections demonstrate a better balance between economic modeling and agricultural relevance.

Before final acceptance I suggest three minor actions:

1. Minor language refinement

- While the overall English has improved, a final light proofread is recommended to correct minor grammatical inconsistencies and occasional redundancy in phrasing (e.g., repeated expressions in the Introduction and Discussion).

2. Clarity in figures and tables

- Perform a final, quick pass to harmonize formatting and labels (figure/table captions and in-text references) and to ensure consistent use of key terms (for example “trade-in” and “blockchain adoption”).

3. Conclusion structure

- The revised conclusion is more coherent; however, the section would benefit from three concise closing points summarizing:

1. the empirical contribution (quantitative evidence of CSA–CO₂ linkage),
2. the policy implication for sustainable agricultural transition in Turkey, and
3. a brief future research direction emphasizing cross-country validation of the decoupling model.
