# Supplementary material for: Exploring climate smart agriculture in Turkey: Enhancing food security and sustainable practices for the reduction of CO₂ emissions
Source: PLoS One. 2026 Mar 31;21(3):e0344924. doi: 10.1371/journal.pone.0344924 (PMC13037976; doi:10.1371/journal.pone.0344924)
Supplement: Attachment — Submitted filename: 3rd Response to Reviewers 2025-12-7 (A).docx [file pone.0344924.s012.docx]

**Authors' Response to Reviewers' Comments**

| **Subject:** | PLOS ONE Decision: Revision required [PONE-D-25-27665] |
| --- | --- |

Dear Dr. Khan,

PONE-D-25-27665

Title: **Exploring Climate Smart Agriculture in Turkey: Enhancing Food Security and Sustainable Practices for the Reduction of CO2 Emissions**

Dear editor and reviewers,

Thank you very much for your letter and reviewers’ valuable suggestions on our manuscript entitled “**Exploring Climate Smart Agriculture in Turkey: Enhancing Food Security and Sustainable Practices for the Reduction of CO2 Emissions”** No: [PONE-D-25-27665]. Those comments are precious and helpful for improving our paper and providing the essential guiding significance to our research. We have carefully checked and improved the manuscript according to the editor’s and reviewers’ comments.

**REVIEWER #2:**

**The authors have not addressed all the points raised in the previous revision, especially the result and discussion.**

**Reply:** I appreciate your assistance in retrieving the specified text from Canvas. We acknowledge your request for a response concerning the statement addressing the conceptual and terminological ambiguities within the manuscript.

I have made revisions to the entire results and discussion section of the paper, taking into consideration the remarks that were made by the most recent reviewer. The findings section 4 of the current manuscript has been completely revised, with the most notable change being the addition of three subheadings to heading 4.1. In addition, three subheadings have been added to heading 4.2 in the same situation. In this particular situation, you should classify CLS-A signs as either high risk or low risk in each subsection separately. Additionally, you should provide a detailed explanation of each signal in a very succinct manner. (Refer to sections 4.1.1 through 4.1.3 and 4.2.1 through 4.2.3.) In relation to the discussion section (5), it has also been altered. It consists of eight paragraphs in total that address all of the subheadings, and it includes a quick interpretation of the consequences of all of the indicators.

Once more, I would like to express my gratitude for the time that you have donated to this process and for your direction throughout its duration. In closing, I would like to express my heartfelt gratitude for the advice that you have given me, which has been very beneficial, as well as the important support that you have provided. The sheer magnitude of your knowledge and the remarkable collection of publications that you have produced leave me completely speechless; nonetheless, I can attest to the fact that this text substantially benefits from the productivity of your display of writing.

**REVIEWER #3:**

**Dear Editor,**

**I am writing to provide my review of the manuscript titled “Exploring Climate Smart Agriculture in Turkey: Enhancing Food Security and Sustainable Practices for the Reduction of CO₂ Emissions“. The authors have thoroughly and effectively revised the manuscript in response to previous review comments. The current version shows clear and substantial improvements in structure, clarity, methodological transparency, and scholarly rigor. The revised abstract, introduction, and discussion are now coherent and aligned with the stated objectives, while the methodology and results sections demonstrate a better balance between economic modeling and agricultural relevance.**

**Before final acceptance I suggest three minor actions:**

**Minor language refinement**

**While the overall English has improved, a final light proofread is recommended to correct minor grammatical inconsistencies and occasional redundancy in phrasing (e.g., repeated expressions in the Introduction and Discussion).**

**Reply:** I appreciate your assistance in retrieving the specified text from Canvas. We acknowledge your request for a response concerning the statement addressing the conceptual and terminological ambiguities within the manuscript.

We conducted a thorough and final review of the manuscript. This action addressed and rectified minor grammatical inconsistencies and eliminated instances of redundancy, especially within the Introduction and Discussion sections, to maintain clear and concise language throughout the document.

**Clarity in figures and tables**

**Perform a final, quick pass to harmonize formatting and labels (figure/table captions and in-text references) and to ensure consistent use of key terms (for example “trade-in” and “blockchain adoption”).**

**Reply:** I appreciate your assistance in retrieving the specified text from Canvas. We acknowledge your request for a response concerning the statement addressing the conceptual and terminological ambiguities within the manuscript.

We performed a comprehensive review to standardize all formatting and labels. This process entailed maintaining uniform presentation across all figure and table captions, verifying the accuracy of in-text references, and ensuring the consistent use of essential technical terminology (e.g., consistently employing "trade-in" and "blockchain adoption" throughout the document).

**Conclusion structure**

**The revised conclusion is more coherent; however, the section would benefit from three concise closing points summarizing:**

**the empirical contribution (quantitative evidence of CSA–CO₂ linkage),**

**the policy implication for sustainable agricultural transition in Turkey, and a brief future research direction emphasizing cross-country validation of the decoupling model.**

**Reply:** I appreciate your assistance in retrieving the specified text from Canvas. We acknowledge your request for a response concerning the statement addressing the conceptual and terminological ambiguities within the manuscript.

We have refined the Conclusion section to enhance its effectiveness by including three succinct closing statements that clearly encapsulate the primary contributions of the study: The Empirical Contribution: We emphasized the quantitative evidence demonstrating the substantial relationship between CSA practices and the decoupling of CO₂ emissions in the agricultural sector. The Policy Implication: We delivered a practical recommendation for advancing sustainable agriculture in Turkey, emphasizing the importance of strategic policy support. Future Research Direction: We conclude with a concise and targeted recommendation highlighting the importance of conducting cross-national validation of the proposed decoupling model. These concluding revisions conclude the review process, and we are confident that the manuscript is now prepared for publication.

Thank you once again for your valuable time and guidance throughout this process. Finally, thank you so much for your helpful advice and invaluable assistance. I'm speechless in the face of your vast knowledge and impressive list of publications, but I can attest to the fact that this manuscript benefits greatly from your fertile ostentation.

**Once again, I greatly appreciate your insightful feedback and recommendations.**
