# Supplementary material for: Exploring climate smart agriculture in Turkey: Enhancing food security and sustainable practices for the reduction of CO₂ emissions
Source: PLoS One. 2026 Mar 31;21(3):e0344924. doi: 10.1371/journal.pone.0344924 (PMC13037976; doi:10.1371/journal.pone.0344924)
Supplement: Attachment — Submitted filename: Reviewer comments.docx [file pone.0344924.s004.docx]

1. My main comments are mentioned on the manuscript PDF file.
2. Abstract: The abstract provides a concise overview of the study, highlighting the main objectives and significant findings. The abstract could benefit from a brief mention of the broader implications of these findings for different crop production.
3. Introduction: Minimize repetitive statements, focus more on the novelty of the study, reducing excessive detail on general information.
4. Methodology: Elaborate the methodology to make it easy to understand. Make it more logical and robust.
5. Data Interpretation in Results: The results section presents extensive data but lacks in-depth interpretation and discussion of these findings within the section, missing an opportunity to provide insights into the implications of these results.
6. Conclusion: The conclusion succinctly summarizes the findings. But it could be enhanced by providing specific recommendations for future research.

In summary, while the manuscript presents a valuable study with significant implications for the Climate Smart Agriculture in Turkey, addressing these critical flaws would strengthen the manuscript's contribution to the field.
